# Omega-3 polyunsaturated fatty acids protect against inflammation through production of LOX and CYP450 lipid mediators: relevance for major depression and for human hippocampal neurogenesis

**DOI:** 10.1038/s41380-021-01160-8

**Published:** 2021-06-16

**Authors:** Alessandra Borsini, Anna Nicolaou, Dolores Camacho-Muñoz, Alexandra C. Kendall, Maria Grazia Di Benedetto, Juliette Giacobbe, Kuan-Pin Su, Carmine M. Pariante

**Affiliations:** 1 Stress, Psychiatry and Immunology Laboratory, Institute of Psychiatry, Psychology and Neuroscience, Department of Psychological Medicine, King’s College London, London, UK; 2 Laboratory for Lipidomics and Lipid Biology, Division of Pharmacy and Optometry, School of Health Sciences, Faculty of Biology, Medicine and Health, The University of Manchester, Manchester, UK; 3 Lydia Becker Institute of Immunology and Inflammation, Faculty of Biology, Medicine and Health, The University of Manchester, Manchester, UK; 4 Biological Psychiatry Unit, IRCCS Istituto Centro San Giovanni di Dio, Fatebenefratelli, Brescia, Italy; 5 College of Medicine, China Medical University, Taichung, Taiwan; 6 Depression Center, An-Nan Hospital, China Medical University, Tainan, Taiwan

**Keywords:** Cell biology, Neuroscience, Molecular biology

## Abstract

Eicosapentaenoic acid (EPA) and docosahexaenoic acid (DHA) can exert antidepressant, anti-inflammatory and neuroprotective properties, but the exact molecular mechanism underlying their effects is still not fully understood. We conducted both in vitro and clinical investigations to test which EPA or DHA metabolites are involved in these anti-inflammatory, neuroprotective and antidepressant effects. In vitro, we used the human hippocampal progenitor cell line HPC0A07/03C, and pre-treated cells with either EPA or DHA, followed by interleukin 1beta (IL1β), IL6 and interferon-alpha (IFN-α). Both EPA and DHA prevented the reduction in neurogenesis and the increase in apoptosis induced by these cytokines; moreover, these effects were mediated by the lipoxygenase (LOX) and cytochrome P450 (CYP450) EPA/DHA metabolites, 5-hydroxyeicosapentaenoic acid (HEPE), 4-hydroxydocosahexaenoic acid (HDHA), 18-HEPE, 20-HDHA, 17(18)-epoxyeicosatetraenoic acid (EpETE) and 19(20)-epoxydocosapentaenoic acid (EpDPA), detected here for the first time in human hippocampal neurones using mass spectrometry lipidomics of the supernatant. In fact, like EPA/DHA, co-treatment with these metabolites prevented cytokines-induced reduction in neurogenesis and apoptosis. Moreover, co-treatment with 17(18)-EpETE and 19(20)-EpDPA and the soluble epoxide hydroxylase (sEH) inhibitor, TPPU (which prevents their conversion into dihydroxyeicosatetraenoic acid (DiHETE)/ dihydroxydocosapentaenoic acid (DiHDPA) metabolites) further enhanced their neurogenic and anti-apoptotic effects. Interestingly, these findings were replicated in a sample of *n* = 22 patients with a DSM-IV Major Depressive Disorder, randomly assigned to treatment with either EPA (3.0 g/day) or DHA (1.4 g/day) for 12 weeks, with exactly the same LOX and CYP450 lipid metabolites increased in the plasma of these patients following treatment with their precursor, EPA or DHA, and some evidence that higher levels of these metabolites were correlated with less severe depressive symptoms. Overall, our study provides the first evidence for the relevance of LOX- and CYP450-derived EPA/DHA bioactive lipid metabolites as neuroprotective molecular targets for human hippocampal neurogenesis and depression, and highlights the importance of sEH inhibitors as potential therapeutic strategy for patients suffering from depressive symptoms.

## Introduction

Evidence suggests that at around 30% of patients with depression do not respond to antidepressant treatment, with most of them having sub-chronic levels of inflammation [1–3], a process which potentially impacts depression-relevant brain pathways. However, despite the role of inflammation in depression, there is still a lack of anti-inflammatory strategies that are effective for these patients, safe for everyday use, and with a clear mechanism of action. Nutrition-based therapeutic strategies consisting of the omega-3 polyunsaturated fatty acids (ω-3 PUFAs), eicosapentaenoic acid (EPA) and docosahexaenoic acid (DHA) are considered promising therapeutic approaches [4, 5]. Indeed, clinical studies within our and other laboratories have showed that diets rich in ω-3 PUFAs, such as EPA and DHA provide beneficial anti-inflammatory and anti-depressant effects [6–12]. Moreover, we have also previously demonstrated that in vitro treatment of human hippocampal progenitors with EPA and DHA can prevent reduction in neurogenesis caused by IL1β, much like treatment with antidepressants, sertraline and venlafaxine, does [13]. However, the exact molecular mechanism by which ω-3 PUFAs exert their anti-inflammatory and anti-depressant effects is currently unknown.

This ability of PUFAs to protect neurones from the detrimental effects of inflammation is likely to be relevant for their antidepressant action, at least in the sub-group of patients with major depression characterised by immune dysregulation [6–12]. In particular, activation of the inflammatory response system in these patients is characterised by an increase in the production of inflammatory cytokines, including interleukin-1beta (IL1β) and IL6 both in the periphery and in the cerebrospinal fluid [1, 14]. These findings are of fundamental importance as cytokines can directly contribute to the development of the depressive symptoms [15]. Indeed, increased levels of cytokines circulating in the periphery can penetrate the more permeable areas of the blood-brain barrier (BBB) to affect brain signalling relevant for the depressive symptoms [16]. In particular, cytokines have been shown to alter neurogenesis, a mechanism potentially disrupted in depression, and required for antidepressant efficacy [17–19]. Indeed, using the aforementioned human hippocampal progenitor cells, we have previously demonstrated the ability of IL1β, IL6 and interferon-alpha (IFN-α) to cause reduction in cell proliferation and neurogenesis, and increased apoptosis, via activation of the downstream inflammatory signalling pathways, transcription factor signal transducer and activator of transcription 1 (STAT1) and nuclear factor-kappa B (NF-kB) [13, 20, 21].

**Fig. 1:**
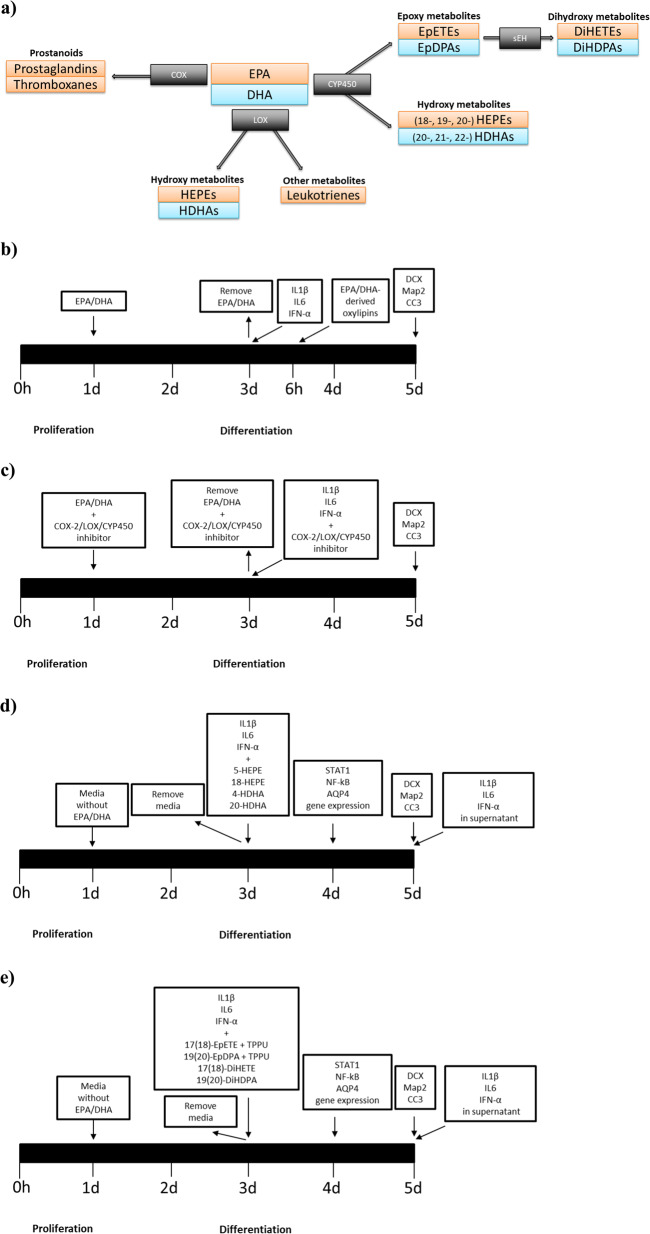
Enzymatic synthesis pathways of ω-3 PUFAs and timeline of experiments with ω-3 PUFAs and derived lipid mediators. **a** ω-3 PUFAs are metabolised by COX, LOX and CYP450 enzymes. COX and LOX enzymes convert ω-3 PUFAs into prostanoids, hydroxy fatty acids and leukotrienes, while CYP450 monooxygenases convert ω-3 PUFAs into epoxy and hydroxy fatty acids. Epoxy fatty acids are then metabolised via the sEH enzyme to the corresponding dihydroxy metabolites. **b** HPC0A07/03C cells were plated for 24 h in proliferating media and then pre-treated with either EPA or DHA for 48h in differentiating media. After 48 h, EPA or DHA were removed and treatment with cytokines IL1β, IL6 or IFN-α was added for additional 6 or 48 h. At the end of the 6h period, supernatant was collected, and metabolites were analysed. Whereas, at the end of the 48 h period, cells were fixed and stained for markers of neurogenesis (DCX and Map2) and apoptosis (CC3). **c** In another experiment, cells were pre-treated with EPA or DHA and an inhibitor of COX-2 inhibitor (CAS 416901-58-1), LOX enzymes (NDGA) or CYP450 enzymes (SKF525A) for 48 h, followed by 48h treatment with cytokines (IL1β, IL6 or IFN-α) and again the inhibitors (COX-2, LOX or CYP450 inhibitor). Cells were then fixed and stained for markers of neurogenesis (DCX and Map2) and apoptosis (CC3). **d** Cells were plated for 24h in proliferating media, and then differentiated for 48 h in vehicle condition without any treatment. Media was then changed and treatment with cytokines (IL1β, IL6 or IFN-α) and EPA- or DHA-derived metabolites (5-HEPE, 18-HEPE, 4HDHA or 20-HDHA) were added for additional 6h or 48h. At the end of the 6h period mRNA was extracted for gene expression of STAT1, NF-kB and AQP4. Whereas, at the end of the 48 h period, cells were fixed and stained with DCX, Map2 and CC3, and supernatant was collected for cytokines analysis. **e** Cells were plated for 24h in proliferating media, and then differentiated for 48 h in vehicle condition without any treatment. Media was then changed and treatment with cytokines (IL1β, IL6 or IFN-α) and EPA- or DHA-derived metabolites (17(18)-EpETE, 19(20)-EpDPA, 17(18)-DiHETE, 19(20)-DiHDPA), with or without TPPU, were added for additional 6 or 48 h. At the end of the 6h period mRNA was extracted for gene expression of STAT1, NF-kB and AQP4. Whereas, at the end of the 48 h period, cells were fixed and stained with DCX, Map2 and CC3, and supernatant was collected for cytokines analysis. Legend: EPA, eicosapentaenoic acid; DHA, docosahexaenoic acid; COX-2, cyclooxygenase-2; LOX, lipoxygenase; CYP450, cytochrome P450; HEPE, hydroxyeicosapentaenoic acid; HDHA, hydroxydocosahexaenoic acid; EpETE, epoxydocosapentaenoic acid; EpDPA, epoxydocosapentaenoic acid; DiHETE, dihydroxyeicosatetraenoic acid; DiHDPA, dihydroxydocosapentaenoic acid; sEH, soluble epoxide hydroxylase; IL1β, interleukin 1beta; IL6, interleukin 6; IFN-α, interferon-alpha; DCX, doublecortin; Map2, microtubule-associated protein 2; CC3, caspase 3; STAT1, activator of transcription 1; NF-kB, nuclear factor-κB; AQP4, aquaporin 4.

One molecular mechanism potentially involved in the antidepressant effects of PUFAs is the action of their metabolites. Polyunsaturated fatty acids are metabolised by cyclooxygenase (COX), lipoxygenase (LOX) and cytochrome P450 (CYP450) enzymes into a range of lipid mediators, which exhibit potent immune regulatory activities [22] (see Fig. 1a). COX and LOX enzymes convert ω-3 PUFAs into prostanoids, mono- and polyhydroxy fatty acids and leukotrienes, while CYP450 monooxygenases convert ω-3 PUFAs into epoxy and hydroxy fatty acids [23]. Epoxy fatty acids are then metabolised via epoxide hydrolases (primarily soluble epoxide hydrolase (sEH)) to the corresponding fatty acyl diols [22]. Although previous studies have shown the ability of lipid mediators to exert anti-inflammatory and neurogenic properties in pre-clinical models of depression, these investigations mainly focussed on specific LOX-derived metabolites [24, 25], the specialised pro-resolving mediators, like resolvins, protectins and maresins [26, 27]. However, the role of other classes of ω-3 lipid metabolites, and their individual involvement in the neuroprotective and antidepressants effects of ω-3 PUFAs, are yet to be investigated. Indeed, there is no clear evidence that such metabolites are produced in human hippocampal neurons, and, in clinical context, only one small, exploratory study found that, in nine depressed patients, seasonal changes in depressive symptoms correlated with changes in some ω-3 lipids metabolites [28].

Considering the limited evidence for the role of these metabolites in the mechanism underpinning the anti-inflammatory and neurogenic activities of ω-3 PUFAs in the context of depression, we used our aforementioned, validated in vitro model of ‘depression in a dish’ [13, 20, 21, 29–33] exposing immortalised human hippocampal progenitor cell line HPC0A07/03C to candidate ‘depressogenic’ cytokines, IL1β, IL6 and IFN-α, resulting in a reduction in neurogenesis and an increase in neuronal apoptosis [13, 20, 21]. In order to identify the lipid species or families of COX-, LOX- or CYP450-derived mediators putatively involved in the anti-inflammatory and neuroprotective effects exerted by ω-3 PUFAs, we pre-treated cells with EPA/DHA with or without a selective inhibitor of COX-, LOX- or CYP450 enzymes, and then directly exposed cells to candidate metabolites. Our hypothesis was that PUFAs metabolites are produced by neurones in vitro and mediate the protective effects of EPA and DHA on inflammation-induced reduction in neurogenesis and increase in apoptosis. Moreover, we validated our pre-clinical findings in a *clinical cohort* of depressed patients receiving a 12 weeks nutritional intervention with EPA or DHA. Our hypothesis was that the same lipid mediators identified as mechanistically relevant in the in vitro studies are increased in plasma samples after ω-3 PUFA treatment, and that this increase is associated with a reduction in the depressive symptomatology.

## Methods

### Cell culture

Multipotent human hippocampal progenitor cell line HPC0A07/03C (provided by ReNeuron, Surrey, UK) was used [13, 30, 34–37]. This model has been previously validated using a hippocampal newborn neuron specific marker, Prospero homeobox protein 1 [30]. Cells were left to proliferate in Dulbecco’s Modified Eagle Medium: Nutrient Mixture F-12 (DMEM/F-12) media to which we added the growth factors epidermal growth factor, basic fibroblast growth factor and 4-hydroxytamoxifen (4-OHT). Differentiation was initiated by removal of the growth factors and 4-OHT. Detailed information on this cell line can be found in our previous publication [30].

### In vitro treatment with ω-3 PUFAs, lipid metabolites and cytokines

Cells were plated on 96 well plates (Nunclon) at a density of 1.5 × 10^4^ cells per well. After 1d proliferation, cells were left to differentiate for a total of 4d. Differentiated cells were pre-treated with either EPA or DHA (both 10 µM), with/without COX-2 inhibitor (CAS 416901-58-1; 1, 4 and 8 µM), LOX inhibitor nordihydroguaiaretic acid (NDGA; 1, 10 and 30 µM) or CYP450 inhibitor (SKF525A; 1 µM) for 2d, followed by treatment with either IL1β (10000 pg/ml), IL6 (50 pg/ml) or IFN-α (50,000 pg/ml), with/without NDGA or SKF525A or CAS 416901-58-1, for additional 6h, 1d or 2d (Fig. 1b, c, further details in Supplementary Materials). Cells were then pre-treated with media (without EPA or DHA) for 2d, followed by co-treatment with 5-hydroxyeicosapentaenoic acid (HEPE) (1500–3000pg/ µl), 18-HEPE (4000–8000 pg/µl), 4-hydroxydocosahexaenoic acid (HDHA), 20-HDHA (both 1500–3000 pg/µl), 17(18)-epoxyeicosatetraenoic acid (EpETE) (0.04–0.08 pg/µl), 19(20)-epoxydocosapentaenoic acid (EpDPA) (0.15–0.3 pg/µl), 17(18)-dihydroxyeicosatetraenoic acid (DiHETE) (1000–2000 pg/µl) or 19(20)-dihydroxydocosapentaenoic acid (DiHDPA) (0.3–0.6 pg/µl), and either IL1β, IL6 or IFN-α (concentrations as above) for additional 2 days, with or without sEH inhibitor TPPU (1 nM) (Fig. 1d, e). At the end of the total period of differentiation (4 days), cells were rinsed with warm PBS and fixed with 4% PFA for 20 min at room temperature (Fig. 1b, c, d, e), whereas supernatant was collected for subsequent cytokines measurement (Fig. 1d, e).

### Immunocytochemistry and quantification of immunofluorescence

Fixed cells were stained for markers of immature and mature neurons using respectively doublecortin (DCX; Alexa 488 donkey anti-rabbit; 1:1000, Invitrogen) and microtubule-associated protein 2 (Map2; Alexa 555 donkey anti-mouse, 1:1000, Invitrogen), whereas apoptotic cells were examined using caspase 3 (CC3; Alexa 555 donkey anti-rabbit, 1:1000, Invitrogen). All cells were labelled using DAPI dye, as in previous publications [13, 20, 21] (see also Supplementary Materials). The number of DCX and Map2 positive cells over total DAPI positive cells was counted using an insight automated imaging platform (CellInsight) (Supplementary Fig. 1 for representative images).

### Singleplex cytokine measurement

Cell supernatants were used for cytokines measurement (IL1β, IL6 or IFN-α), using the Human ProInflammatory Singleplex Very-Sensitive Kit from Meso Scale Discovery (MSD) (Gaithersburg, MD) and the SECTOR Imager MSD device, according to the manufacturers’ instructions. Detailed information on the cytokines analyses procedure can be found in our previous publication [21].

### Analysis of lipid mediators in cell culture supernatants

Cells were plated on six well plates (Nunclon) at a density of 4.5 × 10^5^ cells per well. After 2 days pre-treatment with either EPA or DHA (both 10 µM), followed by 6 h treatment with either IL1β, IL6 or IFN-α (concentrations as above) supernatant was collected, and a panel of ω-3 and ω-6 PUFAs metabolites were measured with ultraperformance liquid chromatography with electrospray ionisation and tandem mass spectrometry (UPLC/ESI-MS/MS), as reported in our previous publication [38] (see also Supplementary Materials, and Supplementary Fig. 2a for the full panel of metabolites analysed).

### RNA isolation and quantitative real-time PCR (qRT-PCR) analysis

Cells were plated on six well plates (Nunclon) at a density of 4.5  × 10^5^ cells per well. After 2d pre-treatment with media (without EPA or DHA), followed by 1 day co-treatment with EPA- or DHA-derived lipid metabolites and either IL1β, IL6 or IFN-α (concentrations as above), RNA was isolated using RNeasy Plus Micro Kit (Quiagen) and both target (STAT1, NF-kB and aquaporin 4 (AQP4)) and housekeeping genes (ribosomal protein L13A and beta-actin) expression levels were analysed by TaqMan qRT-PCR instrument (CFX384 real time system, Bio-Rad, California, USA), as reported in our previous publication [20] (see also Supplementary Materials).

### Clinical samples

The sub-group of patients included in this study was recruited from the outpatient Psychiatric Department at the China Medical University Hospital, Taichung, Taiwan, ROC [39]. This study was approved by the Institutional Review Board, and it was also registered at Clinical-Trials.gov (NCT03871088). Eligible patients were those who met the diagnostic criteria of DSM-IV for MDD, and had baseline scores of 18 or greater on the 21-item Hamilton Rating Scale for Depression [40]. Informed consent was obtained from all subjects. A total of 22 MDD patients were randomly assigned to treatment with either EPA (3.0 g/day) or DHA (1.4 g/day) for 12 weeks (*n* = 11 per group).

### Analysis of lipid mediators in plasma

Plasma samples from the 22 patients (*N* = 11 per group, EPA or DHA) were collected at baseline (before treatment with EPA or DHA) and at week 12, and, as for cell supernatant, lipid mediators were measured using the same UPLC/ESI-MS/MS [38] apparatus and in the same laboratory as used in the in vitro study (see Supplementary Materials, Supplementary Fig. 2a).

### Statistical Analysis

Statistical analyses were performed with IBM SPSS statistical software version 25, StataCorp STATA version 16 and GraphPad Prism version 8 and consisted of one-way, two-way analysis of variance, Chi-square *χ*^2^ test, Mann Whitney *U*, Wilcoxon and Spearman’s *r*_s_ test, followed by Bonferroni’s post hoc analyses where appropriate. Variance was similar between the groups that have been statistically compared. Data are presented as mean ± SEM, and *p* values ≤ 0.05 were considered significant.

## Results

### The ability of EPA and DHA to prevent cytokines-induced reduction in neurogenesis and increase in apoptosis is mediated by production of LOX and CYP450 lipid mediators

**Fig. 2:**
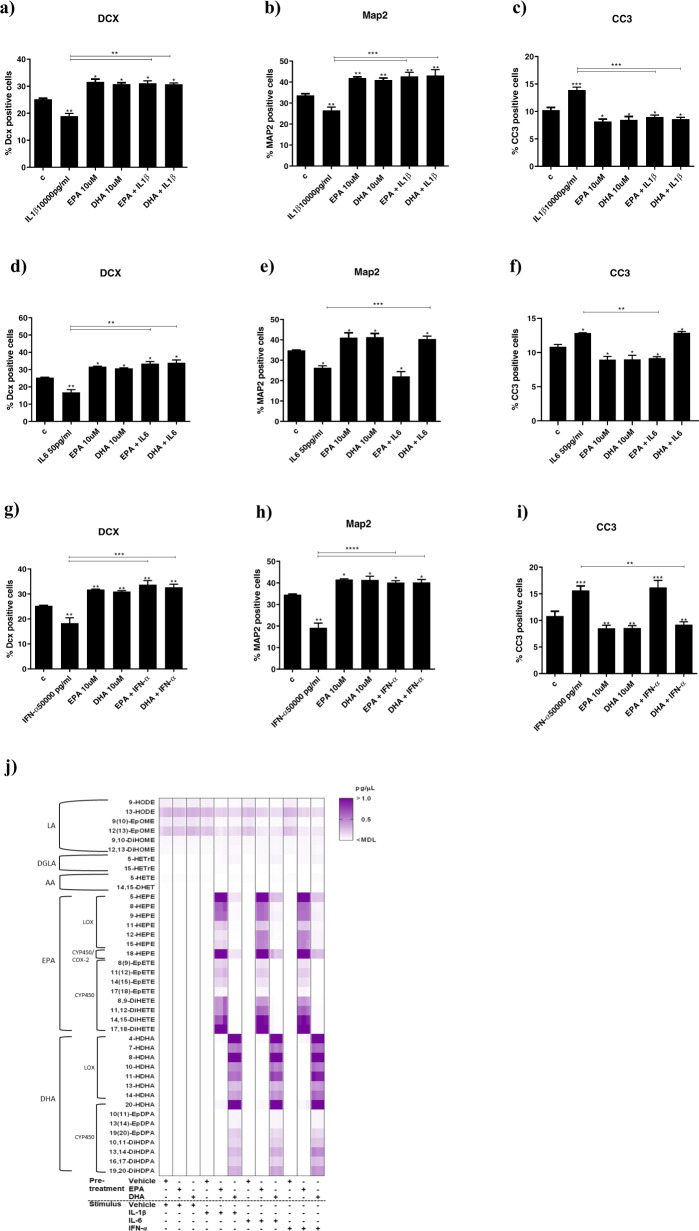
Pre-treatment with EPA or DHA prevents cytokines-induced reduction in neurogenesis and increase in apoptosis, and stimulates the production of EPA- and DHA-derived LOX and CYP450 lipid mediators. **a–i** Pre-treatment of cells with either EPA or DHA followed by IL1β, IL6 or IFN-α prevented the reduction in neurogenesis (DCX+ and Map2+ cells) and/or increase in apoptosis (CC3+ cells) induced by the cytokines alone. Two-way ANOVA with Bonferroni’s post hoc test was performed. Data are shown as mean ± SEM; *p < 0.05, **p < 0.01, ***p < 0.001, ****p < 0.0001, compared with vehicle treatment or as indicated. **j** Heatmap showing metabolites production in supernatant of cells pre-treated for 48 h during differentiation with either EPA or DHA, followed by 6 h treatment with cytokines (IL1β, IL6 or IFN-α). Legend: LA, linoleic acid; DGLA, dihomo-γ-linolenic acid; AA, arachidonic acid; EPA, eicosapentaenoic acid; DHA, docosahexaenoic acid; COX-2, cyclooxygenase-2; LOX, lipoxygenase; CYP450, cytochrome P450; HODE, hydroxyoctadecadienoic acid; EpOME, epoxyoctadecenoic acid; DiHOME, dihydroxyoctadecenoic acid; HETrE, hydroxyeicosatrienoic acid; HETE, hydroxyeicosatetraenoic acid; DHET, dihydroxyeicosatrienoic acid; HEPE, hydroxyeicosapentaenoic acid; EpETE, epoxyeicosatetraenoic acid; DiHETE, dihydroxyeicosatetraenoic acid; HDHA, hydroxydocosahexaenoic acid; EpDPA, epoxydocosapentaenoic acid; DiHDPA, dihydroxydocosapentaenoic acid.

As first step, we extended our initial observation that EPA and DHA prevent IL1β -induced reduction in neurogenesis and increase in apoptosis to two other candidate ‘depressogenic’ cytokines, IL6 and IFN-α. Cell were pre-treated with EPA or DHA (both 10 µM) for 2 days during differentiation, followed by treatment with either IL1β (10,000 pg/ml), IL6 (50 pg/ml) or IFN-α (50,000 pg/ml) for additional 2 days (Fig. 1b), as in our previous studies with PUFAs and with IL1β [13, 41]. Not only we were able to independently replicate the previously described ability of either EPA and DHA to prevent the IL1β-induced reduction in DCX+ cells (Fig. 2a) and in Map2+cells Fig. 2b), and increase in CC3+cells (Fig. 2c), but also we found similar (but more specific) effects with the other cytokines. Indeed, we found that EPA or DHA also prevented reduction in DCX+ cells caused by treatment with IL6 alone (Fig. 2d), while only DHA prevented the reduction in Map2+ cells (Fig. 2e), and only EPA prevented the increase in CC3+ cells (Fig. 2f). Moreover, pre-treatment with EPA or DHA prevented reduction in DCX+ cells (Fig. 2g) and Map2+cells caused by treatment with IFN-α alone (Fig. 2h), while only DHA prevented the increase in CC3+ cells (Fig. 2i).

In order to test whether ω-3 PUFAs metabolites are produced in human hippocampal neurones, and if they are involved in these protective effects of neurogenesis and apoptosis we pre-treated cell with either EPA or DHA (both 10 µM) for 2 days during differentiation, followed by treatment with either IL1β, IL6 or IFN-α (concentrations as above) for additional 6 h (Fig. 1b), then measured a panel of EPA- and DHA-derived lipid mediators in cell supernatant (Supplementary Fig. 2a). In cells pre-treated with EPA, we found an increase in EPA-derived LOX (5-, 8-, 9-, 11-, 12-, 15-HEPE), CYP450 *hydroxylase*/COX-2 (18-HEPE) and CYP450 *epoxygenase* (8(9)-, 11(12)-, 14(15)-, 17(18)-EpETE and 8(9)-, 11(12)-, 14(15)-, 17(18)-DiHETE) species; and, in cells pre-treated with DHA, we found an increase in DHA-derived LOX (4-, 7-, 8-, 10-, 11-, 13-, 14-HDHA), CYP450 *hydroxylase* (20-HDHA) and CYP450 *epoxygenase* (10(11)-, 13(14)-, 19(20)-EpDPA and 10(11)-, 13(14)-, 16(17)-, 19(20)-DiHDPA) species (see Fig. 2j and example chromatograms in Supplementary Fig. 2b–i).

Then, in order to understand whether these metabolites are involved in the effect exerted by ω-3 PUFAs, cells were pre-treated with either EPA or DHA with or without a selective inhibitor for COX-2 enzyme (CAS 416901-58-1; 1 µM), LOX (NDGA; 1 µM) or CYP450 (SKF525A; 1 µM) for 2 days during differentiation, followed by treatment with either IL1β, IL6 or IFN-α, again with or without LOX, CYP450 or COX-2 inhibitors, for additional 2d (Fig. 1c). While treatment with COX-2 inhibitor did not affect results (see Supplementary Fig. 3a–i and Supplementary Materials), reatment with LOX or CYP450 inhibitors prevented the protective effect of EPA or DHA against cytokines-induced reduction in neurogenesis and increase in apoptosis (see Supplementary Fig. 4a–r and Supplementary Materials).

### Treatment with LOX or CYP450 lipid mediators prevents cytokine-induced reduction in neurogenesis and increase in apoptosis

As our results using enzyme inhibitors indicated that lipids oxidised via LOX or CYP450 enzymes are involved in the anti-inflammatory and neurogenic effects of EPA and DHA, we exposed cells to co-treatment with the most highly produced LOX or CYP450 lipid mediators and either IL1β, IL6 or IFN-α (as above) for 2d (see Fig. 1d, e). We used two concentrations for each lipid: the concentration detected at the end of the incubation period (see Fig. 2j), and then a higher concentration (twice the concentration detected at the end of the incubation period) to account for the possibility that the ‘biologically active’ levels of some metabolites might have been reached earlier during the incubation, with lower levels detected at the end of the incubation period.

**Fig. 3:**
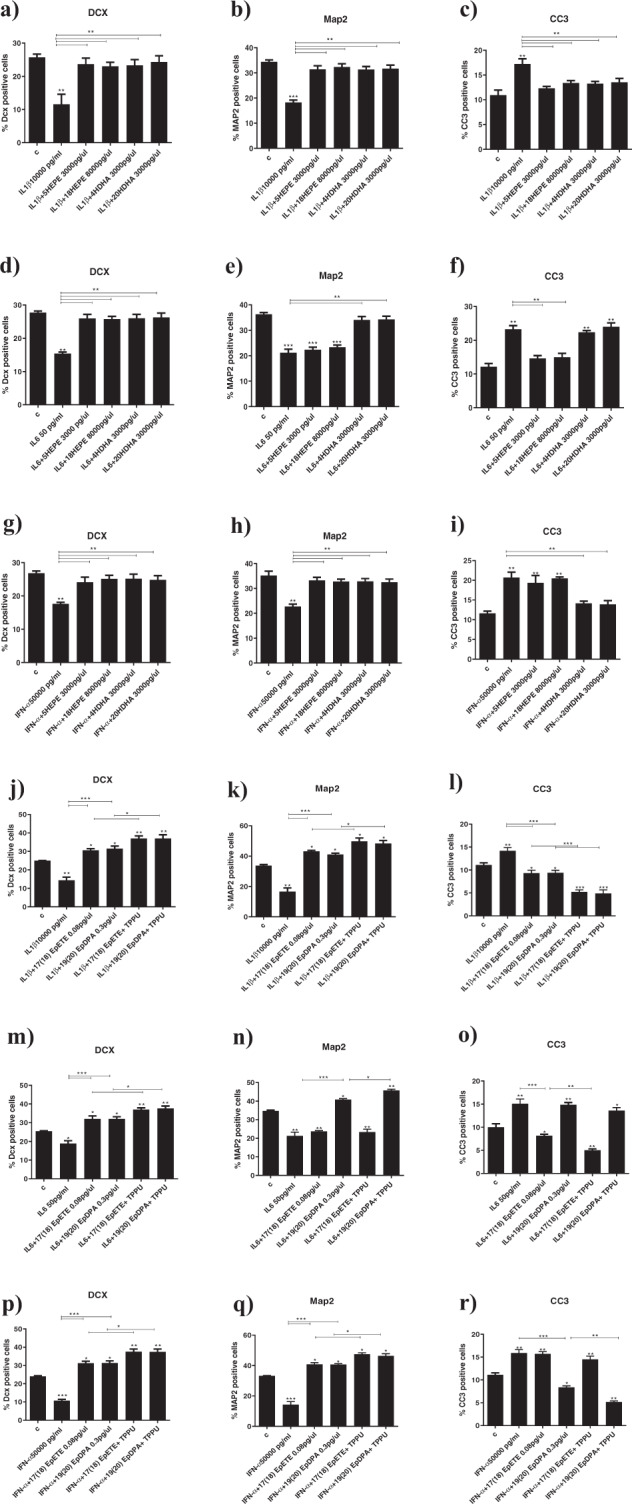
Treatment with higher concentrations of LOX and CYP450 lipid mediators prevents cytokines-induced reduction in neurogenesis and increase in apoptosis. **a**–**i** Co-treatment of cells with higher concentrations of 5-HEPE, 18-HEPE, 4-HDHA or 20-HDHA and IL1β, IL6 or IFN-α partially prevented decrease in DCX+ and Map+cells, and increase in CC3+cells caused by treatment with cytokines alone. **j**–**r** Co-treatment of cells with higher concentrations of 17(18)-EpETE or 19(20)-EpDPA and IL1β, IL6 or IFN-α prevented decrease in DCX+ and Map+cells, and increase in CC3+cells caused by treatment with cytokines alone, and this effect was enhanced by treatment with the sEH inhibitor TPPU. One-way ANOVA with Bonferroni’s post hoc test was performed. Data are shown as mean ± SEM; *p < 0.05, **p < 0.01, ***p < 0.001, compared with vehicle treatment or as indicated.

Results with EPA and DHA metabolites exactly mirrored the effects of EPA and DHA. Specifically, co-treatment of cells with EPA-derived LOX 5-HEPE (1500 pg/µl), or CYP450 *hydroxylase* 18-HEPE (4000 pg/µl), or DHA-derived LOX 4-HDHA (1500 pg/µl) or CYP450 *hydroxylase* 20-HDHA (1500 pg/µl), prevented the decrease in DCX+ and Map+ cells, and the increase in CC3+ cells caused by treatment with the cytokines (see Supplementary Fig. 5a–i and Supplementary Materials). Using the higher concentrations did not change the results (see Fig. 3a–i).

We also found that, similarly to EPA and DHA, co-treatment of cells with either EPA- or DHA-derived CYP450 *epoxygenase* metabolites, 17(18)-EpETE (0.04 pg/µl) and 19(20)-EpDPA (0.15 pg/µl) respectively, prevented the decrease in DCX+ and Map+ cells caused by treatment with cytokines alone, but not the increase in CC3+ cells (see Supplementary Fig. 6a–i). However, interestingly when we used the higher concentrations, we found not only the prevention of the decrease in DCX+ and Map+ cells, but also the prevention of the increase in CC3+cells (see Fig. 3j–r). Moreover, all these effects (of the higher concentrations) were further enhanced by treatment with the sEH inhibitor (TPPU, 1 nM), which prevents the further conversion of 17(18)-EpETE and 19(20)-EpDPA into 17(18)-DiHETE and 19(20)-DiHDPA, respectively (Fig. 3j, k, l). Finally, treatment with the final metabolites, 17(18)-DiHETE (1000 pg/µl) or 19(20)-DiHDPA (0.3 pg/µl), did not affect any of these markers, not even using the higher concentrations (see Supplementary Fig. 6a–i).

### In mechanistic studies, treatment with CYP450 lipid mediators and a sEH inhibitor fully prevents cytokines-induced changes in downstream inflammatory and neurogenic pathways

In the experiments described above, we had shown that EPA-derived LOX 5-HEPE, CYP450 *hydroxylase* 18-HEPE, and CYP450 *epoxygenase* 17(18)-EpETE, and DHA-derived LOX 4-HDHA, CYP450 *hydroxylase* 20-HDHA, and CYP450 *epoxygenase* 19(20)-EpDPA, all prevented the effects of IL1β, IL6 and IFN-α on neurogenesis and apoptosis. We thus wanted to examine the molecular mechanisms through which these metabolites may act. Specifically, we tested whether these metabolites can increase the mRNA expression of the inflammatory transcription factors, STAT1 and NF-kB, and reduce the mRNA expression of the neuroprotective water channel, AQP4, which we had previously identified as relevant to the effects of inflammation on neurogenesis and apoptosis [13, 20, 21, 37]; moreover, we tested whether these metabolites can reduce the levels of these same cytokines measured in the supernatant, as we hypothesised that all these three cytokines were stimulated by treatment with each of them. We thus co-treated differentiated cells with the LOX or CYP450 lipids (at the higher concentrations) and IL1β, IL6 or IFN-α for 1d (for gene expression) and 2d (for cytokines levels in the supernatant) (Fig. 1d, e). For sake of clarity, results are described with reference to the effects of each specific cytokine on these molecular mechanisms.

**Fig. 4:**
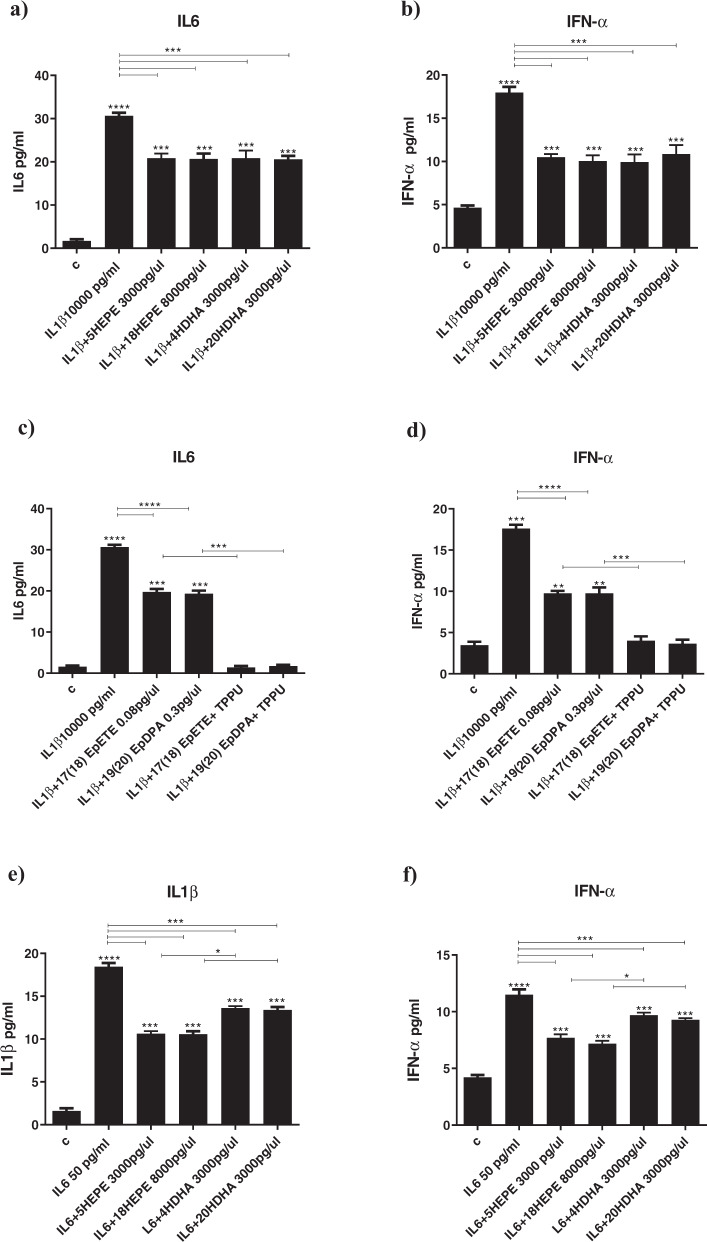

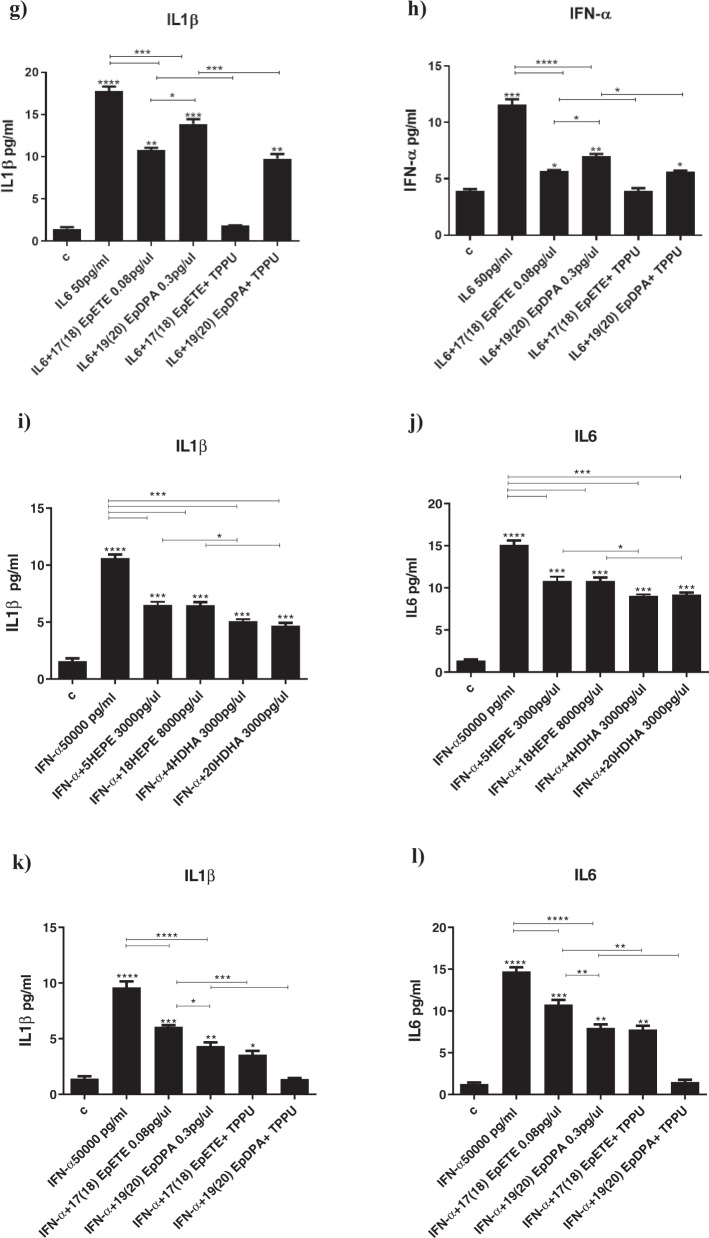
Treatment with CYP450 lipid mediators and sEH inhibitor fully prevents cytokines-induced production of inflammatory cytokines. **a**–**l** Co-treatment with 5-HEPE, 18-HEPE, 4-HDHA, 20-HDHA, 17(18)-EpETE or 19(20)-EpDPA and either IL1β, IL6 or IFN-α prevented the increase in the production of the same cytokines (IL1β, IL6 or IFN-α), induced by treatment with IL1β, IL6 or IFN-α alone. Moreover, co-treatment with 17(18)-EpETE or 19(20)-EpDPA, IL1β, IL6 or IFN-α, and the sHE inhibitor TPPU further enhanced the effect of 17(18)-EpETE and 19(20)-EpDPA. One-way ANOVA with Bonferroni’s post hoc test was performed. Data are shown as mean ± SEM; *p < 0.05, **p < 0.01, ***p < 0.001, ****p < 0.0001, compared with vehicle treatment or as indicated.

IL1β increased STAT1 and NFKB, decreased AQP4 (Supplementary Fig. 7a–c), and increased both IL6 and IFN-α cytokines (Fig. 4a, b). Co-treatment with all the aforementioned metabolites that were able to affect neurogenesis and apoptosis (EPA-derived LOX 5-HEPE (3000 pg/µl), CYP450 *hydroxylase* 18-HEPE (8000 pg/µl), and CYP450 *epoxygenase* 17(18)-EpETE (0.08 pg/µl); or with DHA-derived LOX 4-HDHA (3000 pg/µl), CYP450 *hydroxylase* 20-HDHA (3000 pg/µl), and CYP450 *epoxygenase* 19(20)-EpDPA (0.3 pg/µl), all prevented the increase in STAT1 and NF-kB and the production of IL6 and IFN-α cytokines, but not the reduction in AQP4 gene expression (Supplementary Fig. 7a–f and Fig. 4a–d). Treatment with the sEH inhibitor further enhanced the effects of 17(18)-EpETE and 19(20)-EpDPA on both gene expression and cytokines production (Supplementary Fig. 7d–f and Fig. 4c, d).

IL6 increased STAT1, decreased AQP4, but did not affect NFKB (Supplementary Fig. 7g–i); it also increased both IL1β and IFN-α (Fig. 4e, f). Co-treatment with 5-HEPE, 18-HEPE, 4-HDHA, 20-HDHA, 17(18)-EpETE or 19(20)-EpDPA (as above) did not prevent the increase in STAT1 gene expression, but prevented the decrease in AQP4 expression (Supplementary Fig. 7g–l). The metabolites also prevented the production of IL1β and IFN-α cytokines (Fig. 4e–h). Treatment with the sEH inhibitor further enhanced the effects of 17(18)-EpETE, but not of 19(20)-EpDPA, on AQP4 gene expression, and of both 17(18)-EpETE and 19(20)-EpDPA on cytokines production (Supplementary Fig. 7j–l and Fig. 4g, h).

IFN-α increased STAT1, decreased AQP4, but did not affect NFKB (Supplementary Fig. 7m–o); it also increased both IL1β and IFN-α (Fig. 4i, j). Co-treatment with 5-HEPE, 18-HEPE, 4-HDHA or 20-HDHA (as above) prevented the increase in STAT1 and the decrease in AQP4 gene expression (Supplementary Fig. 7m–o), and the production of IL1β and IL6 cytokines (Fig. 4i, j); co-treatment with 17(18)-EpETE or 19(20)-EpDPA prevented the increase in STAT1 and the production of IL1β and IL6, but *not* the decrease in AQP4. Treatment with the sEH inhibitor further enhanced the effects of 19(20)-EpDPA on STAT1 gene expression (Supplementary Fig. 7p–r and Fig. 4k, l). Moreover, treatment with the sEH inhibitor and either 17(18)-EpETE or 19(20)-EpDPA *also* prevented decrease in AQP4 expression (Supplementary Fig. 7p–r).

### Increased levels of LOX and CYP450 lipid mediators in depressed patients receiving ω-3 PUFAs intervention correlates with a reduction in the depressive symptomatology

**Fig. 5:**
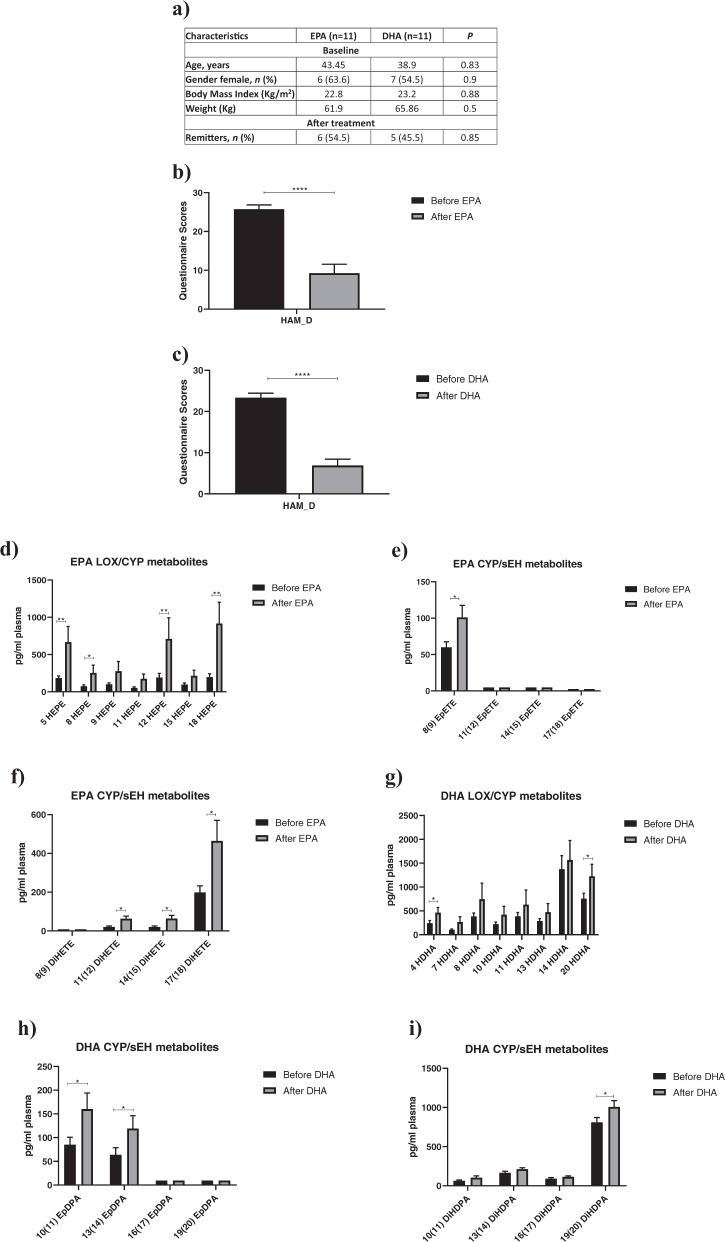
Increased levels of LOX and CYP450 lipid mediators in depressed patients receiving ω-3 PUFAs intervention. **a** Demographic and clinical characteristics of the depressed patients by treatment groups. Chi-square *χ*^2^ test (for categorical variables) or Mann Whitney *U* (for continuous values). Continuous data are shown as mean, categorical data are presented as n (%). **b**, **c** Differences in HAM-D scores before and after treatment with either EPA or DHA. **d**–**f** 5-, 8-, 9-, 11-, 12-, 15-, 18-HEPE and 8(9)-, 11(12)-, 14(15)-, 17(18)-EpETE, -DiHETE metabolites levels before and after treatment with either EPA. **g**–**i** 4-, 7-, 8-, 10-, 11-, 13-, 14-, 20-HDHA, 10(11)-, 13(14)-, 16(17)-, 19(20)-EpDPA, -DiHDPA metabolites levels before and after treatment with either DHA. Wilcoxon’s t test, with Bonferroni’s post hoc test was performed. Data are shown as mean ± SEM; *p < 0.05, **p < 0.01, ****p < 0.0001, compared as indicated. Lower limit of detection was reported for 11(12)-, 14(15), 17(18)-EpETE, 8(9)-DiHETE, 16(17), 19(20)-EpDPA.

Finally, in order to validate our in vitro findings, we measured the same panel of EPA- and DHA-derived lipid metabolites also in plasma samples of depressed patients before and after 12 weeks intervention with either EPA (3.0 g/day; *n* = 11) or DHA (1.4 g/d; *n* = 11). The patients’ baseline demographic and psychiatric characteristics are shown in Fig. 5a. Briefly, there was a significant reduction in the HAM-D scores after treatment with either EPA (average reduction = from 25.7 to 9.3; Fig. 5b) or DHA (average reduction = from 23.4 to 6.9; Fig. 5c), with around half of the sample showing remission, defined as HAM-D scores ≤ 7, after treatment (54.5% for EPA sample, 45.5% for DHA sample; Fig. 5a).

In patients receiving treatment with EPA, there was a significant increase in the same EPA-derived (but not the DHA-derived) LOX and CYP450 lipid metabolites (both *hydroxylase* and *epoxygenase*) previously identified in our in vitro experiments (+73% 5-HEPE, +72% 8-HEPE, +73% 12-HEPE, +79% 18-HEPE, +42% 8(9)-EpETE, +68% 11(12)-DiHETE, +85% 14(15)-DiHETE, +57% 17(18)-DiHETE; see Fig. 5d, e, f). Similarly, in patients receiving treatment with DHA, there was a significant increase in the same DHA-derived (but not the EPA-derived) LOX and CYP450 lipid metabolites (both *hydroxylase* and *epoxygenase*) previously identified in our in vitro experiments (+47% 4-HDHA, +38% 20-HDHA, +46% 10(11)-EpDPA, +47% 13(14)-EpDPA, +19% 19(20)-DiHDPA; see Fig. 5g, h, i). There were no significant differences in any other metabolite analysed when comparing before and after EPA or DHA treatment (see Supplementary Fig. 8a–r). In exploratory correlation analyses, we found that, across all samples, higher levels of some metabolites were correlated with lower levels of depressive symptoms, for example, EPA-derived 5-, 12-, 18-HEPE, 8(9)-EpETE, and 11(12)-, 14(15)-, 17(18)-DiHETE (*r*_s_ = −0.5, *p*=0.02; *r*_s_ = −0.4, *p* = 0.05; *r*_s_ = −0.5, *p*=0.02; *r*_s_ = −0.4, *p* = 0.05; *r*_s_ = −0.5, *p* = 0.02; *r*_s_ = −0.4, *p* = 0.03; *r*_s_ = −0.5, *p* = 0.02, respectively; see Supplementary Fig. 9a–h), and DHA-derived 10(11)-, 13(14)- EpDPA and 19(20)-DiHDPA (r_s_ = −0.3, *p* = 0.05; *r*_s_ = −0.3, *p*=0.05; *r*_s_ = −0.5, *p* = 0.02, respectively; see Supplementary Fig. 9i–m).

## Discussion

In this study, we demonstrate, for the first time, that EPA and DHA are metabolised in neurones into LOX (5-HEPE, 4-HDHA), CYP450 *hydroxylase* (18-HEPE, 20-HDHA) and *epoxygenase* (17(18)-EpETE and 19(20)-EpDPA) lipid mediators that have the ability to prevent the reduction in neurogenesis and the increase in neuronal apoptosis and in inflammatory transcription factors induced by pro-inflammatory cytokines. Together with our new evidence that these same metabolites raise in the plasma of depressed patients receiving treatment with either EPA or DHA, our paper points at these lipid mediators as a novel molecular mechanism underpinning the antidepressant, anti-inflammatory and neuroprotective effects of PUFAs.

Firstly, we were able to identify differential effects of EPA and DHA in preventing cytokines-induced decrease in neurogenesis and increase in apoptosis, also in line with previous evidence [13, 42–45]. Specifically, EPA prevents the reduction in neurogenesis (Map2+ cells) only in the presence of IL1β and IFN-α, while DHA acts on neurogenesis with all the three cytokines; moreover EPA prevents the increase in apoptosis (CC3+ cells) induced by IL1β and IL6, while DHA acts on apoptosis in the presence of IL1β and IFN-α. This is consistent with the evidence that EPA acts more as an anti-inflammatory agent, able to inhibit pathways related to the innate immune response [13, 46], and thus exerts its anti-apoptotic properties. This is in line with previous evidence showing the ability for EPA to decrease IL6 production upon cell stimulation with lipopolysaccharide, and to prevent cell apoptosis [47]. In contrast, DHA has a more neuroprotective and neurogenic role [48], and this is independent of the type of cytokines to which it is exposed to. Interestingly, we have previously shown that treatment with DHA can prevent reduction in neurogenesis caused by IL1β, much like treatment with antidepressants, sertraline and venlafaxine, does [13]. Evidence from the presence study seems to confirm similar effects also when in presence of IL6 and IFN-α.

Interestingly, co-treatment with LOX (5-HEPE, 4-HDHA), CYP450 *hydroxylase* (18-HEPE, 20-HDHA) or *epoxygenase* (17(18)-EpETE and 19(20)-EpDPA) metabolites and IL1β, IL6 or IFN-α all prevent cytokines-induced reduction in neurogenesis and increase in apoptosis, with exactly the same effects of their corresponding precursor, EPA or DHA. This finding demonstrates for the first time the ability for these metabolites to exert neurogenic and antiapoptotic properties in the context of human hippocampal neurogenesis, a mechanism relevant to depression [17–19]. Indeed, so far, LOX and CYP450 lipids have only been studied for their ability to reduce inflammation and oxidative stress, or for regulating tissue homoeostasis [49, 50], with no evidence yet that they could be produced in neurones or that they could modulate neurogenesis or neuroplasticity. Interestingly, these metabolites are produced in the presence of EPA or DHA upon cell stimulation with either IL1β, IL6 or IFN-α, but not during pre-treatment with EPA or DHA alone. These findings suggest the ability for EPA and DHA to remain stored within the cell membrane for longer periods until a later insult, for example with cytokines, can activate the enzymes (LOX and CYP450) responsible for EPA and DHA synthesis into their final metabolic products, which are responsible to reduce inflammation and restore cell homoeostasis [51]. Indeed, ω-3 PUFAs are usually kept stored in the cell membrane and serve as fundamental structural components of membrane complexes [52]. In particular, ω-3 PUFAs can change the expression of surface receptors and ultimately regulate membrane-based cellular responses, especially when in presence of an inflammatory or stressful stimulus [53].

Interestingly, the same LOX and CYP450 *hydroxylase* and *epoxygenase* metabolites that we have identified in vitro – and only these metabolites – are also increased in plasma samples of depressed patients receiving nutritional intervention with either EPA or DHA. To our knowledge this is the first study to measure ω-3 PUFAs metabolites in a clinical sample of patients with major depression exposed to treatment with either EPA or DHA (and which also showed a parallel decrease in depressive symptoms). Only one observational study reported the analysis of similar LOX- and CYP450-derived lipid classes and found, as in our study, negative correlations between metabolites levels and depressive symptoms; however the cohort consisted of patients with seasonal depression, and only few lipid species were identified, produced naturally rather than during PUFAs treatment [28]. Nevertheless, it is interesting to notice that the magnitude of the effects of PUFAs administration in our study (Cohen’s *d* = 0.73-0.75) is of the same magnitude of the difference detected in this study [28] when comparing the levels of ω-3 PUFAs metabolites in the same patients when they have low versus high levels of depression, thus supporting the notion that the changes that we measure in PUFAs metabolites following PUFAs administration are clinically relevant. From a clinical point of view, it is also important to notice the one study which measured LOX- and CYP450-derived EPA/DHA lipid metabolites concentrations in plasma of healthy individuals. The study reported much higher concentrations of these metabolites, when compared with concentrations observed in our cohort of depressed patients at baseline [54]. Although a direct comparison is of course limited by the methodological differences between the studies, this evidence suggests an impaired endogenous production of these anti-inflammatory and neuroprotective metabolites in patients with depression.

Our study also shows that co-treatment with the CYP450 *epoxigenase*-derived 17(18)-EpETE and 19(20)-EpDPA, and an inhibitor of the sEH enzyme, significantly enhances the neurogenic and anti-apoptotic effect of 17(18)-EpETE and 19(20)-EpDPA. The sEH enzyme plays an important role in ω-3 PUFAs metabolism, as it allows the conversion of EpETEs and EpDPAs into their less active dihydroxy derivatives DiHETEs and DiHDPAs [50] (see Fig. 1a). Since we have shown that both 17(18)-EpETE and 19(20)-EpDPA have themselves neurogenic and anti-apoptotic properties, our findings confirm that maximising their bioavailability by blocking their further metabolism enhances these biological actions. Interestingly, previous evidence has demonstrated that oral administration of TPPU, the same sEH inhibitor used in our study, can exert rapid antidepressant effects in an inflammation model of depression in mice [55]. Also, mice showing depressive-like behaviours have higher sEH protein levels in the brain, and this has been confirmed in post-mortem brain tissues of patients with major depressive disorder [55]. Taken together with our study, these lines of evidence suggest a possible role for sEH as therapeutic targets for treatment of depression [56]. While sEH inhibitors like TPPU or AUDA, have been previously used both in in vitro and in vivo models of inflammation [56], a new drug, GSK2256294A, able to selectively inhibit the sEH enzyme, has been recently tested and validated for its safety and tolerability in a clinical cohort of obese smokers with pulmonary inflammation [57]. Due to its low molecular weight, GSK2256294A has also been used in patients developing neuroinflammation after subarachnoid haemorrhage (ClinicalTrials.gov identifier NCT03318783), therefore making GSK2256294A a valid option for drug repurposing also in the context of other inflammation-associated brain disorders, including depression, where at least a sub-group of patients often presents chronic levels of peripheral and central inflammation [1, 2, 14].

In terms of mechanisms, we also found that treatment with LOX (5-HEPE, 4-HDHA), CYP450 *hydroxylase* (18-HEPE, 20-HDHA) or *epoxygenase* metabolites (17(18)-EpETE and 19(20)-EpDPA) and IL1β, IL6 or IFN-α, prevented cytokines-induced increase in STAT1, NF-kB gene expression and production of these same cytokines, as well as reduction in AQP4 gene expression. STAT1 and NF-kB are pro-inflammatory transcription factors, whereas AQP4 is a neuroprotective water channel protein, all of which had previously identified as relevant to the effects of inflammation on neurogenesis and apoptosis [13, 20, 21, 37]. Of particular interest is the fact that, in our study, treatment with 17(18)-EpETE or 19(20)-EpDPA and the sEH inhibitor, but not with 17(18)-EpETE or 19(20)-EpDPA alone, prevented the decrease in AQP4 caused by IFN-α, and that this effect was much stronger for 19(20)-EpDPA than for 17(18)-EpETE. This is in line with our other findings showing the ability for 19(20)-EpDPA, but not for 17(18)-EpETE, both with the inhibitor, to prevent IFN-α-induced increase in apoptosis, while both equally prevented reduction in neurogenesis. Indeed, AQP4 is particularly important for the suppression of apoptosis [20], and, in the context of IFN-α, this protein can be considered a mechanistic target for the anti-apoptotic effect of 19(20)-EpDPA, but only when in presence of the sEH inhibitor.

The main limitation of our study is that the experimental findings are generated from an in vitro system, with an immortalised cell line. However, all our previous results with this model have been replicated in animal and clinical studies, including changes in neurogenesis by cytokines, cortisol, and antidepressants, and changes in stress- and inflammation- regulated genes both in the whole blood mRNA of depressed patients and in the hippocampal mRNA of animal models of depression [13, 20, 29, 30, 34–36, 46]. We also acknowledge the relatively small sample size of depressed patients used to measure ω-3 PUFAs-derived lipid mediators and the fact that the study was not a randomised placebo control trial; in future studies, it would be important to replicate these findings in a much larger cohort of depressed patients, and to extend our investigations also to other classes of ω-3 PUFAs metabolites, including endocannabinoids [58]. Finally, it is important to highlight the fact that, concentrations of EPA and DHA (both 10 µM), used in this study and in our previous in vitro studies [13, 37, 41], are concentrations that likely cannot be achieved with consumption of food rich in ω-3 PUFAs [59], but rather require therapeutic PUFAs supplements. Indeed, these in vitro concentrations resemble those found in the brain of individuals receiving therapeutic doses of ω-3 similar to those administered in our clinical study [60]. Therefore, we can conclude that the in vitro concentrations and the clinical doses of ω-3 PUFAs are relatively equivalent, especially with respect to EPA and DHA production in the brain; moreover, similar oral doses have also been used in several other clinical studies [7, 39, 60–62].

In summary, our study confirms and extends previous evidence for the antidepressant, anti-inflammatory and neuroprotective abilities of EPA and DHA, and identifies LOX-derived 5-HEPE and 4-HDHA, and CYP450-derived 18-HEPE, 20-HDHA, 17(18)-EpETE and 19(20)-EpDPA as mediators of these effect of ω-3 PUFAs. Further corroboration that these metabolites increase in the plasma of depressed patients that receive EPA or DHA (with a parallel improvement in depressive symptoms) supports the relevance of LOX and CYP450 hydroxy and epoxy PUFA derivatives, and the enzymes involved in their metabolism, as potential therapeutic strategy for patients suffering from depressive symptoms, at least in the subgroup of patients with increased inflammation.

## Supplementary information

Supplementary Materials

Supplementary Figure 1

Supplementary Figure 2

Supplementary Figure 3

Supplementary Figure 4

Supplementary Figure 5

Supplementary Figure 6

Supplementary Figure 7

Supplementary Figure 8

Supplementary Figure 9
