# Supplementary material for: Omega-3 polyunsaturated fatty acids protect against inflammation through production of LOX and CYP450 lipid mediators: relevance for major depression and for human hippocampal neurogenesis
Source: Mol Psychiatry. 2021 Jun 16;26(11):6773–88. doi: 10.1038/s41380-021-01160-8 (PMC8760043; doi:10.1038/s41380-021-01160-8)
Supplement: Supplementary file 1 — Supplementary Materials [file 41380_2021_1160_MOESM1_ESM.pdf]

For submission to Molecular Psychiatry

Omega-3 polyunsaturated fatty acids protect against inflammation through production of LOX and CYP450 lipid mediators: relevance for major depression and for human hippocampal neurogenesis

Supplementary Materials

Alessandra Borsini, PhD[1*], Anna Nicolaou, PhD[2,3], Dolores Camacho-Muñoz, PhD[2], Alexandra C. Kendall, PhD[2], Maria Grazia Di Benedetto, MSc[1,4], Juliette Giacobbe, MSc[1], Kuan-Pin Su, PhD, MD[1,5,6*], Carmine M. Pariante, PhD, MD[1]

[1] Stress, Psychiatry and Immunology Laboratory, Institute of Psychiatry, Psychology and Neuroscience, Department of Psychological Medicine, King's College London, UK

[2] Laboratory for Lipidomics and Lipid Biology, Division of Pharmacy and Optometry, School of Health Sciences, Faculty of Biology, Medicine and Health, The University of Manchester, UK

[3] Lydia Becker Institute of Immunology and Inflammation, Faculty of Biology, Medicine and Health, The University of Manchester, UK

[4] Biological Psychiatry Unit, IRCCS Istituto Centro San Giovanni di Dio, Fatebenefratelli, Brescia, Italy

[5] College of Medicine, China Medical University, Taichung, Taiwan

[6] Depression Center, An-Nan Hospital, China Medical University, Tainan, Taiwan

* Corresponding Authors
Alessandra Borsini, PhD
Stress, Psychiatry and Immunology Lab & Perinatal Psychiatry
Institute of Psychiatry, Psychology and Neuroscience, King's College London
G.32.01, The Maurice Wohl Clinical Neuroscience Institute
Cutcombe Road, London, SE5 9RT
Tel: 020 7848 0726
Email: alessandra.borsini@kcl.ac.uk

Kuan-Pin Su, MD, PhD
An-Nan Hospital, China Medical University
Tainan, Taiwan
Email: cobolsu@gmail.com

SUPPLEMENTARY METHODS

In vitro treatment with ω-3 PUFAs, lipid mediators and cytokines: Concentrations of EPA and DHA (non-esterified), IL1β, IL6 and IFN-α were previously validated and used in all our studies with respectively, ω-3 PUFA[1-4] and/or cytokines[1, 2, 5-7]. EPA, DHA, LOX and CYP450 lipid mediators, LOX, CYP450 and sEH inhibitor were all dissolved in 100% EtOH. Our cells express COX-2, 5-, 12-, 15-LOX, CYP2C8, CYP2C9 and CYP2J2 enzymes (data not shown).

Immunocytochemistry and quantification of immunofluorescence: Immature and mature neurons were assessed using respectively DCX and Map2 (primary antibody: rabbit anti-DCX, 1:500; mouse anti-Map2, 1:500, Abcam, Cambridge, UK; secondary antibody: Alexa 488 donkey anti-rabbit; 1:1000, Invitrogen; Alexa 555 donkey anti-mouse, 1:1000, Invitrogen), whereas apoptotic cells were examined using CC3 (primary antibody: rabbit anti-CC3, 1:500, Technol; secondary antibody: Alexa 555 donkey anti-rabbit, 1:1000, Invitrogen). All cells were labelled using DAPI dye (Figure 1b, c, d and e). The number of DCX and Map2 positive cells over total DAPI positive cells was counted using an insight automated imaging platform (CellInsight). Detailed information on the staining and imaging analyses procedure can be found in our previous publications[2, 5, 6]. At least six independent experiments were conducted on independent biological cultures, and each sample was tested in quadruplicate.

Analysis of lipid mediators in cell culture supernatants and human plasma: Lipid mediators were extracted from cell supernatant and human plasma samples using ice-cold methanol (15%, v/v), and 12-hydroxyeicosatetraenoic acid (HETE)-d8, prostaglandin (PG) B2-d4, 8 (9)-epoxy eicosatrienoic acid-d11, and 8,9-dihydroxyeicosatrienoic acid-d11 (20 ng each/sample; CaymanChemicals, Ann Arbor, MI, USA) were used as internal standards. The extracts were semipurified using solid-phase extraction cartridges (C18-E; Phenomenex,

Macclesfield, United Kingdom) to remove interferences, such as proteins and preconcentrate the analytes, and then quantitated using compound-specific calibration lines constructed using commercially-available standards (Cayman Chemical, Ann Arbor, Michigan). UPLC/ESI-MS/MS analysis was performed on an Acquity UPLC pump (Waters, Wilmslow, United Kingdom) coupled to an electrospray ionization triple quadrupole mass spectrometer (Xevo TQ-S; Waters) as previously described[8]. See Supplementary Figure 2 for the full panel of metabolites analysed. At least six independent experiments were conducted on independent biological cultures, and each sample was tested in duplicates.

RNA isolation and quantitative real-time PCR (qPCR) analysis: RNA was isolated using the RNeasy Micro Kit (Qiagen), RNA quantity and quality were assessed by evaluation of the A260/280 and A260/230 ratios using a Nanodrop spectrometer (NanoDrop Technologies). SuperScript® III Reverse Transcriptase (Life Technologies) was then used to synthesise cDNA. Both target (STAT1, NF-kB and AQP4) and housekeeping genes (RPL13A and BACT) expression levels were then analyzed by TaqMan qRT-PCR instrument (CFX384 real time system, Bio-Rad, California, USA) using the iScriptTM two-step RTPCR kit for probes (Bio-Rad, California, USA) (Figure 1d, e). Detailed information on the gene expression analyses procedure can be found in our previous publications[9, 10]. At least six independent experiments were conducted on independent biological cultures, and each sample was tested in triplicate.

Clinical samples: Patients' sociodemographic characteristics (age, gender) were recorded at the initial assessment together with body weight and the body mass index (BMI), which was calculated as the participant's weight (kg) divided by the square of standing height (m) and measured[11]. The severity of depressive symptoms was measured by trained psychiatrists using the 21-item HAMD[11] before therapy (baseline), and at week 12. Based on the changes in

HAMD scores at each visit, clinical remission was defined as the HAMD scores ≤7[12]. The exclusion criteria for patients were: (1) a recent or past history of other DSM-IV axis-I diagnoses besides unipolar major depression, including psychotic disorders, organic mental disorders, impulse control disorders, substance use disorder or substance abuse (last 6 months prior to the study), and bipolar disorders; (2) axis-II diagnoses, including borderline and antisocial personality disorder; (3) a notable medical comorbidity; (4) acutely suicidal ideation and attempt were noted that close monitoring such as hospitalization was necessary; (5) all kinds of substance users including tobacco smokers and alcohol users, such as current or ex-smokers and current or ex-drinkers; and (6) regular consumption of ω-3 PUFAs supplements or a habit of eating fish equal to or > 4 times per week.

SUPPLEMENTARY RESULTS

Treatment with LOX or CYP450 inhibitor, but not COX-2 inhibitor, prevents the effect of EPA and DHA against cytokines-induced reduction in neurogenesis and increase in apoptosis

We pre-treated cells with either EPA or DHA (both 10μM) with or without a selective inhibitor of COX-2 enzymes (CAS 416901-58-1; 1, 4 and 8μM), LOX (NDGA; 1, 10 and 30μM) or CYP450 (SKF525A; 1μM) for 2 days during differentiation, followed by treatment with either IL1β (10000pg/ml), IL6 (50pg/ml) or IFN-α (50000pg/ml), again with or without LOX and CYP450 inhibitors, for additional 2d (Figure 1c).

Treatment with COX-2 inhibitor (1μM) did not cause any changes in results (Supplementary Figure 3a-i), even with higher concentrations (4 and 8μM; data not shown). However, pre-treatment with EPA or DHA (without inhibitors), followed by co-treatment with LOX inhibitor and IL1β prevented the effect of EPA or DHA against cytokine-induced

reduction in DCX+ and Map2+cells, and increase in CC3+cells (Supplementary Figure 4a, b and c). Also, pre-treatment with EPA or DHA (without inhibitors), followed by co-treatment with LOX inhibitor and IL6 prevented the effect of EPA or DHA against cytokine-induced reduction in DCX+ and Map2+cells, and increase in CC3+cells (Supplementary Figure 4d, e and f). Similarly, pre-treatment with EPA or DHA (without inhibitors), followed by co-treatment with LOX inhibitor and IFN-α prevented the effect of EPA or DHA against cytokine-induced reduction in DCX+ and Map2+cells, and increase in CC3+cells (Supplementary Figure 4g, h and i). Of note, this preventive effect was confirmed when LOX inhibitor was used both during pre-treatment with EPA or DHA and in co-treatment with cytokines (IL1β, IL6 or IFN-α) (Supplementary Figure 4a-i). Although, higher concentrations of LOX inhibitor (NDGA; 10 and 30μM) have also been tested, results remained unchanged (data not shown).

In contrast, pre-treatment with EPA or DHA (without inhibitors), followed by co-treatment with CYP450 inhibitor and IL1β prevented the effect of EPA or DHA against cytokine-induced reduction in DCX+ and Map2+cells, and increase in CC3+cells (Supplementary Figure 4j, k and l). Similarly, pre-treatment with EPA or DHA (without inhibitors), followed by co-treatment with CYP450 inhibitor and IL6 prevented the effect of EPA or DHA against cytokine-induced reduction in DCX+ and Map2+cells, and increase in CC3+cells (Supplementary Figure 4m, n and o). Finally, pre-treatment with EPA or DHA (without inhibitors), followed by co-treatment with CYP450 inhibitor and IFN-α prevented the effect of EPA or DHA against cytokine-induced reduction in DCX+ and Map2+cells, and increase in CC3+cells (Supplementary Figure 4p, q and r). Of note, this preventive effect was confirmed when CYP450 inhibitor was used both during pre-treatment with EPA or DHA and in co-treatment with cytokines (IL1β, IL6 or IFN-α) (Supplementary Figure 4a-i).

Treatment with lower concentrations of LOX or CYP450 hydroxy lipid mediators prevents cytokines-induced reduction in neurogenesis and increase in apoptosis

We exposed cells to co-treatment with LOX or CYP450 *hydroxylase* lipids and either IL1β, IL6 or IFN-α (as above) for 2d (Figure 1d, e). Concentrations of lipid mediators were selected based on the levels of metabolites previously detected in cell supernatant (Figure 2j).

Co-treatment of cells with EPA-derived LOX 5-HEPE (1500pg/µl), or CYP450 *hydroxylase* 18-HEPE (4000pg/µl), or DHA-derived LOX 4-HDHA (1500pg/µl) or CYP450 *hydroxylase* 20-HDHA (1500pg/µl) and IL1β prevented decrease in DCX+cells caused by treatment with the cytokine alone (Supplementary Figure 5a). Similar findings were identified for Map2+cells (Supplementary Figure 5b) and CC3+cells (Supplementary Figure 5c).

Co-treatment of cells with 5-HEPE, 18-HEPE, 4-HDHA or 20-HDHA (as above) and IL6 prevented decrease in DCX+cells caused by treatment with the cytokine alone (Supplementary Figure 5d). However, similar to EPA and DHA, only 4-, 20-HDHA, but not 5-, 18-HEPE prevented a reduction in Map2+cells caused by the cytokine (Supplementary Figure 5e), whereas 5-, 18-HEPE, but not 4-, 20-HDHA prevented increase in CC3+cells caused by treatment with IL6 alone (Supplementary Figure 5f).

Also, co-treatment of cells with 5-HEPE, 18-HEPE, 4-HDHA or 20-HDHA (as above) and IFN-α prevented decrease in DCX+cells caused by treatment with the cytokine alone (Supplementary Figure 5g). Similar findings were identified for Map2+cells (Supplementary Figure 5h). Whereas, similar to EPA and DHA, 4-, 20-HDHA, but not 5-, 18-HEPE prevented increase in CC3+cells caused by treatment with IFN-α alone (Supplementary Figure 5i).

Treatment with lower concentrations of CYP450 epoxy lipid mediators prevent cytokines-induced reduction in neurogenesis, but not increase in apoptosis

We exposed cells to co-treatment with CYP450 *epoxygenase* lipids and either IL1β, IL6 or IFN-α (as above) for 2d (Figure 1d, e).

Co-treatment with either EPA-derived 17(18)-EpETE (0.04pg/μl) or DHA-derived 19(20)-EpDPA (0.15pg/μl) and IL1β prevented reduction in DCX+ cells caused by treatment with the cytokine alone, as previously seen for EPA and DHA (Supplementary Figure 6a). Similar findings were observed for Map2+cells (Supplementary Figure 6b). These effects were further enhanced when in presence of the sHE inhibitor TPPU (1nM) (Supplementary Figure 6a, b). However, in contrast with EPA and DHA, neither of these metabolites prevented increase in CC3+cells (Supplementary Figure 6c).

Also, co-treatment with either 17(18)-EpETE or 19(20)-EpDPA (as above) with IL6 prevented reduction in DCX+ cells caused by treatment with the cytokine alone (Supplementary Figure 6d), however similar to EPA and DHA, only EpDPA but not EpETE prevented a reduction in Map2+cells caused by the cytokine (Supplementary Figure 6e). These effects were further enhanced when in presence of the sHE inhibitor TPPU (1nM) (Supplementary Figure 6d, e). Whereas, in contrast with EPA and DHA, neither of these metabolites prevented increase in CC3+cells (Supplementary Figure 6f).

Finally, as for EPA and DHA, co-treatment with either 17(18)-EpETE or 19(20)-EpDPA (as above) with IFN-α prevented reduction in DCX+ cells caused by treatment with the cytokine alone (Supplementary Figure 6g). Similar findings were observed for Map2+cells (Supplementary Figure 6h). These effects were further enhanced when in presence of the sHE

inhibitor TPPU (1nM) (Supplementary Figure 6g, h). However, in contrast with EPA and DHA, neither of these metabolites prevented increase in CC3+cells (Supplementary Figure 6i).

References:

1. Horowitz MA, Wertz J, Zhu D, Cattaneo A, Musaelyan K, Nikkheslat N *et al.* Antidepressant compounds can be both pro- and anti-inflammatory in human hippocampal cells. *Int J Neuropsychopharmacol* 2014; **18**(3).

2. Borsini A, Alboni S, Horowitz MA, Tojo LM, Cannazza G, Su KP *et al.* Rescue of IL-1beta-induced reduction of human neurogenesis by omega-3 fatty acids and antidepressants. *Brain, behavior, and immunity* 2017; **65:** 230-238.

3. Borsini A, Stangl D, Jeffries AR, Pariante CM, Thuret S. The role of omega-3 fatty acids in preventing glucocorticoid-induced reduction in human hippocampal neurogenesis and increase in apoptosis. *Translational psychiatry* 2020; **10**(1)**:** 219.

4. Alboni S, Gibellini L, Montanari C, Benatti C, Benatti S, Tascedda F *et al.* N-acetyl-cysteine prevents toxic oxidative effects induced by IFN-alpha in human neurons. *Int J Neuropsychopharmacol* 2013; **16**(8)**:** 1849-1865.

5. Borsini A, Cattaneo A, Malpighi C, Thuret S, Harrison NA, Zunszain PA *et al.* Interferon-Alpha Reduces Human Hippocampal Neurogenesis and Increases Apoptosis via Activation of Distinct STAT1-Dependent Mechanisms. *Int J Neuropsychoph* 2018; **21**(2)**:** 187-200.

6. Borsini A, Di Benedetto MG, Giacobbe J, Pariante CM. Pro- and anti-inflammatory properties of interleukin (IL6) in vitro: relevance for major depression and for human hippocampal neurogenesis. *Int J Neuropsychopharmacol* 2020.

7. Zunszain PA, Anacker C, Cattaneo A, Choudhury S, Musaelyan K, Myint AM *et al.* Interleukin-1beta: a new regulator of the kynurenine pathway affecting human hippocampal neurogenesis. *Neuropsychopharmacology : official publication of the American College of Neuropsychopharmacology* 2012; **37**(4)**:** 939-949.

8. Kendall AC, Pilkington SM, Murphy SA, Del Carratore F, Sunarwidhi AL, Kiezel-Tsugunova M *et al.* Dynamics of the human skin mediator lipidome in response to dietary omega-3 fatty acid supplementation. *FASEB journal : official publication of the Federation of American Societies for Experimental Biology* 2019; **33**(11)**:** 13014-13027.

9. Borsini A, Cattaneo A, Malpighi C, Thuret S, Harrison NA, Consortium MRCI *et al.* Interferon-Alpha Reduces Human Hippocampal Neurogenesis and Increases Apoptosis via Activation of Distinct STAT1-Dependent Mechanisms. *Int J Neuropsychopharmacol* 2018; **21**(2)**:** 187-200.

10. Anacker C, Cattaneo A, Luoni A, Musaelyan K, Zunszain PA, Milanesi E *et al.* Glucocorticoid-related molecular signaling pathways regulating hippocampal neurogenesis. *Neuropsychopharmacology : official publication of the American College of Neuropsychopharmacology* 2013; **38**(5)**:** 872-883.

11. Yang B, Lin L, Bazinet RP, Chien YC, Chang JP, Satyanarayanan SK *et al.* Clinical Efficacy and Biological Regulations of omega-3 PUFA-Derived Endocannabinoids in Major Depressive Disorder. *Psychother Psychosom* 2019; **88**(4)**:** 215-224.

12. Hamilton M. A rating scale for depression. *J Neurol Neurosurg Psychiatry* 1960; **23:** 56-62.

Figures Caption

Supplementary Figure 1. Representative immunostaining images of neurogenic and apoptotic markers in control condition. Cells were treated with media containing EGF, bFGF, 4-OHT for 1 day during proliferation, followed by 4 days during differentiation with media without growth factors. Neuroblasts were detected by DCX (green) over the total number of cells DAPI (blue) (a), mature neurons were detected by Map2 (red) over the total number of cells DAPI (blue) (b), whereas apoptotic cells were stained by CC3 (red) over the total number of cells DAPI (blue) (c). Scalebar: 10µm.

Supplementary Figure 2. List of lipid mediators analysed in cell supernatant and plasma, and example of ion chromatograms. (a) List of lipid mediators analysed in cell supernatant of cells pre-treated with EPA or DHA for 48h during differentiation, followed by treatment with cytokines (IL1β, IL6 or IFN-α) for 6h, and in plasma samples of depressed patients before and after treatment with either EPA or DHA. **(b-i)** Example of reconstructed ion chromatograms with authentic lipid standard (STD) showing production of selected lipids by cells pre-treated with EPA or DHA for 48h during differentiation, followed by treatment with IL1β for 6h. Legend: LA, linoleic acid; AA, arachidonic acid; ALA, alpha-linolenic acid; OxoODE, oxoode; HODE, hydroxyoctadecadienoic acid; Trans EKODE, epoxy-keto-octadecenoic acid; EpOME, epoxyoctadecenoic acid; DiHOME, dihydroxyoctadecenoic acid; oxo-ETE, oxo-eicosatetraenoic acid; oxo-EPE, oxo-eicosapentaenoic acid; HETE, hydroxyeicosatetraenoic acid; EET, epoxyeicosatrienoic acid; HETrE, hydroxyeicosatrienoic acid; DHET, dihydroxyeicosatrienoic acid; LTB, leukotriene; LXA, lipoxin; PG, prostaglandin; TXB, thromboxane; HOTrE, octadecatrienoic acid; HEPE, hydroxyeicosapentaenoic acid; EpETE, epoxyeicosatetraenoic acid DiHETE, dihydroxyeicosatetraenoic acid; DiHEPE, dihydroxyicosapentaenoic acid; Rv, resolving; HDHA, hydroxydocosahexaenoic acid, EpDPA, epoxydocosapentaenoic acid; DiHDPA, dihydroxydocosapentaenoic acid.

Supplementary Figure 3. Co-treatment with COX-2 inhibitor and cytokines did not prevent the effect of EPA and DHA against cytokines-induced reduction in neurogenesis and increase in apoptosis. (a-i) Co-treatment of COX-2 inhibitor (CAS 416901-58-1) with IL1β, IL6, or IFN-α, but not with EPA or DHA, did not prevent the effect of EPA and DHA against cytokines-induced reduction in DCX+cells, Map2+cells, and increase in CC3+cells. One-way ANOVA with Bonferroni's post hoc test was performed. Data are shown as mean±SEM; *p<0.05, **p<0.01, ***p<0.001, compared with vehicle treatment or as indicated.

Supplementary Figure 4. Co-treatment with LOX or CYP450 inhibitor and cytokines prevents the effect of EPA and DHA against cytokines-induced reduction in neurogenesis and increase in apoptosis. (a-i) Co-treatment of LOX inhibitor (NDGA) with IL1β, IL6, or IFN-α, but not with EPA or DHA prevented the effect of EPA and DHA against cytokines-induced reduction in DCX+cells, Map2+cells, and increase in CC3+cells. **(j-r)** Co-treatment of CYP450 inhibitor (SKF525A) with IL1β, IL6, or IFN-α, but not with EPA or DHA prevented the effect of EPA and DHA against cytokines-induced reduction in DCX+cells, Map2+cells, and increase in CC3+cells. One-way ANOVA with Bonferroni's post hoc test was performed. Data are shown as mean±SEM; *p<0.05, **p<0.01, ***p<0.001, compared with vehicle treatment or as indicated.

Supplementary Figure 5. Treatment with lower concentrations of LOX or CYP450 hydroxy lipid mediators prevents cytokines-induced reduction in neurogenesis and increase in apoptosis. (a-i) Co-treatment of cells with lower concentrations of with 5-HEPE, 18-HEPE, 4-HDHA or 20-HDHA and either IL1β, IL6 or IFN-α prevented decrease in DCX+ and Map+cells, and the increase in CC3+cells caused by treatment with cytokines alone. One-way ANOVA with Bonferroni's post hoc test was performed. Data are shown as mean±SEM; *p<0.05, **p<0.01, ***p<0.001, compared with vehicle treatment or as indicated.

Supplementary Figure 6. Treatment with lower concentrations of CYP450 epoxy lipid mediators prevent cytokines-induced reduction in neurogenesis, but not increase in apoptosis. (a-i) Co-treatment of cells with lower concentrations of 17(18)-EpETE or 19(20)-EpDPA and IL1β, IL6 or IFN-α prevented decrease in DCX+ and Map+cells, but not the increase in CC3+cells caused by treatment with cytokines alone, and this effect was enhanced by treatment with the sHE inhibitor TPPU. However, co-treatment of cells with 17(18)-DiHETE or 19(20)-DiHDPA did not prevent decrease in DCX+ and Map+cells, but not the increase in CC3+cells caused by treatment with cytokines alone. One-way ANOVA with Bonferroni's post hoc test was performed. Data are shown as mean±SEM; *p<0.05, **p<0.01, ***p<0.001, ****p<0.0001, compared with vehicle treatment or as indicated.

Supplementary Figure 7. Treatment with LOX or CYP450 hydroxy lipid mediators prevents cytokines-induced increase in downstream inflammatory and neurogenic pathways. (a-r) Co-treatment with 5-HEPE, 18-HEPE, 4-HDHA, 20-HDHA, 17(18)-EpETE or 19(20)-EpDPA and either IL1β, IL6 or IFN-α prevented the increase in STAT1 and NF-kB, and the decrease in AQP4 gene expression caused by treatment with cytokines alone. Moreover, co-treatment with 17(18)-EpETE or 19(20)-EpDPA, IL1β, IL6 or IFN-α, and sHE inhibitor further enhanced the effect of 17(18)-EpETE and 19(20)-EpDPA. One-way ANOVA with Bonferroni's post hoc test was performed. Data are shown as mean±SEM; *p<0.05, **p<0.01, ***p<0.001, ****p<0.0001, compared with vehicle treatment or as indicated.

Supplementary Figure 8. Peripheral production of additional COX, LOX and CYP450 lipid mediators in the same cohort of depressed patients receiving nutritional intervention with either EPA or DHA. (a-i) Additional lipid mediators identified in plasma of patients before and after receiving EPA; **(j-r)** additional lipid mediators identified in plasma of patients before and after receiving DHA. Wilcoxon's t test, with Bonferroni's post hoc test was performed. Data are shown as mean±SEM. Statistical analysis showed no significant differences in the expression of these metabolites. Legend: HDHA, hydroxydocosahexaenoic acid; oxo-ETE, oxo-eicosatetraenoic acid; HETE, hydroxyeicosatetraenoic acid; OxoODE, oxoode; HODE, hydroxyoctadecadienoic acid; Trans EKODE, epoxy-keto-octadecenoic acid; HOTrE, octadecatrienoic acid; HETrE, hydroxyeicosatrienoic acid; EpDPA, epoxydocosapentaenoic acid; DiHDPA, dihydroxydocosapentaenoic acid; EET, epoxyeicosatrienoic acid; DHET, dihydroxyeicosatrienoic acid; EpOME, epoxyoctadecenoic acid; DiHOME, dihydroxyoctadecenoic acid; PG, prostaglandin; TXB, thromboxane; HEPE, hydroxyeicosapentaenoic acid; EpETE, epoxyeicosatetraenoic acid DiHETE, dihydroxyeicosatetraenoic acid. Lower limit of detection was reported for 5HETrE, 16(17), 19(20)-EpDPA, 5(6), 8(9), 14(15)-EET, 15-HEPE, 11(12), 14(15), 17(18)-EpETE, 8(9)-DiHETE, 5(6), 8(9), 14(15)-EET. Lower limit of detection was reported for 5HETrE, 16(17), 19(20)-EpDPA, 5(6), 8(9), 14(15)-EET, 15-HEPE, 11(12), 14(15), 17(18)-EpETE, 8(9)-DiHETE, 5(6), 8(9), 14(15)-EET.

Supplementary Figure 9. Increased levels of LOX and CYP450 lipid mediators in depressed patients receiving ω-3 PUFAs intervention correlates with less severe depressive symptoms. (a-h) Correlations between levels of 5-, 8-, 9-, 11-, 12-, 15-, 18-HEPE, 8(9)-, 11(12)-, 14(15)-, 17(18)-EpETE, -DiHETE metabolites, and HAM-D scores. **(i-m)** Correlations between levels of 4-, 7-, 8-, 10-, 11-, 13-, 14-, 20-HDHA, 10(11)-, 13(14)-, 16(17)-, 19(20)-EpDPA, -DiHDPA metabolites and HAM-D scores. Spearman's r_s test was performed.
