# Supplementary figures and images for: Omega-3 polyunsaturated fatty acids protect against inflammation through production of LOX and CYP450 lipid mediators: relevance for major depression and for human hippocampal neurogenesis
Source: Mol Psychiatry. 2021 Jun 16;26(11):6773–88. doi: 10.1038/s41380-021-01160-8 (PMC8760043; doi:10.1038/s41380-021-01160-8)

a)

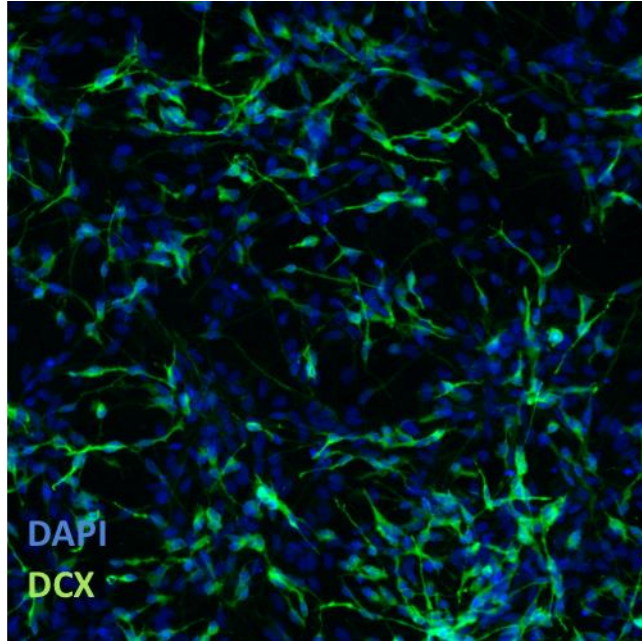

b)

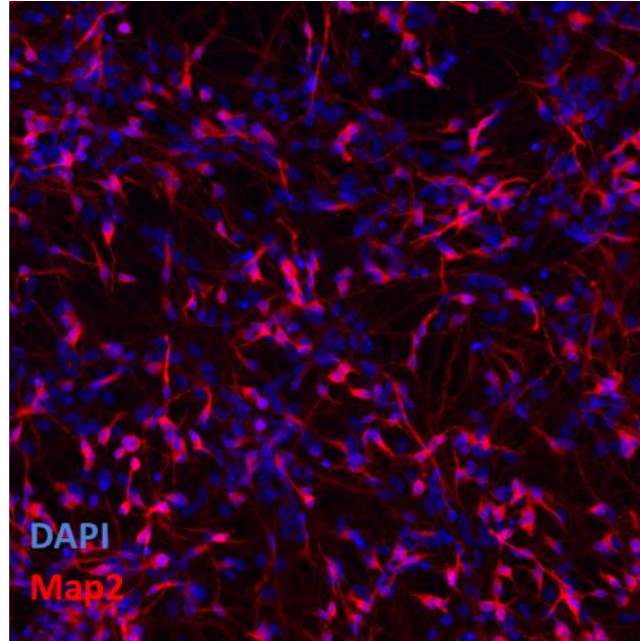

c)

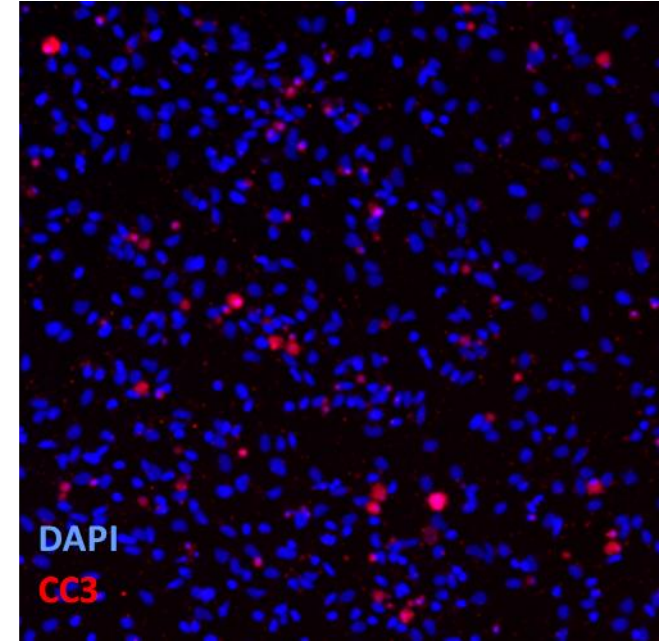

Supplement: Supplementary file 2 — Supplementary Figure 1 [file 41380_2021_1160_MOESM2_ESM.pdf]

a)

## DCX

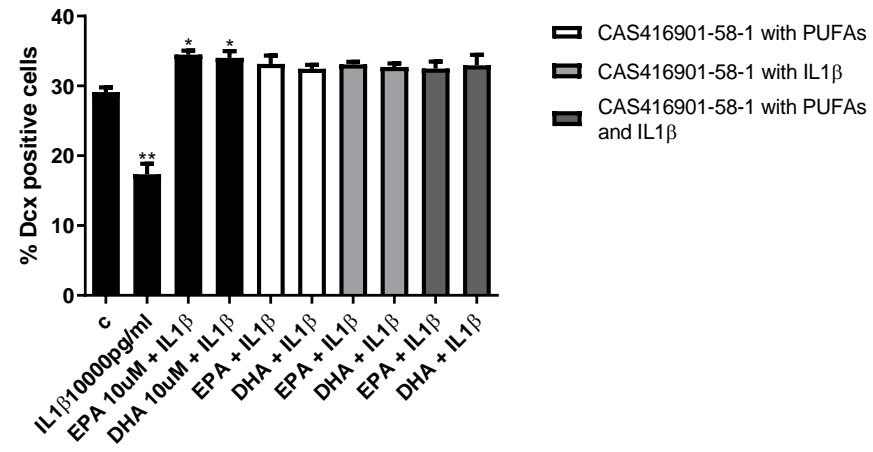

b)

## Map2

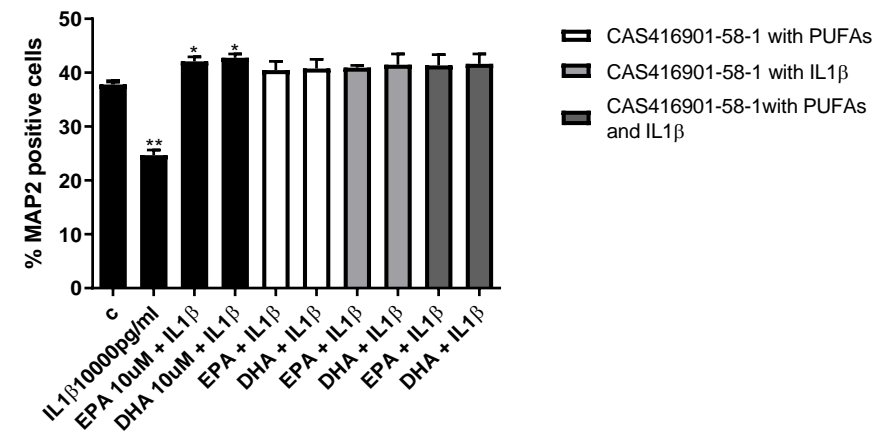

c)

## CC3

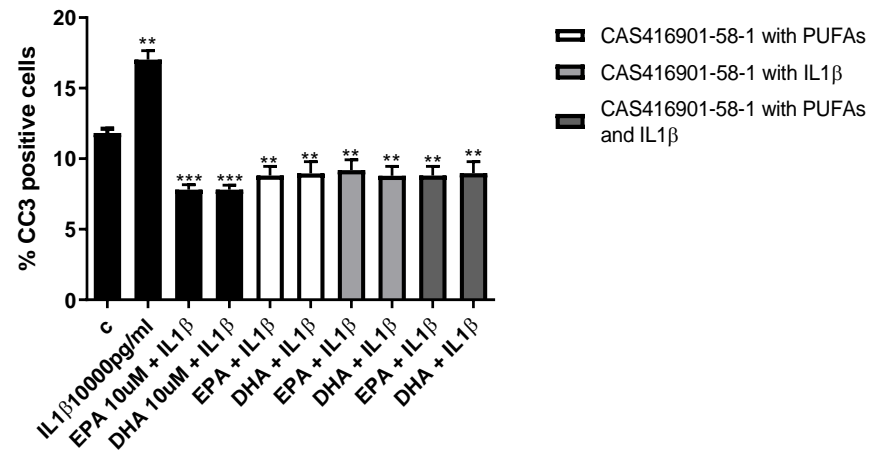

d)

Map2

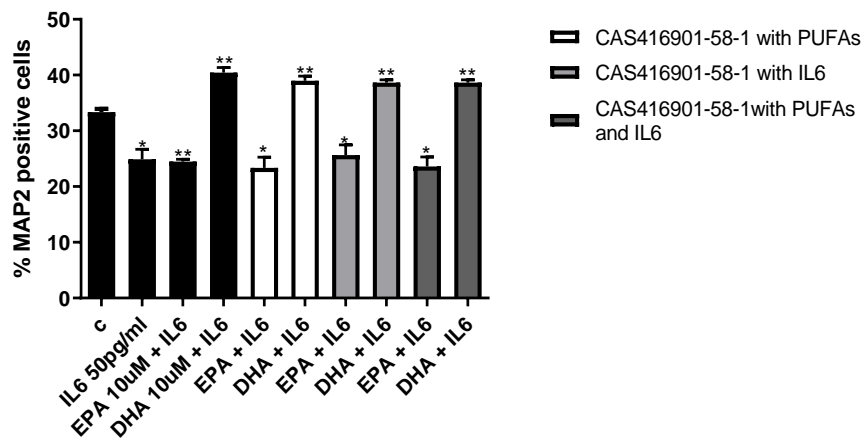

e)

DCX

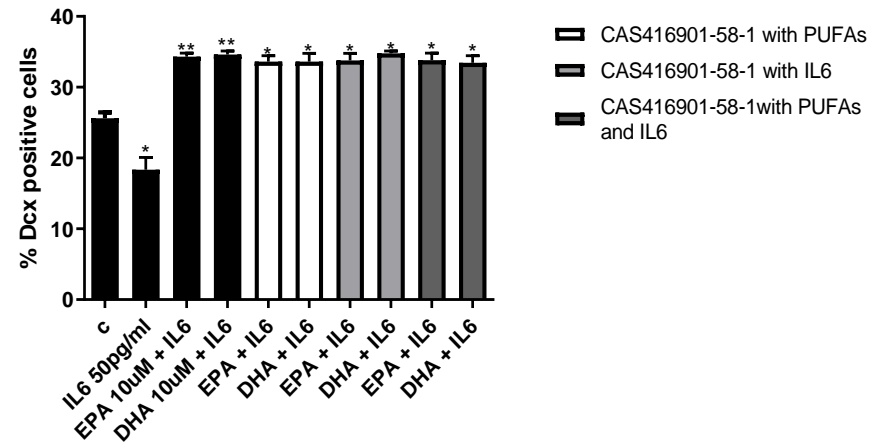

f)

CC3

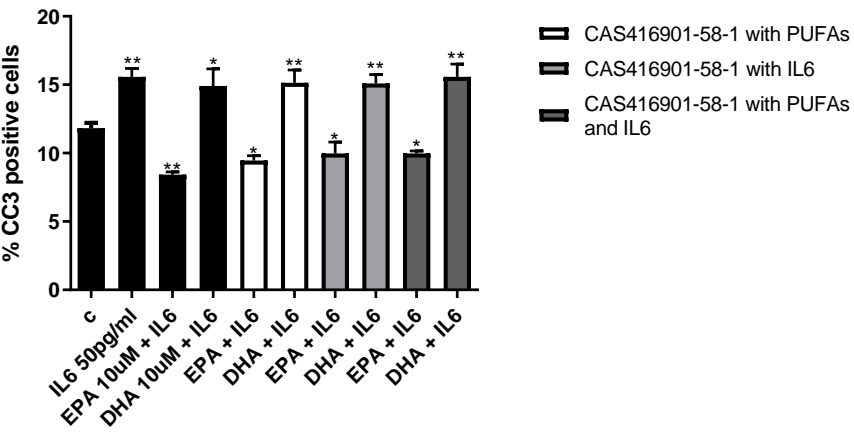

g)

DCX

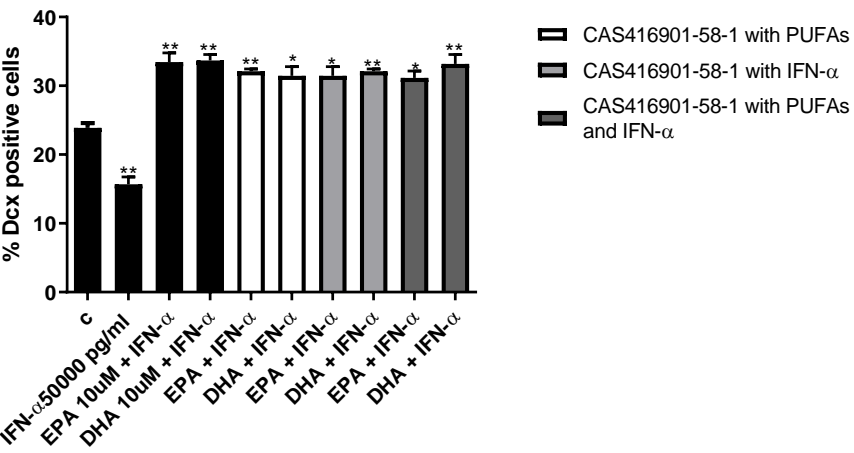

h)

Map2

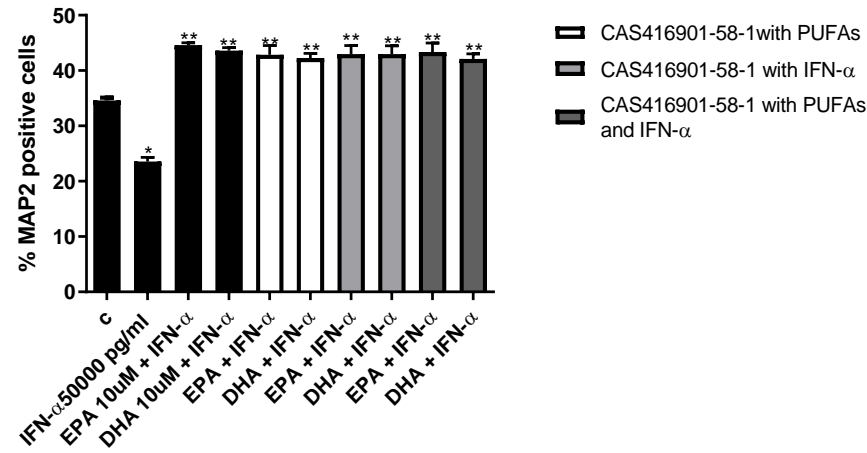

i)

CC3

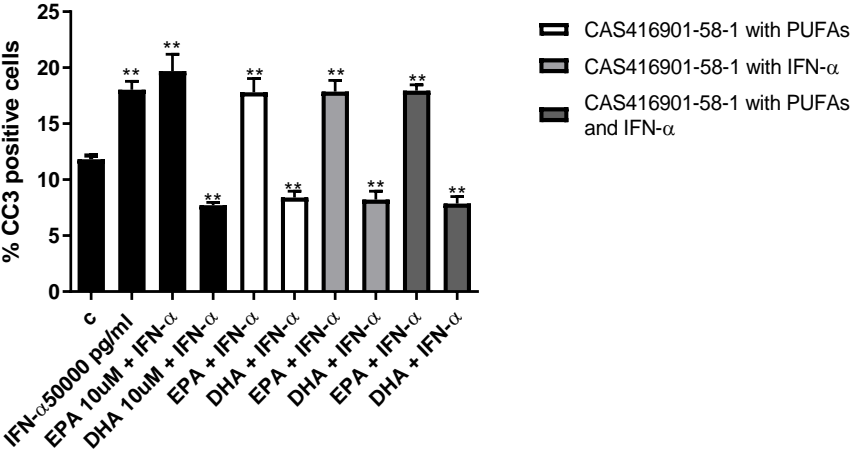

Supplement: Supplementary file 4 — Supplementary Figure 3 [file 41380_2021_1160_MOESM4_ESM.pdf]

a)

## DCX

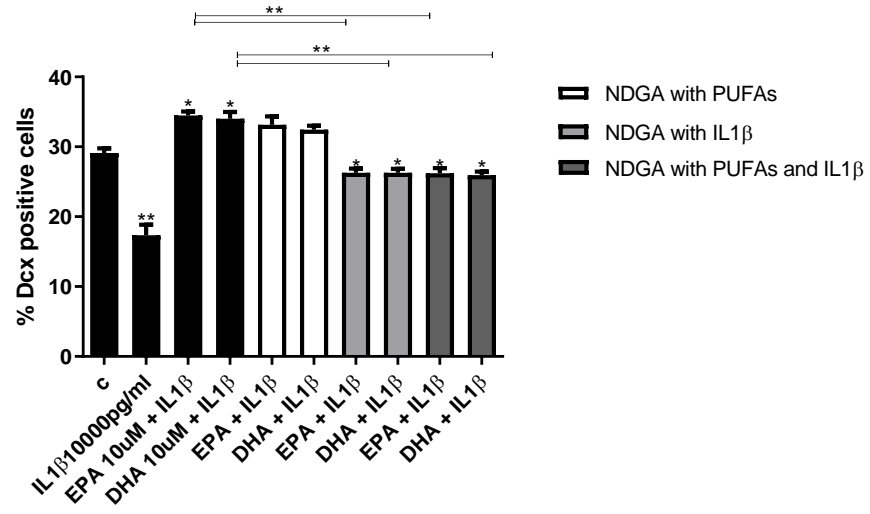

b)

## Map2

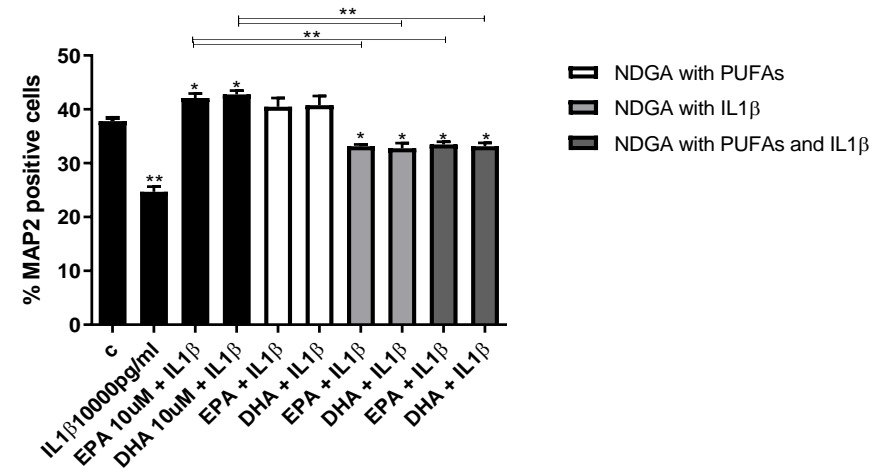

c)

## CC3

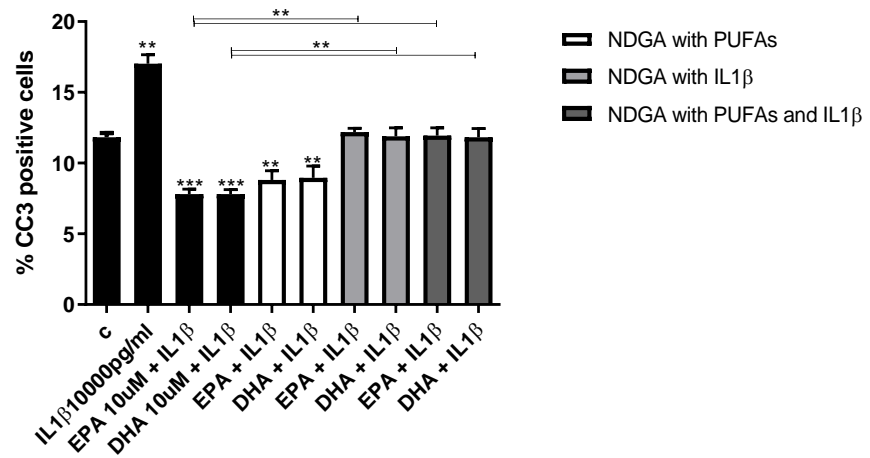

d)

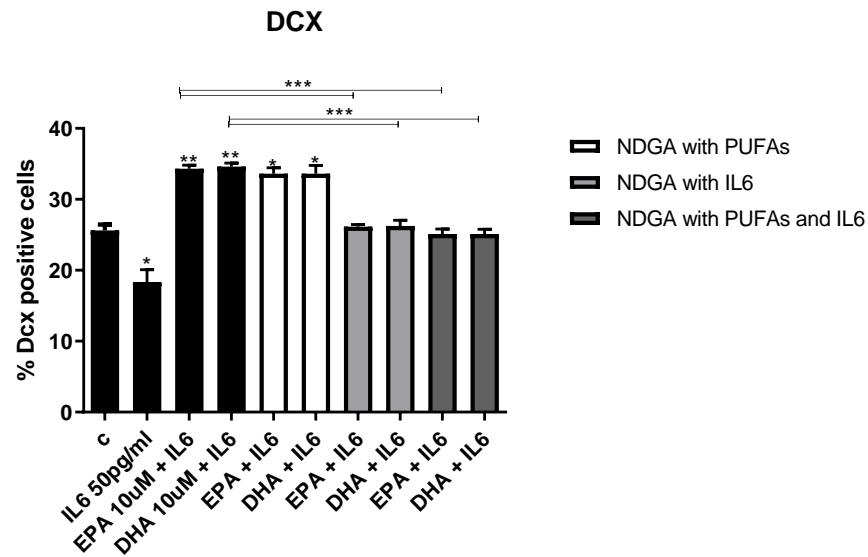

e)

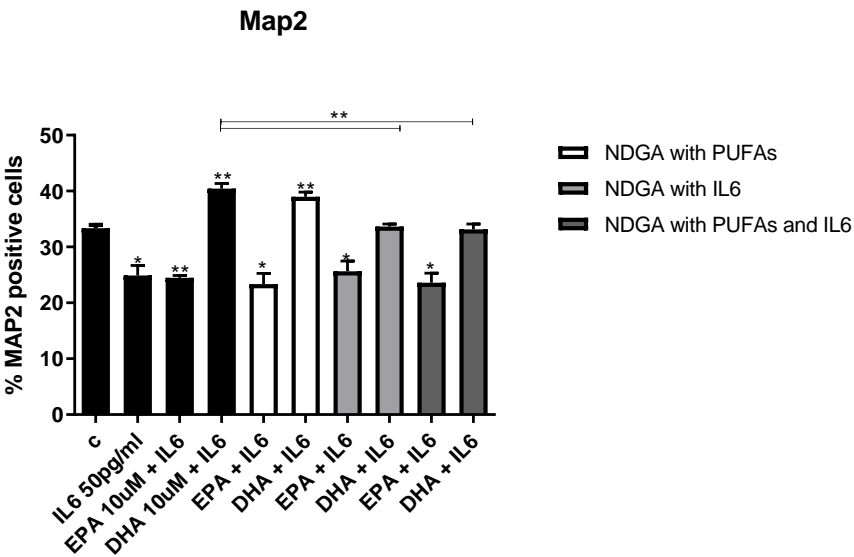

f)

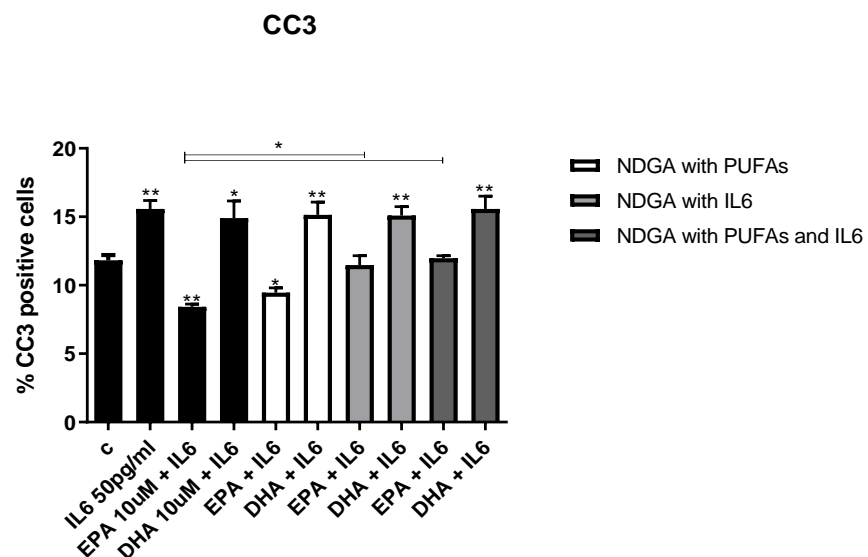

g)

## DCX

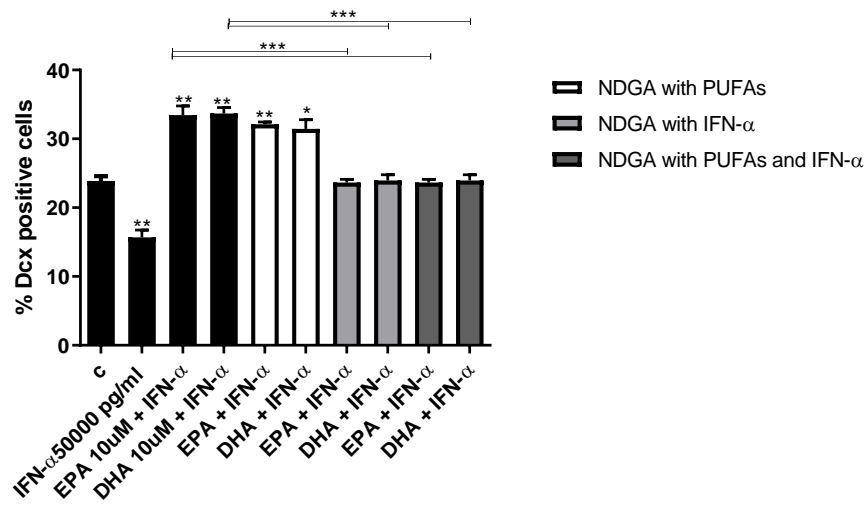

h)

## Map2

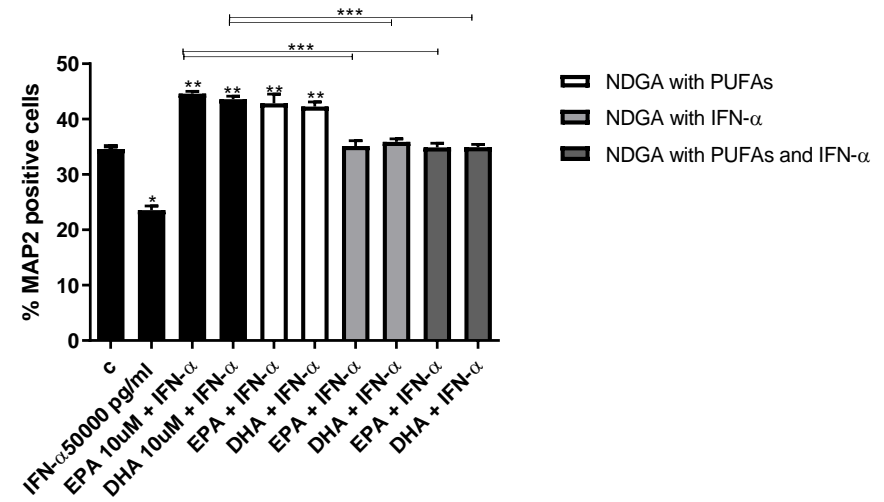

i)

## CC3

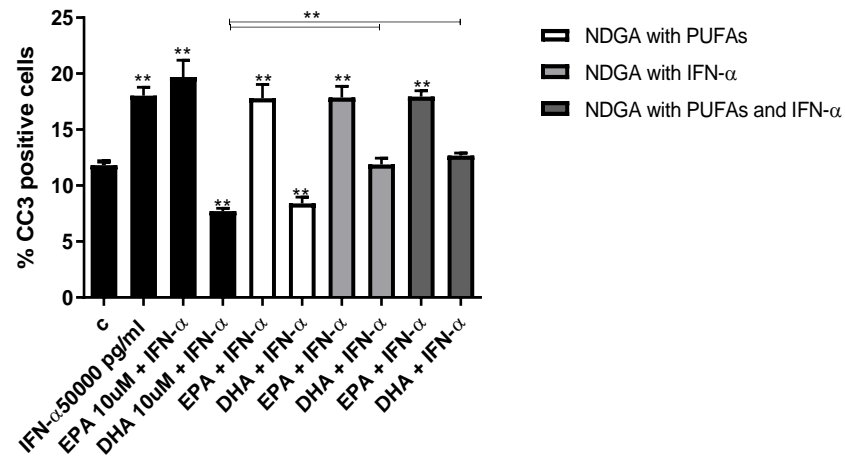

j)

## DCX

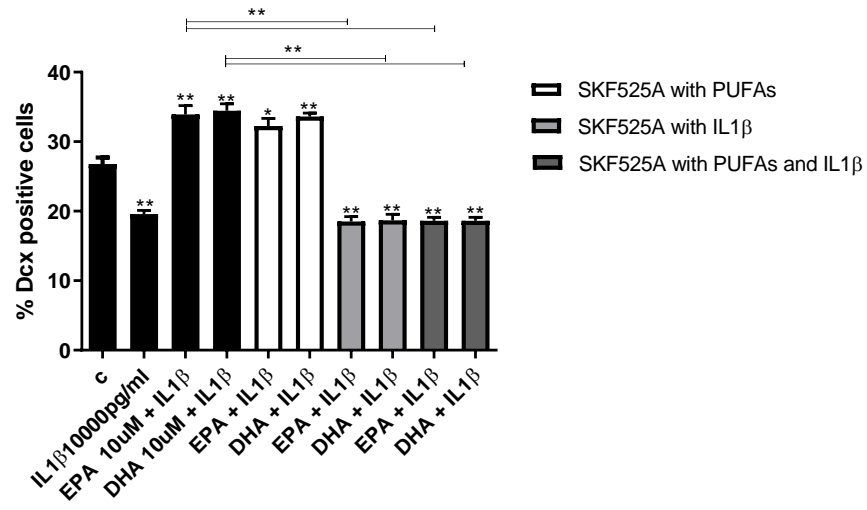

k)

## Map2

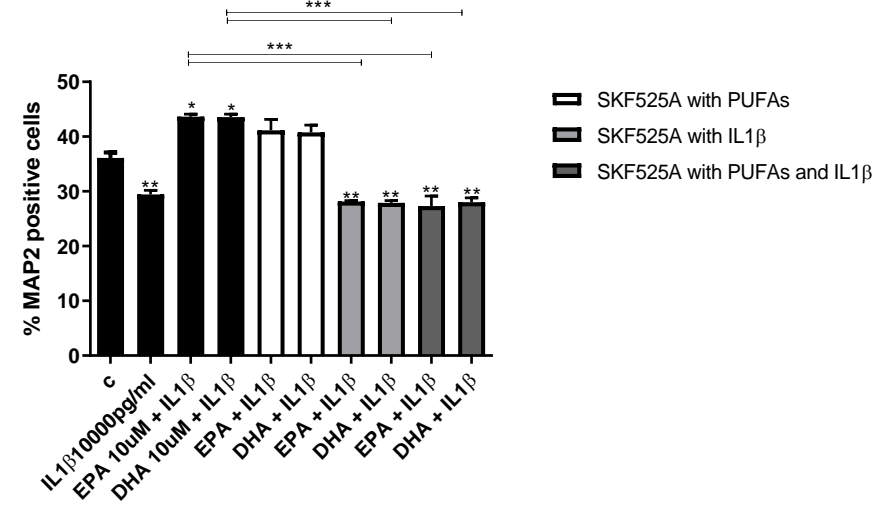

l)

## CC3

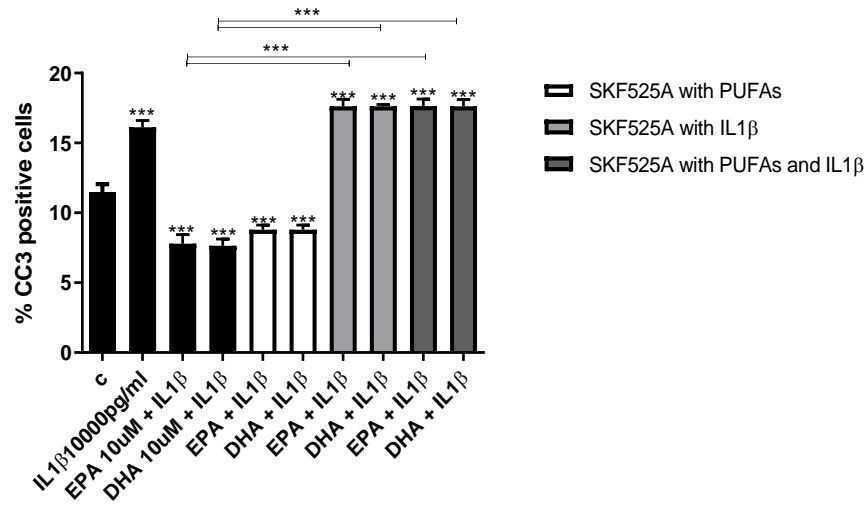

**m)**

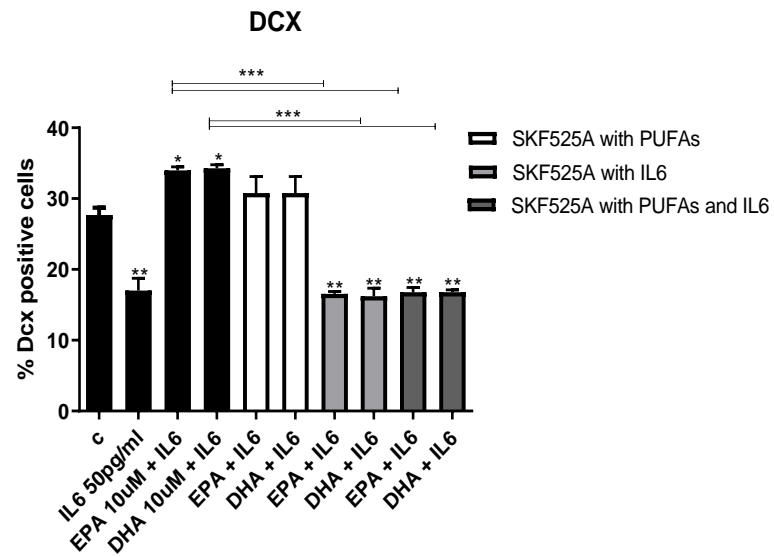

**o)**

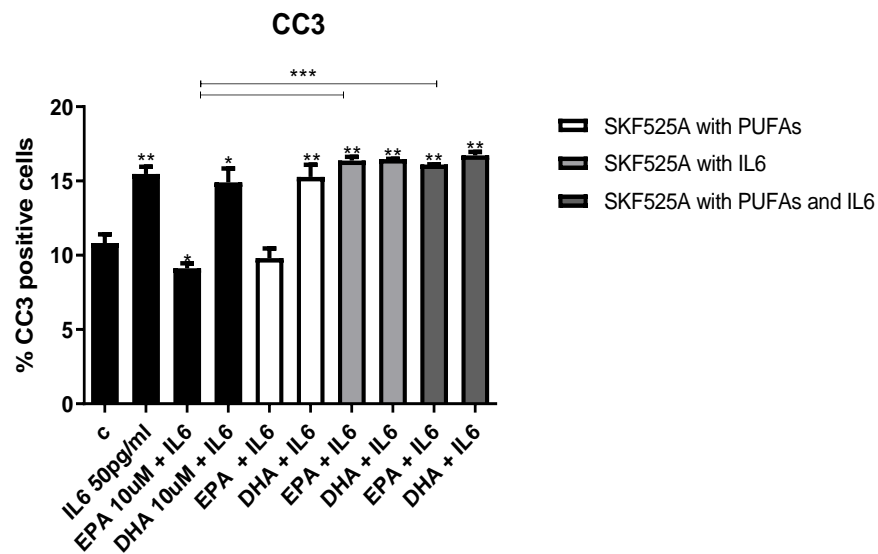

**n)**

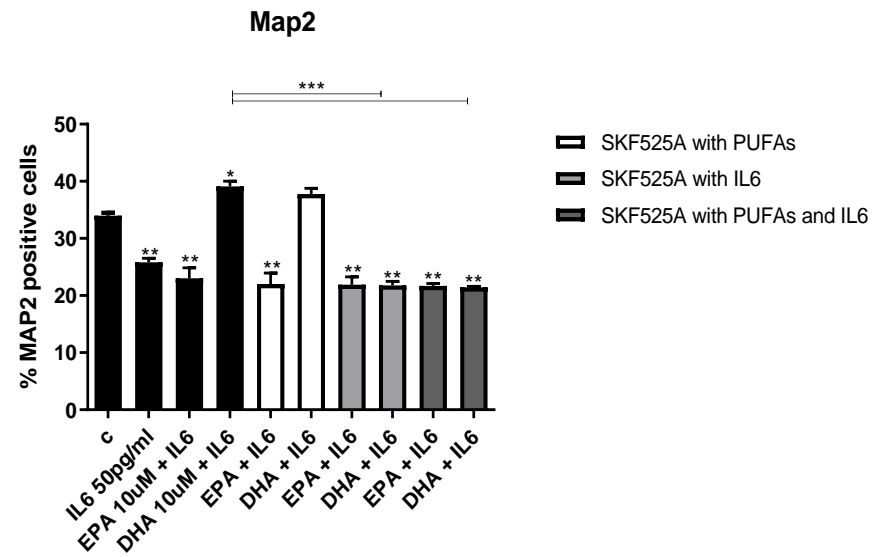

p)

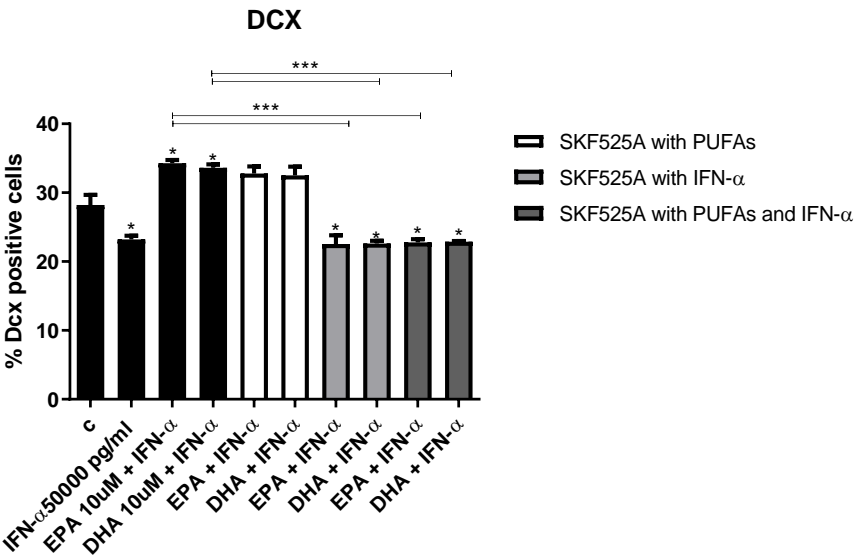

q)

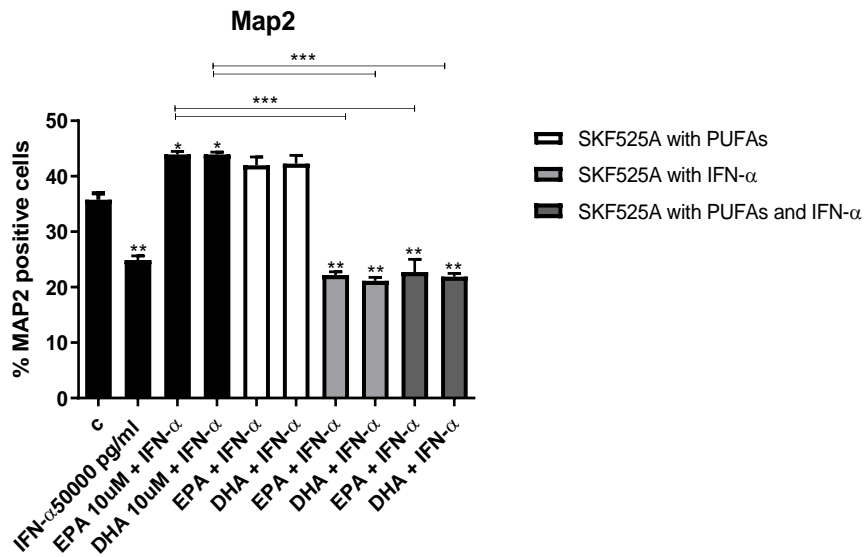

r)

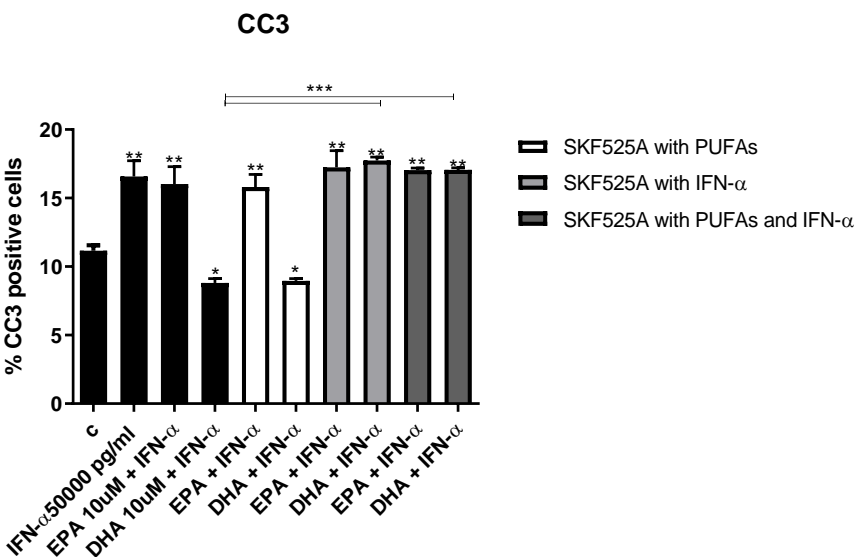

Supplement: Supplementary file 5 — Supplementary Figure 4 [file 41380_2021_1160_MOESM5_ESM.pdf]

a)

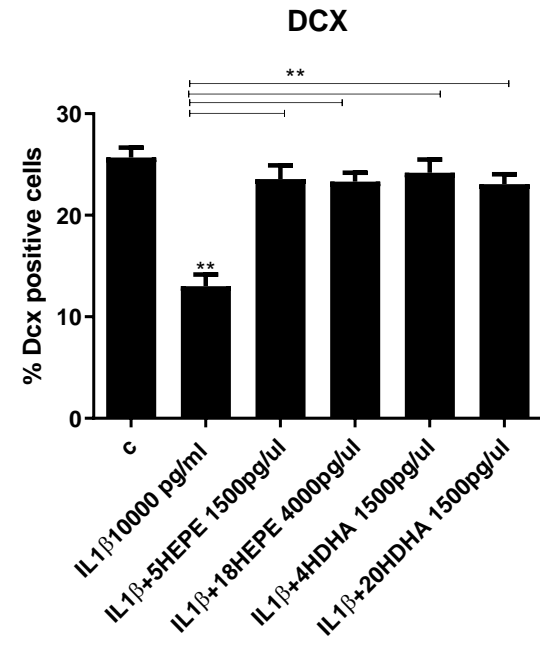

b)

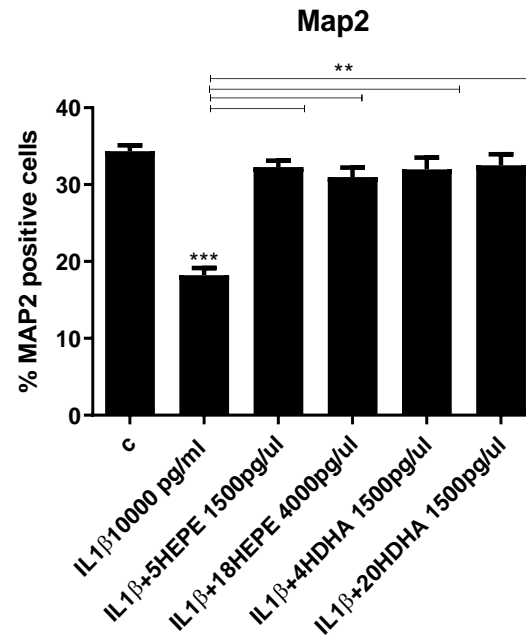

c)

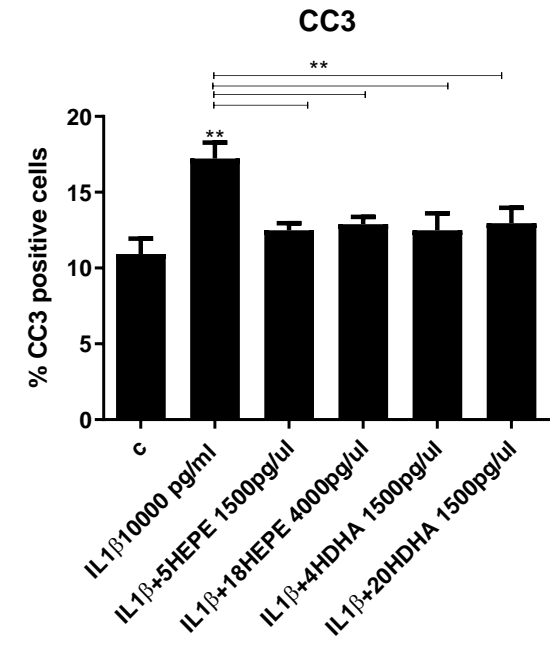

d)

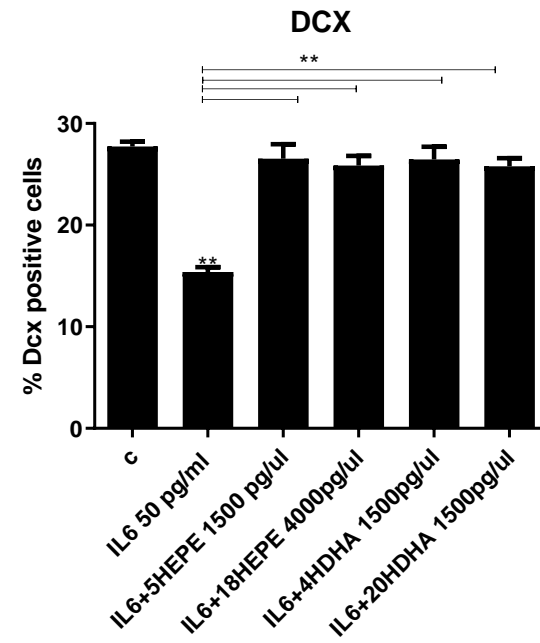

e)

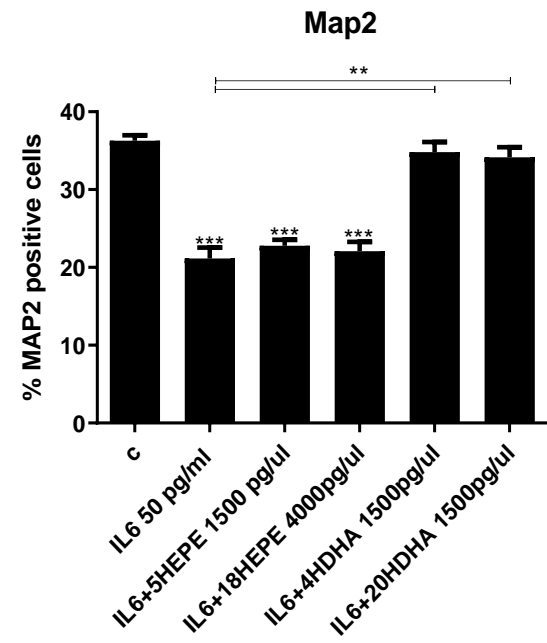

f)

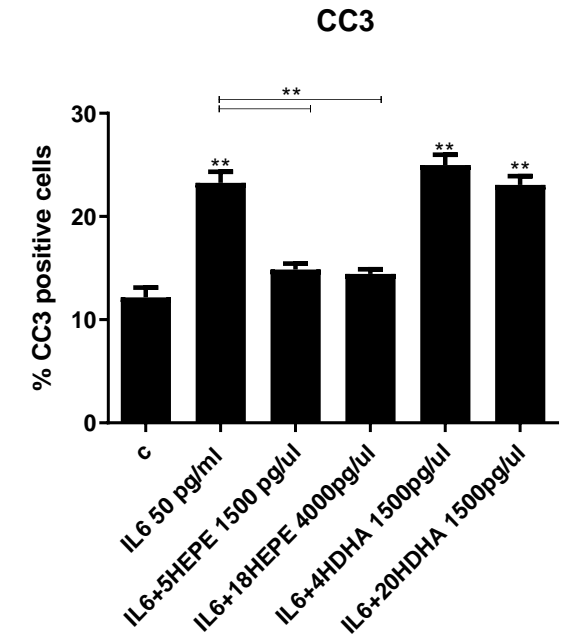

g)

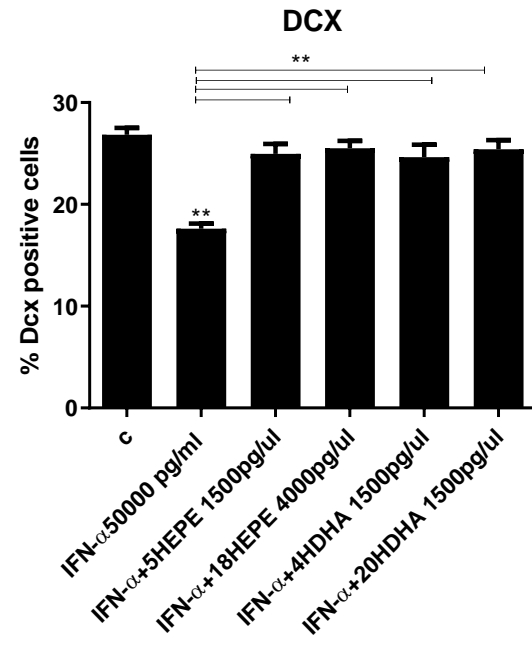

h)

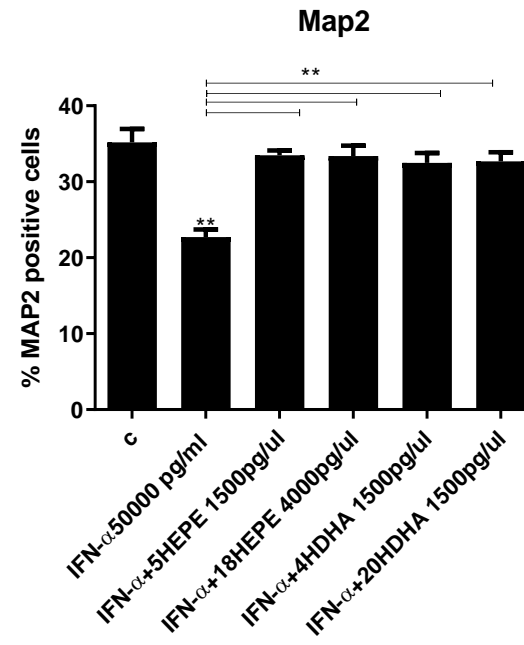

i)

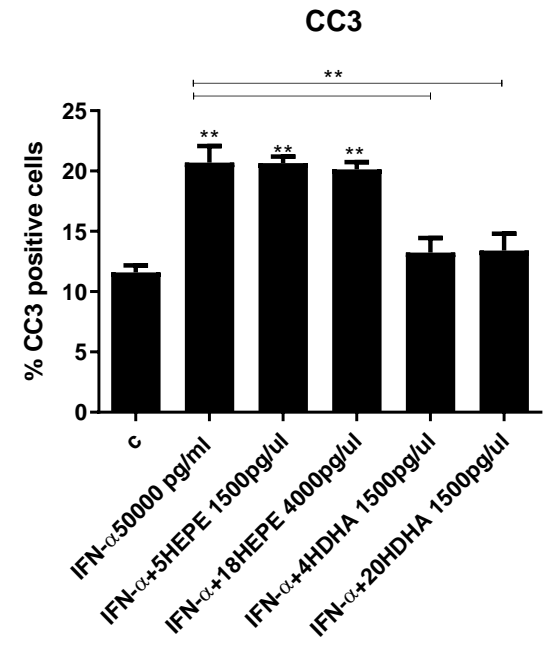

Supplement: Supplementary file 6 — Supplementary Figure 5 [file 41380_2021_1160_MOESM6_ESM.pdf]

a)

DCX

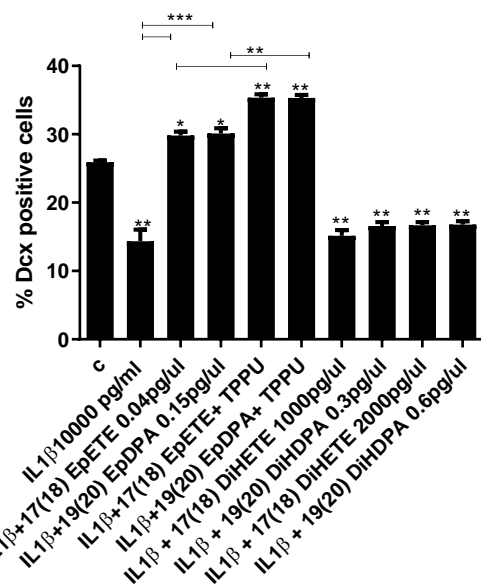

b)

Map2

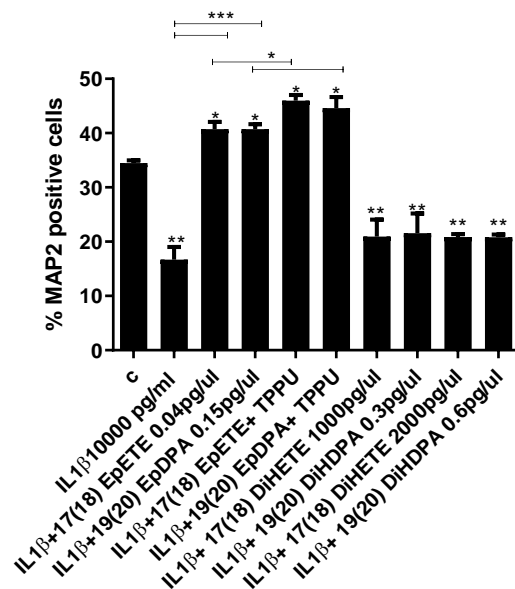

c)

CC3

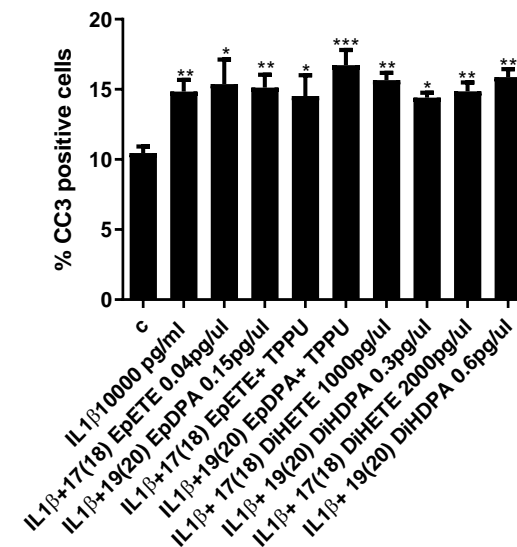

d)

DCX

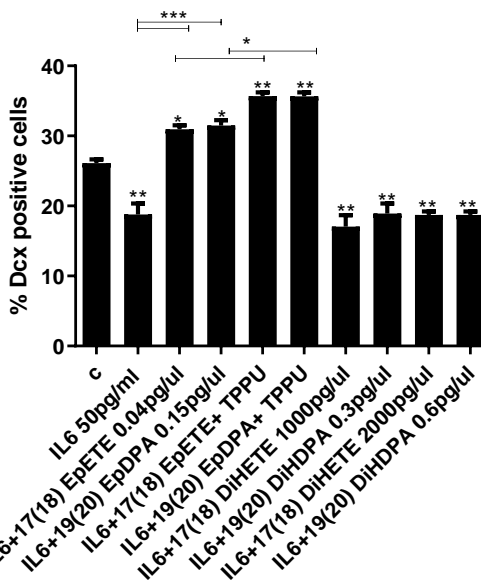

e)

Map2

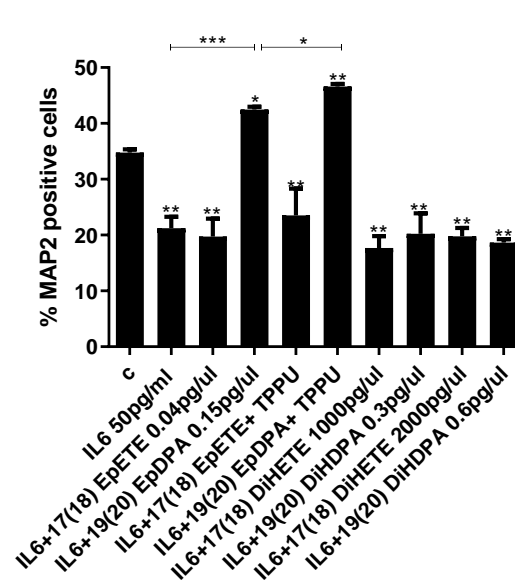

f)

CC3

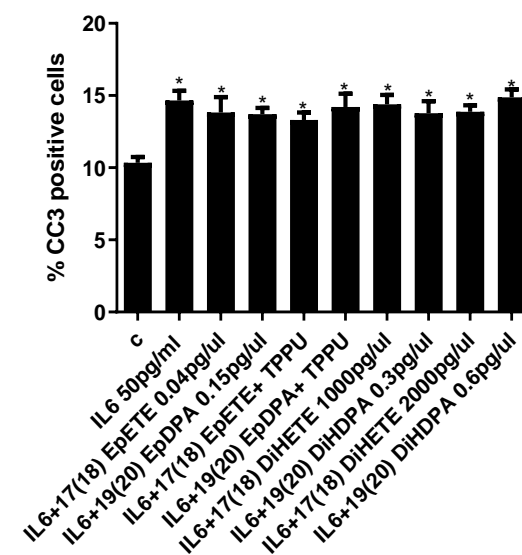

g)

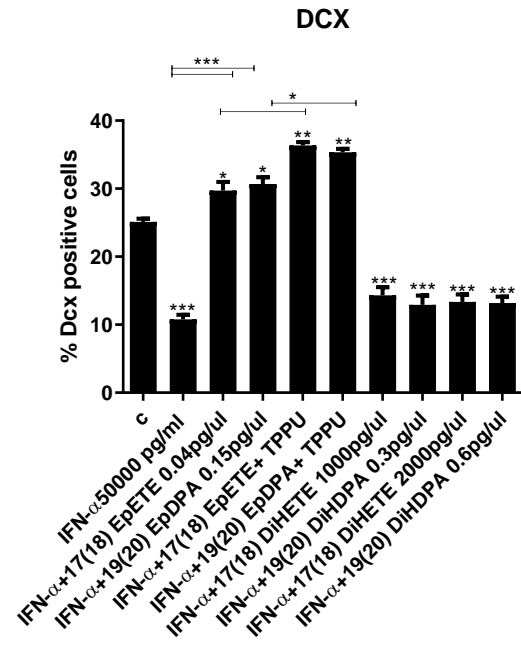

h)

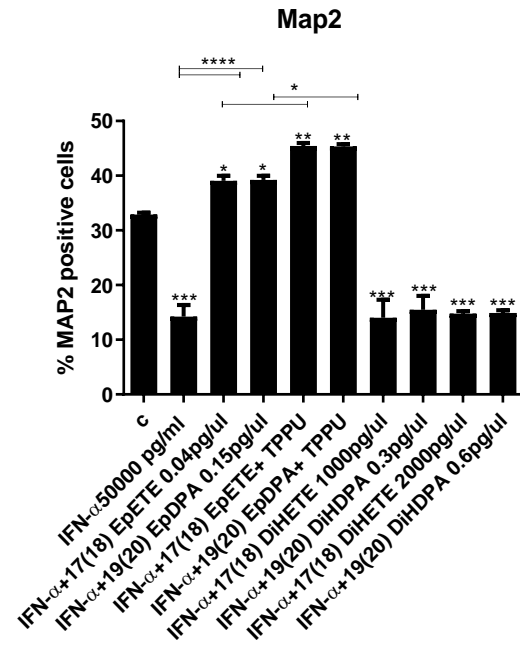

i)

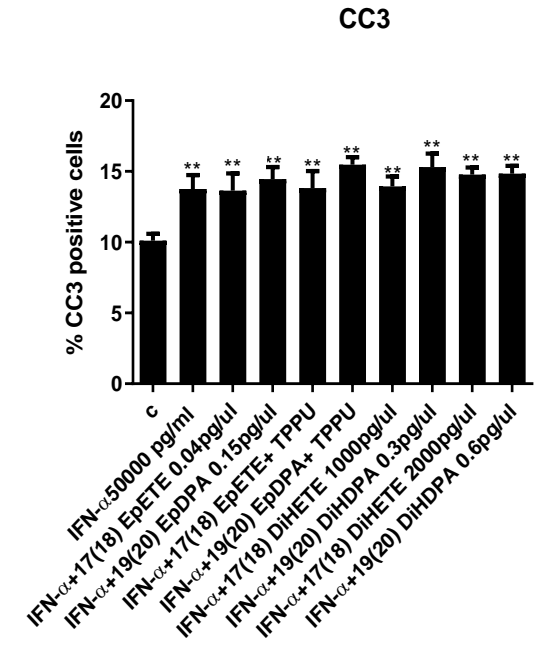

Supplement: Supplementary file 7 — Supplementary Figure 6 [file 41380_2021_1160_MOESM7_ESM.pdf]

a)

## STAT1

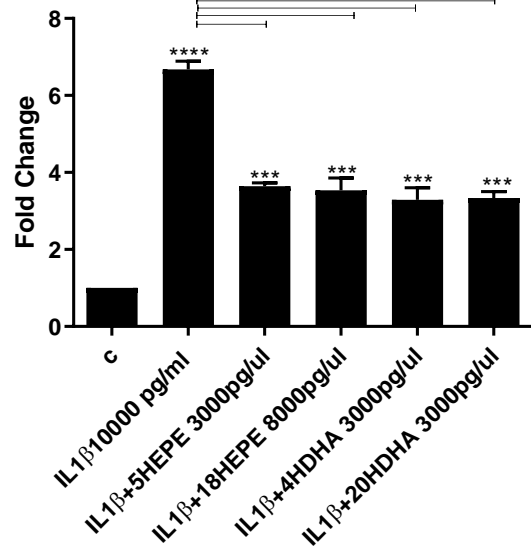

b)

## NFκB

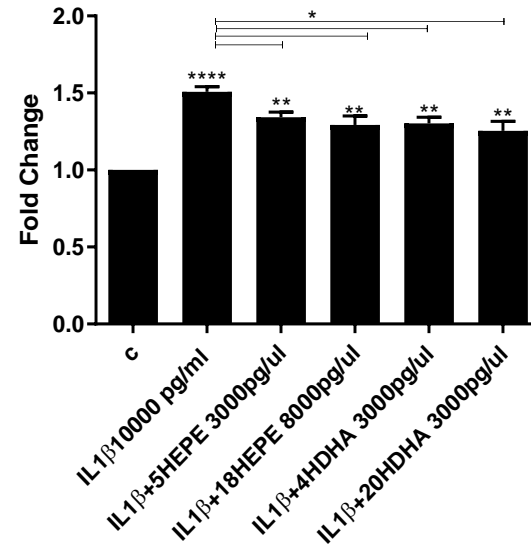

c)

## AQP4

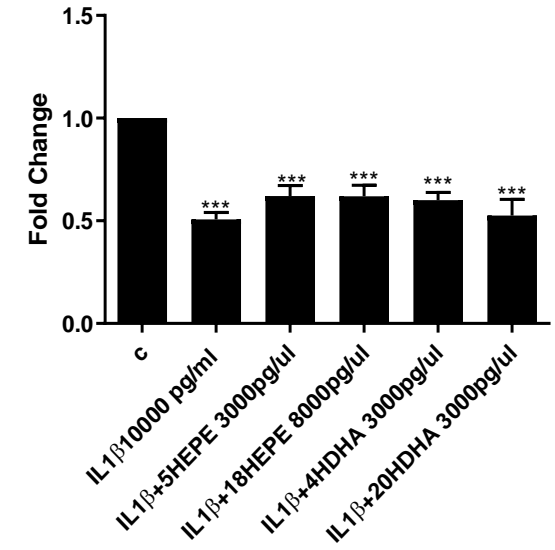

d)

## STAT1

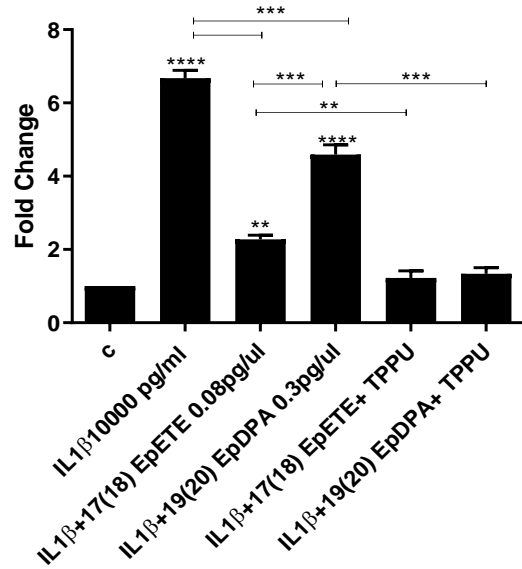

e)

## NFκB

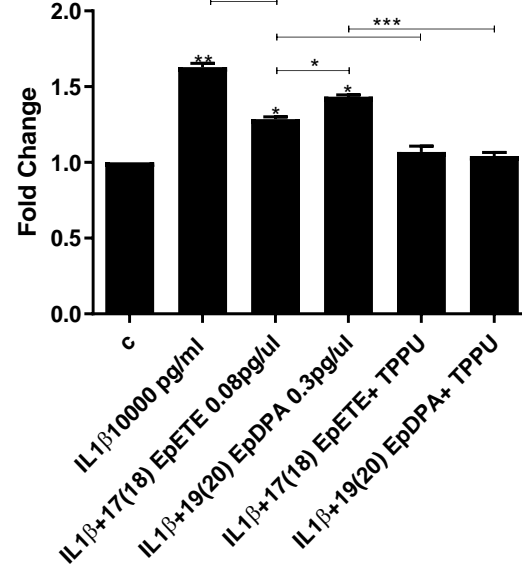

f)

## AQP4

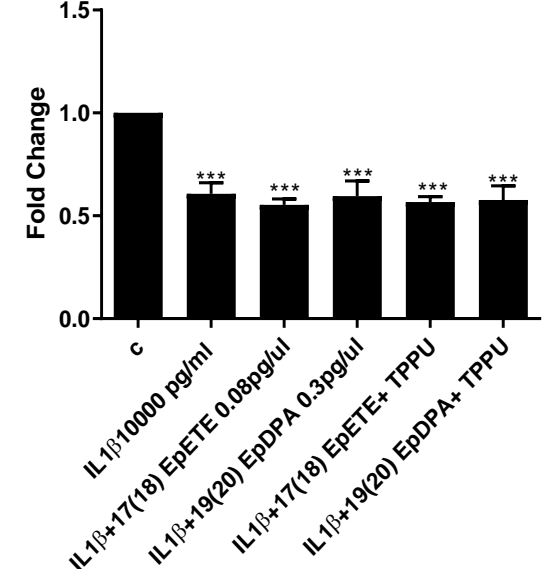

g)

STAT1

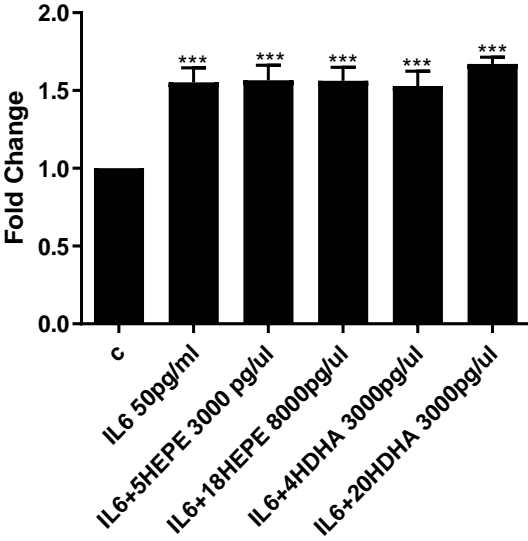

h)

NFKB

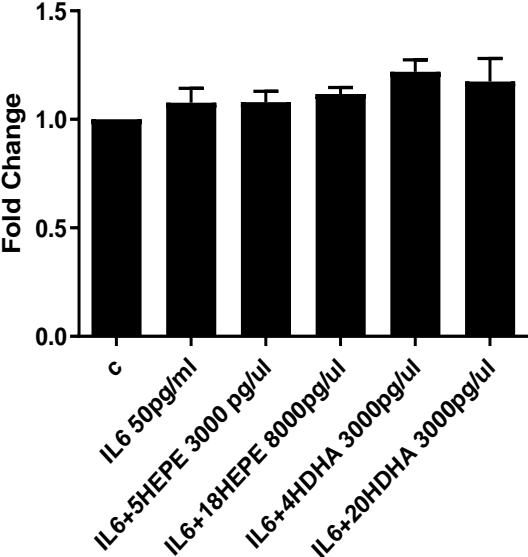

i)

AQP4

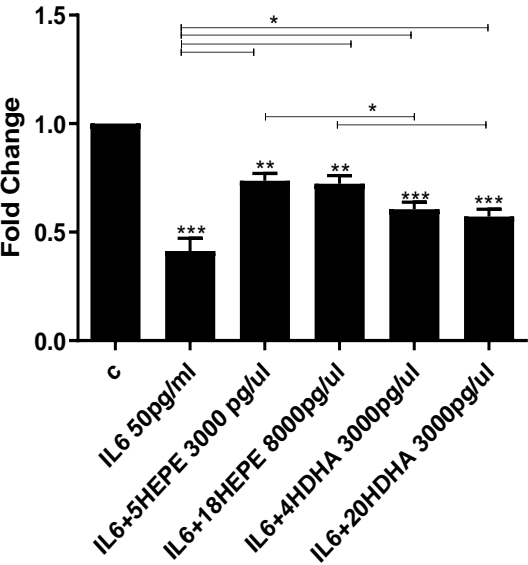

j)

STAT1

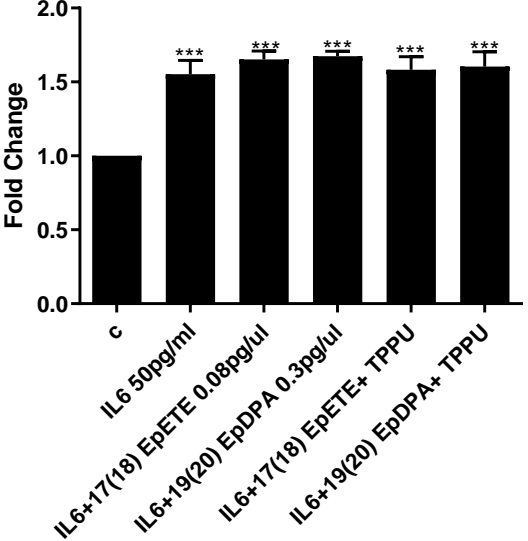

k)

NFKB

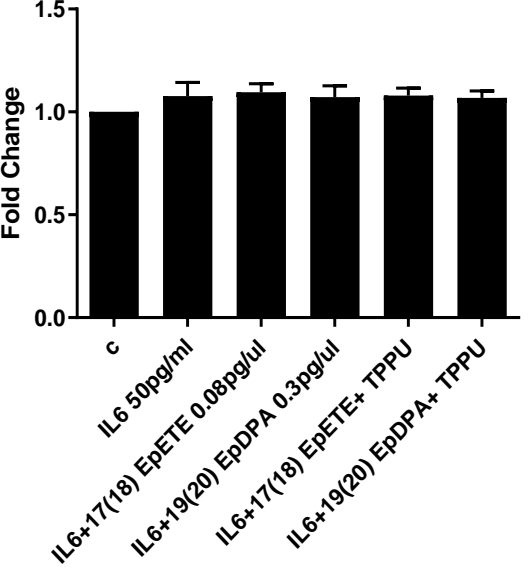

l)

AQP4

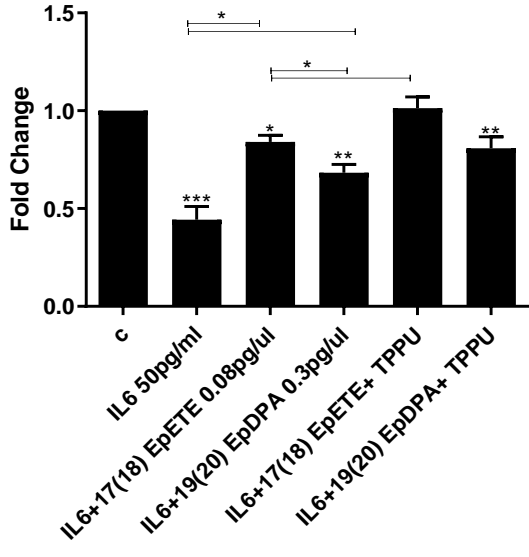

m)

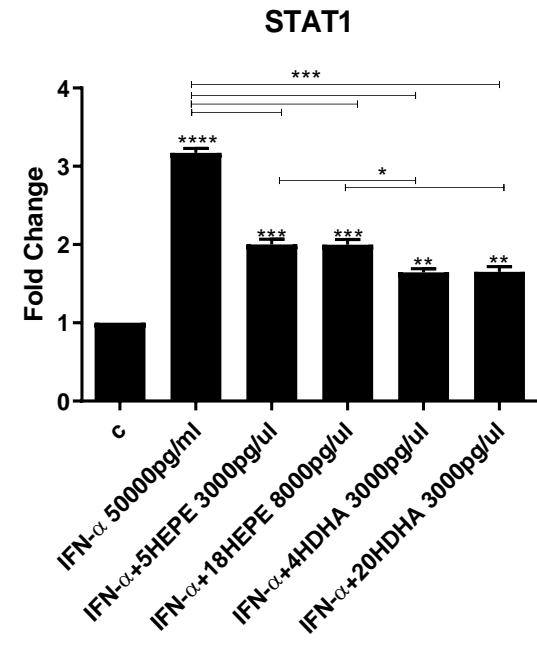

n)

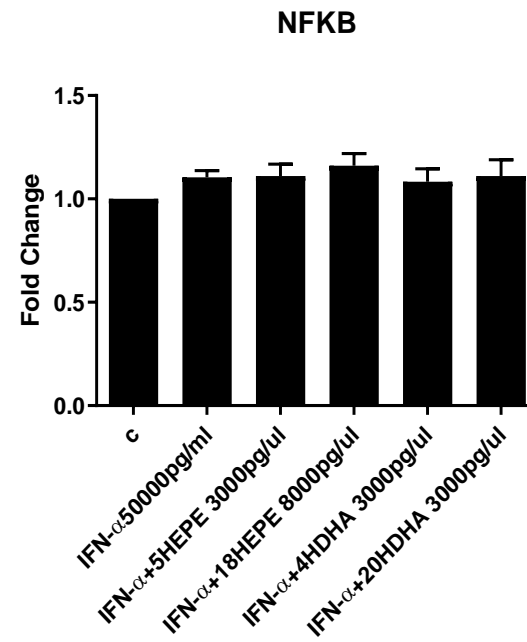

o)

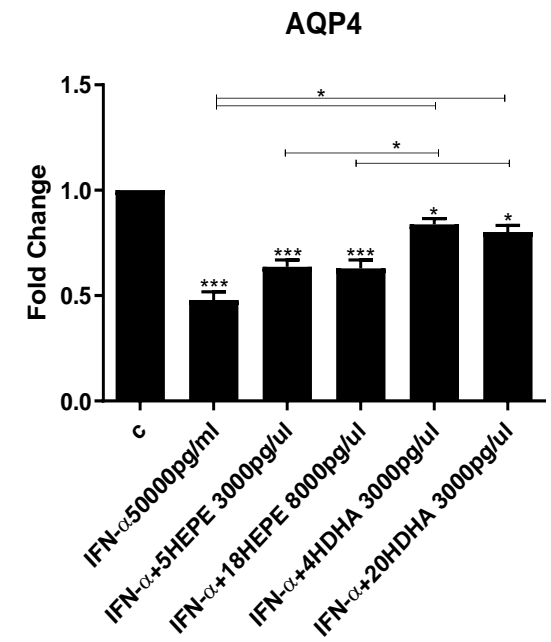

p)

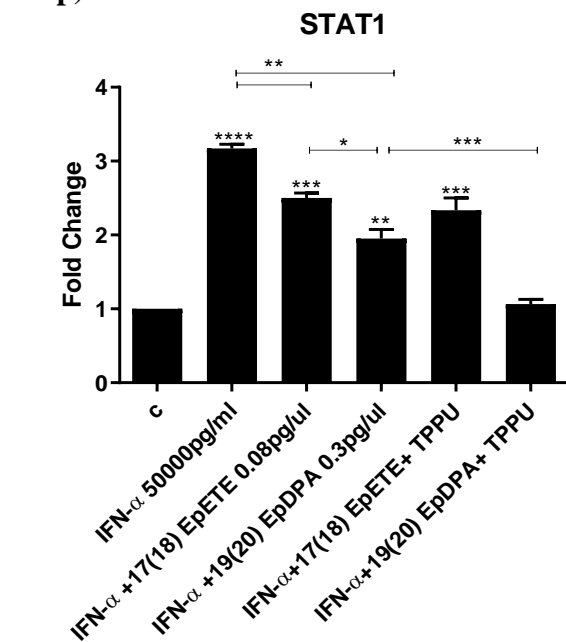

q)

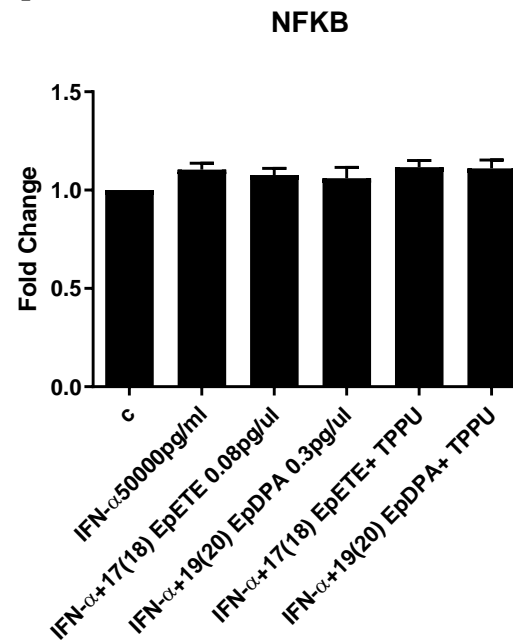

r)

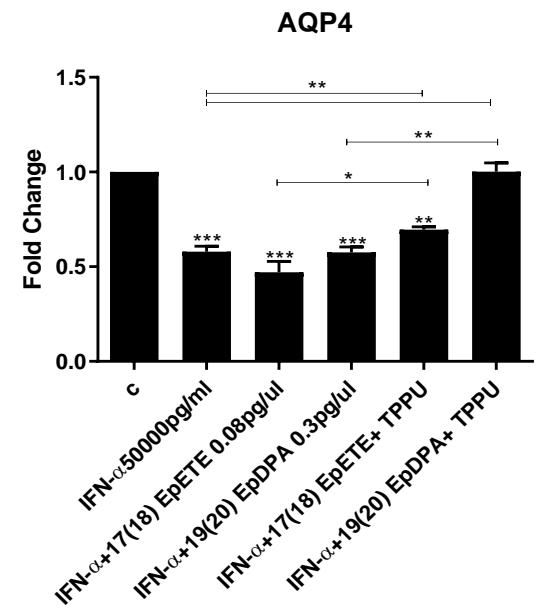

Supplement: Supplementary file 8 — Supplementary Figure 7 [file 41380_2021_1160_MOESM8_ESM.pdf]

a)

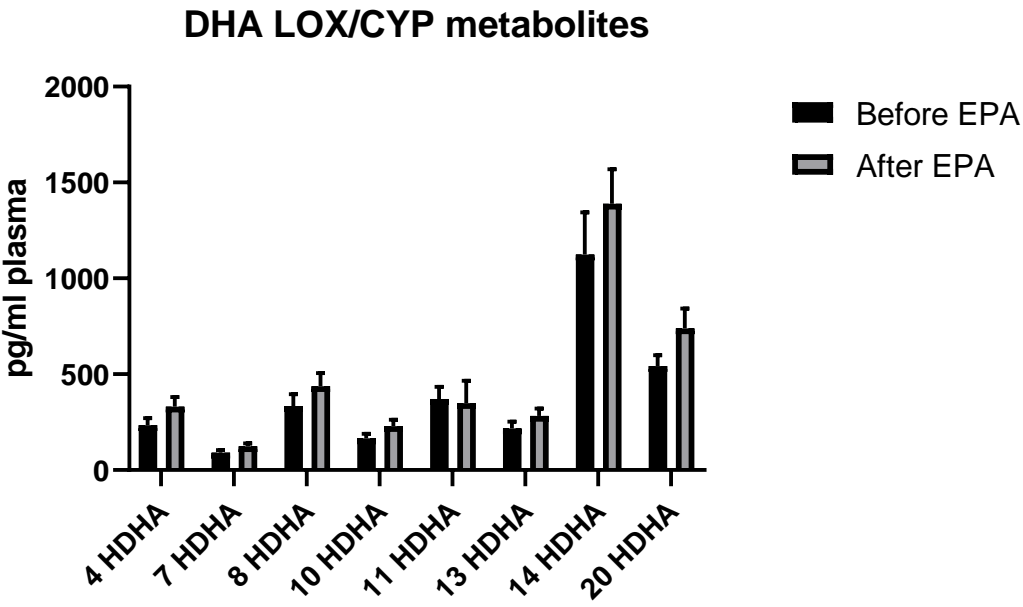

b)

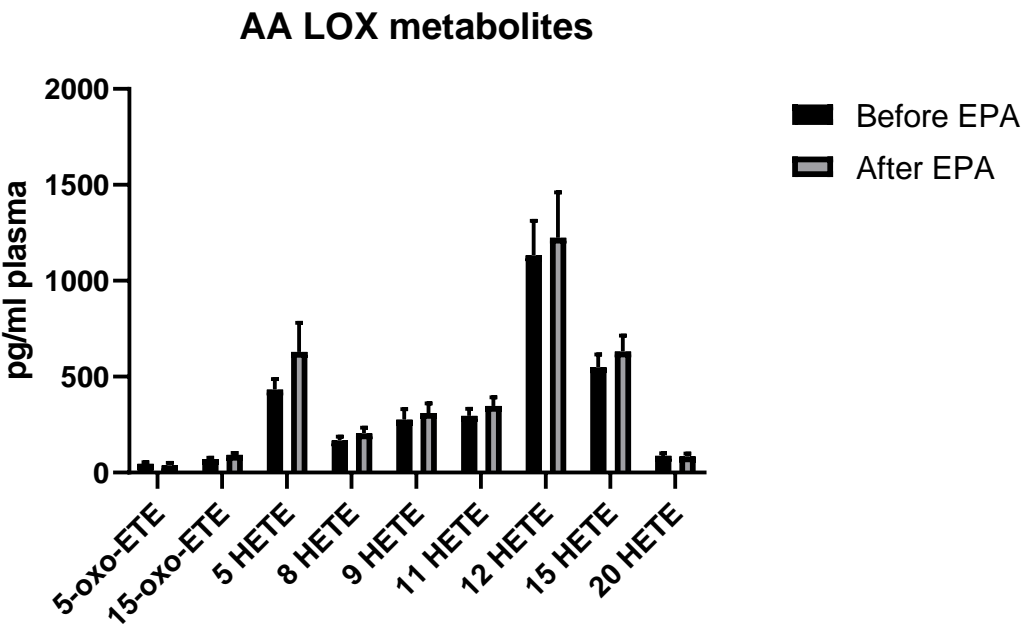

c)

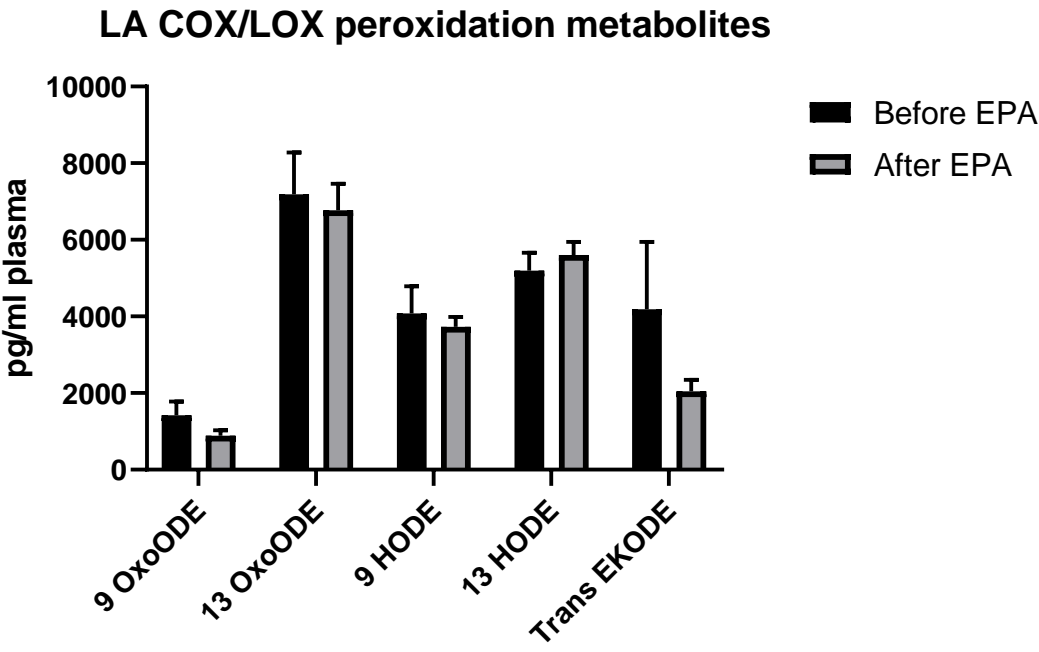

d)

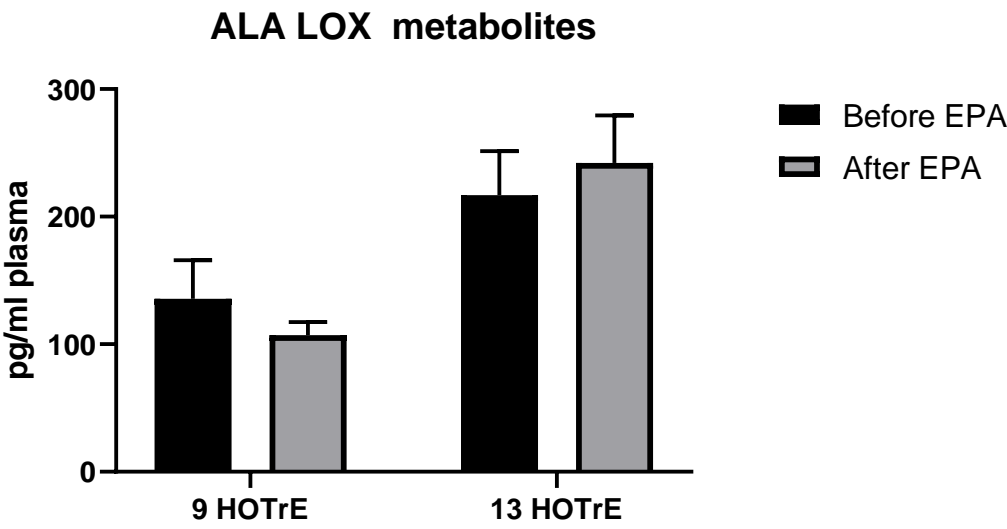

e)

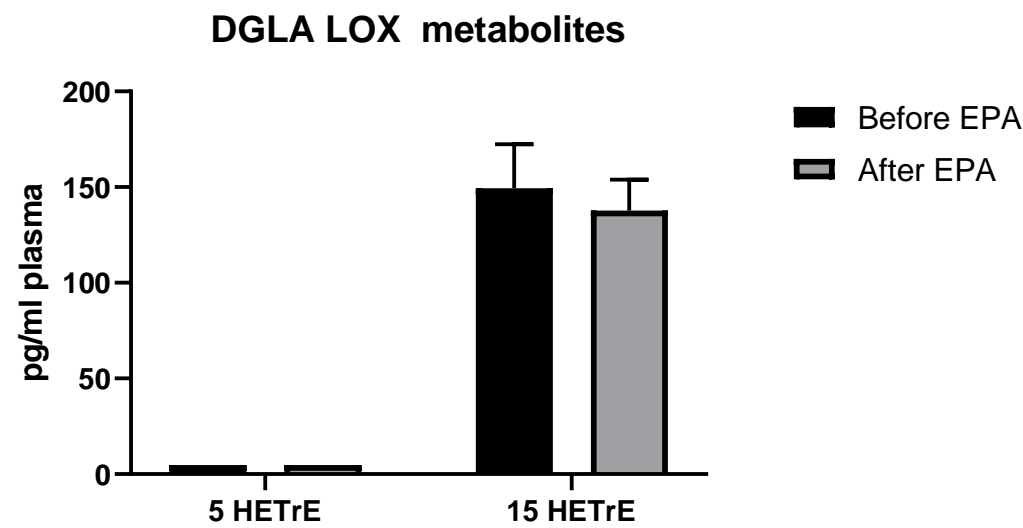

f)

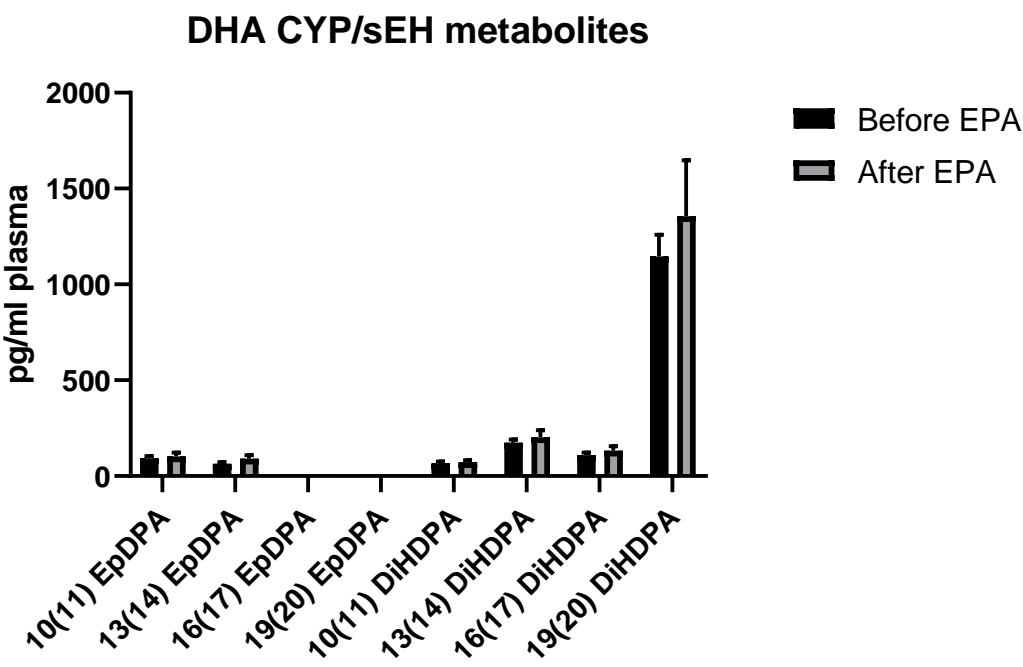

g)

**AA CYP/sEH metabolites**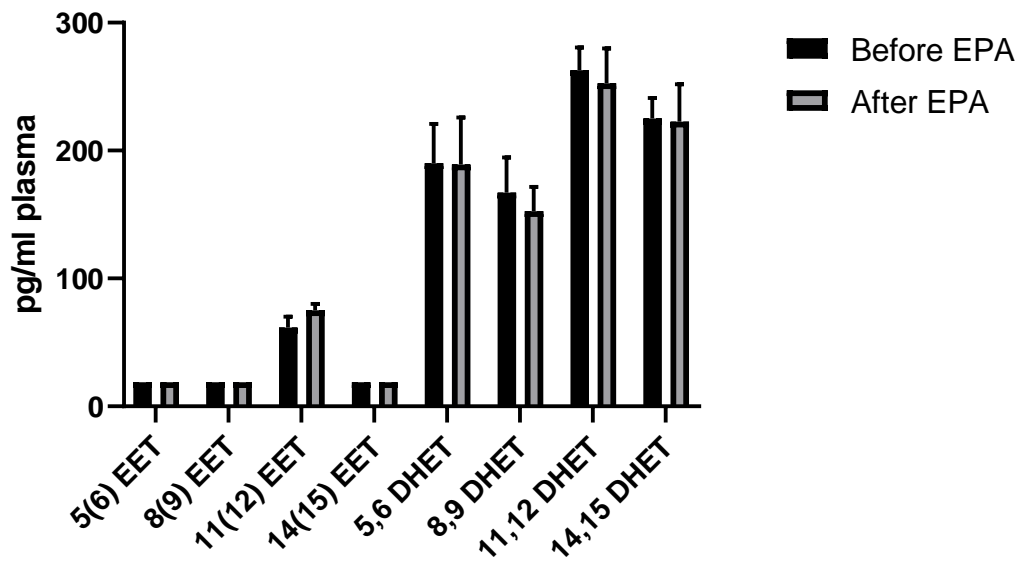

h)

**LA CYP/sEH metabolites**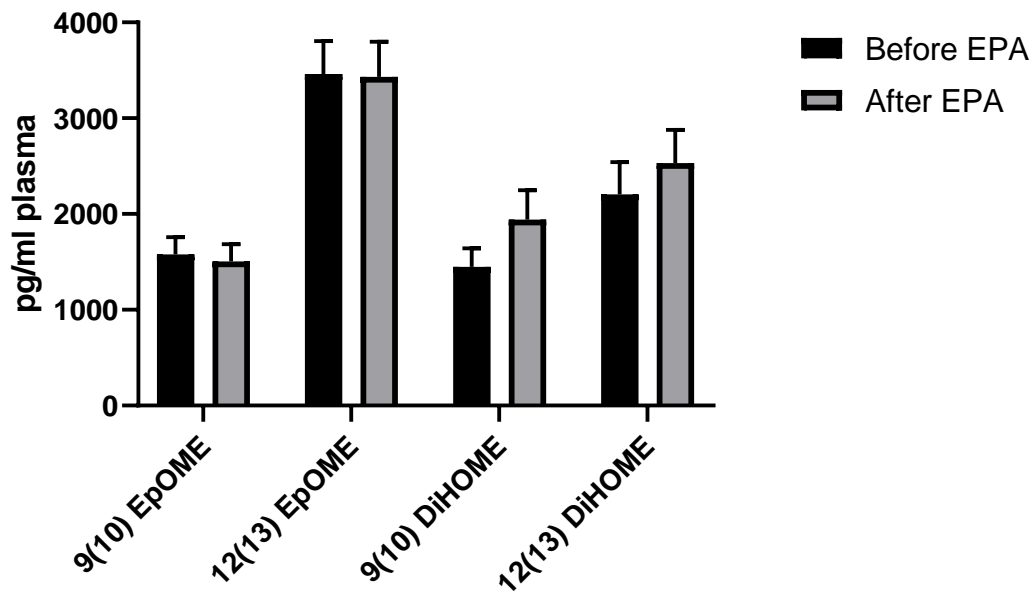

i)

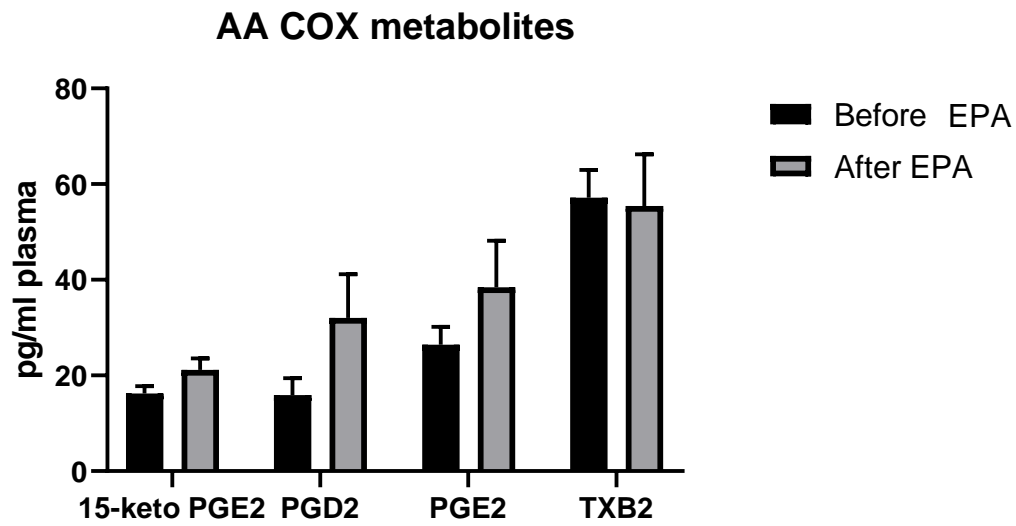

j)

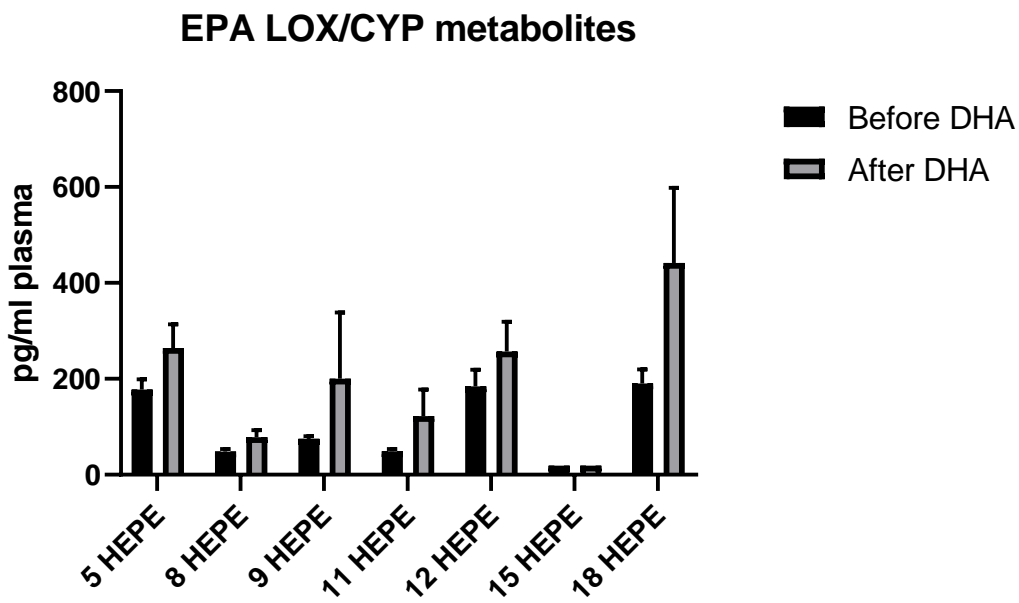

k)

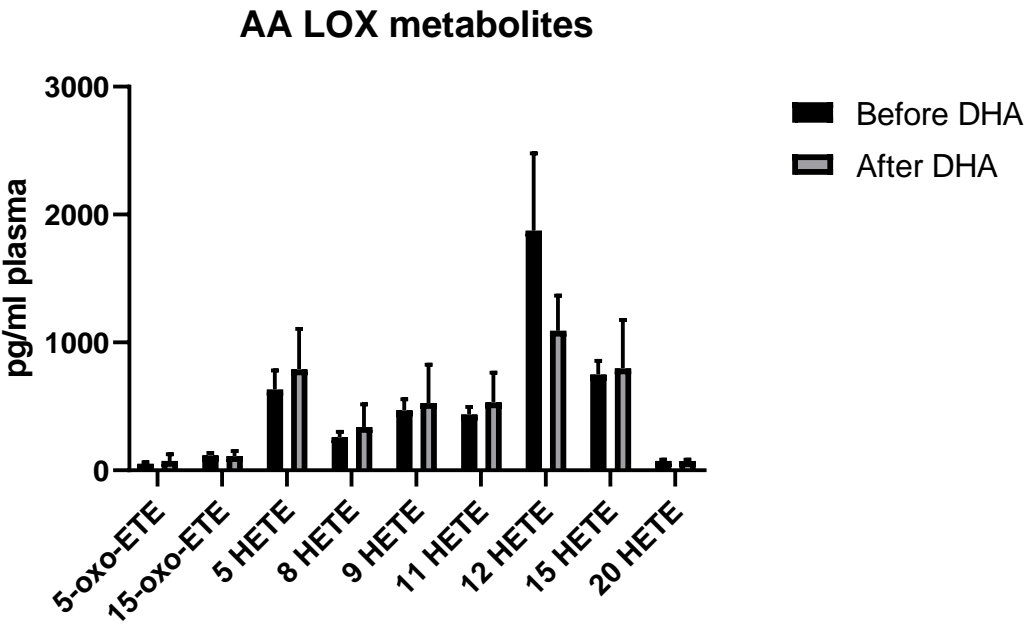

l)

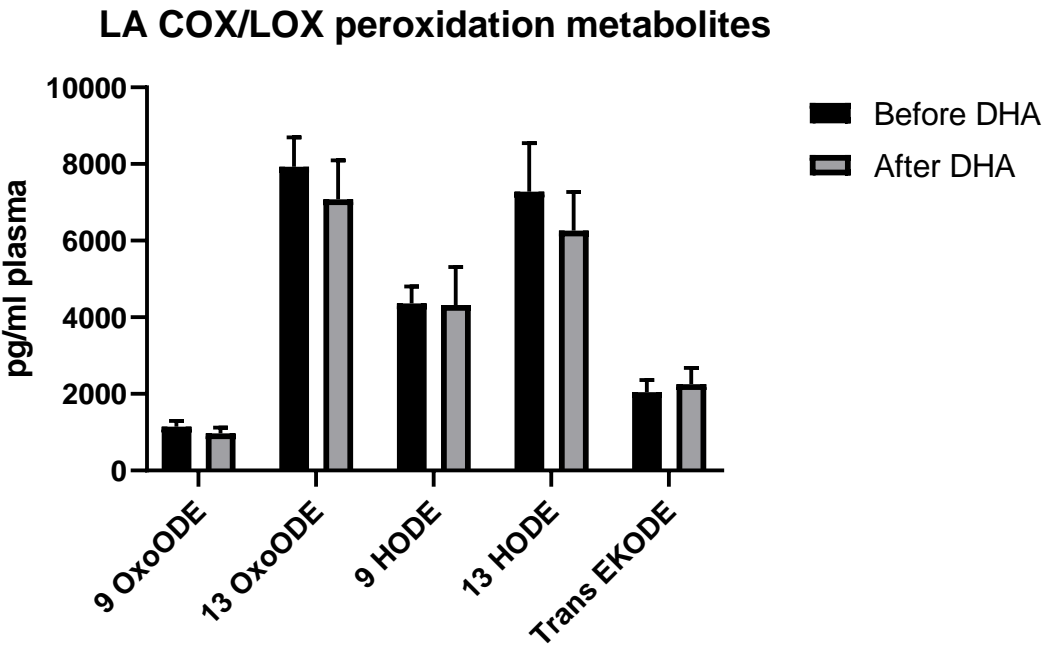

m)

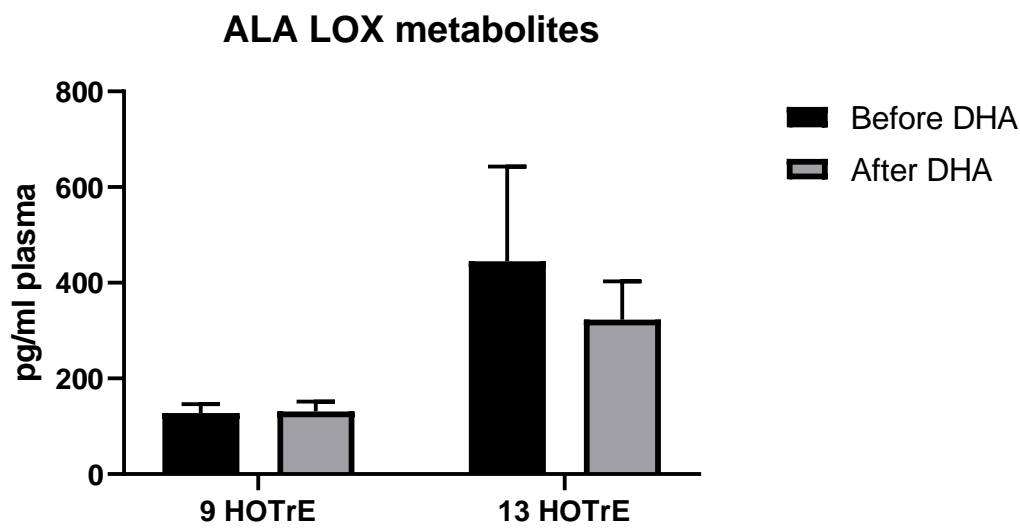

n)

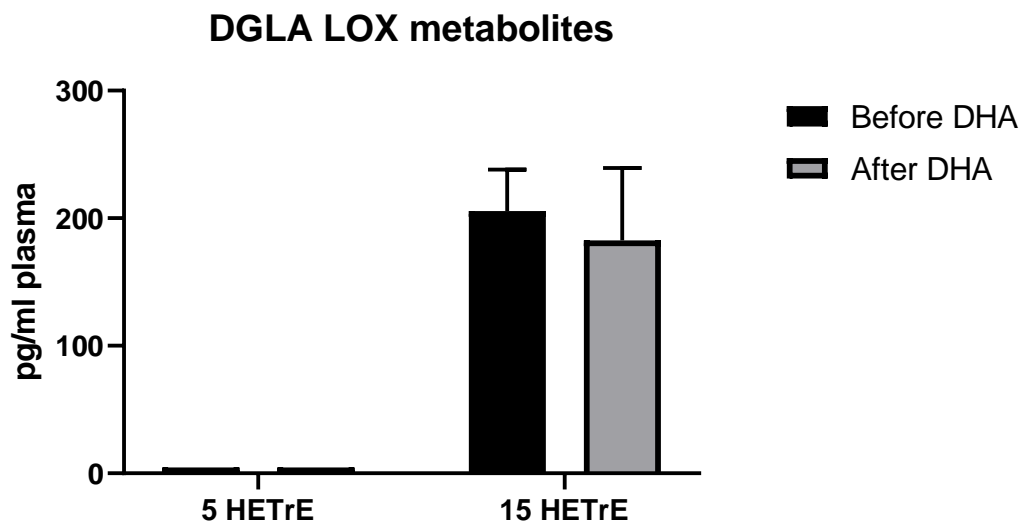

o)

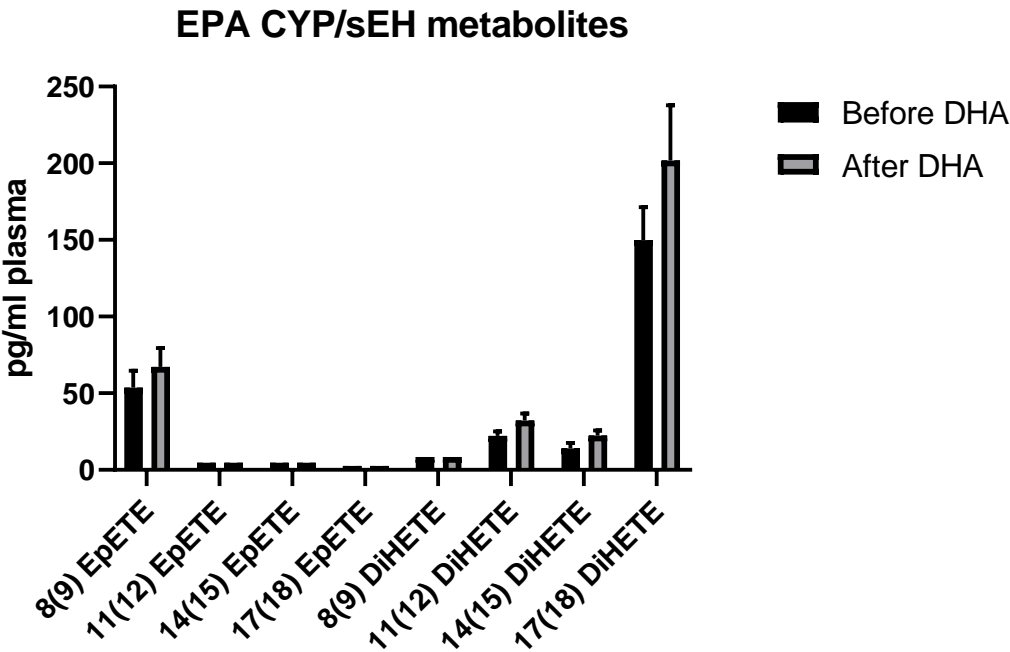

p)

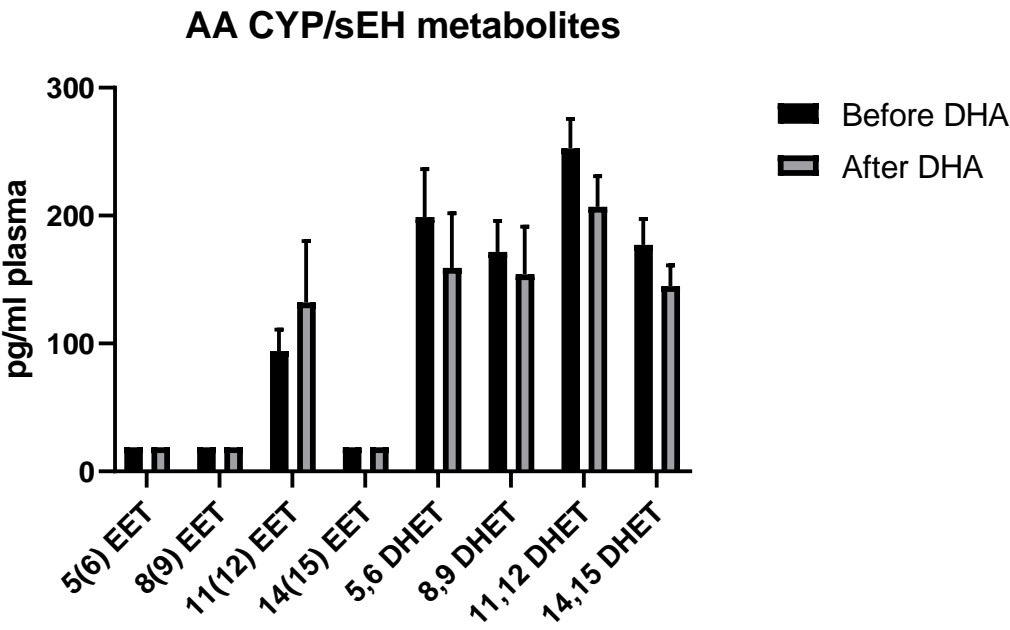

q)

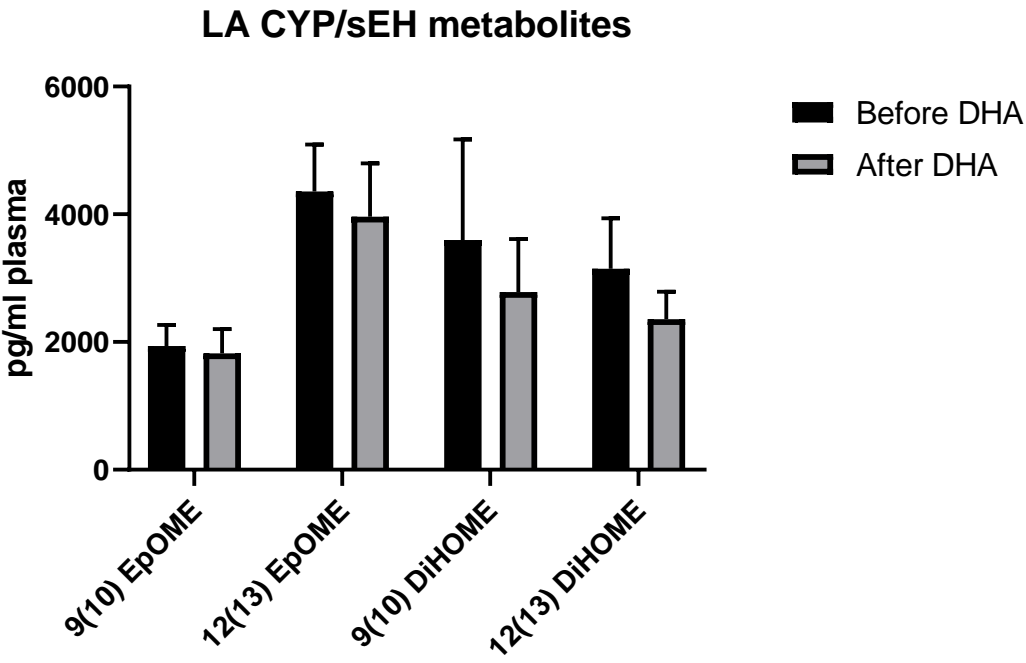

r)

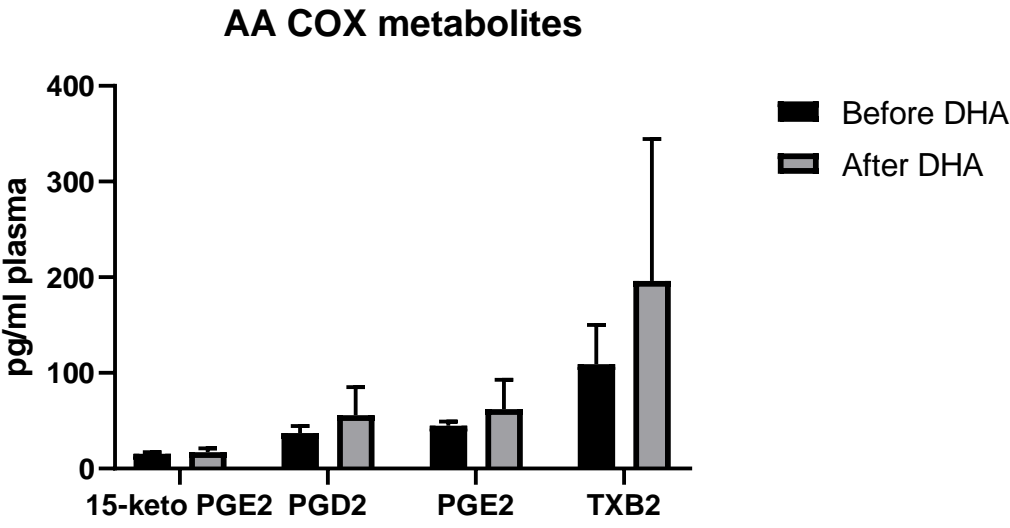

Supplement: Supplementary file 9 — Supplementary Figure 8 [file 41380_2021_1160_MOESM9_ESM.pdf]

a)

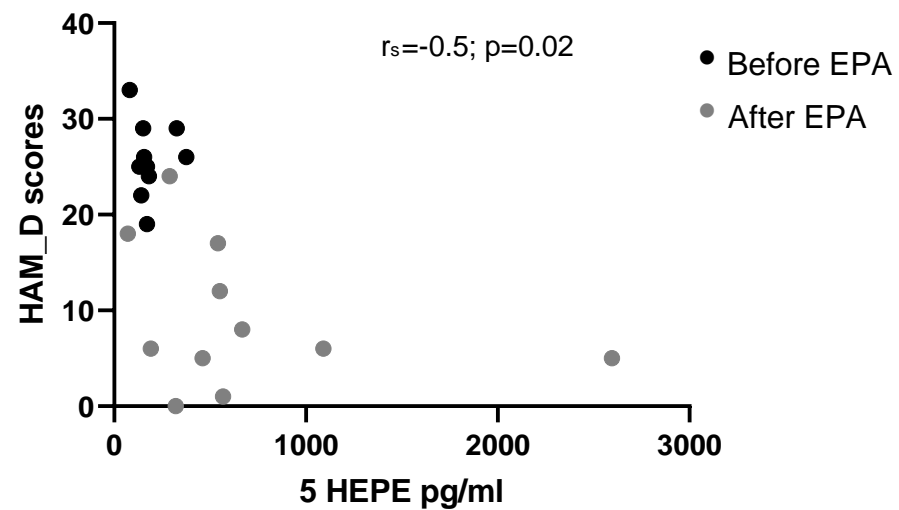

b)

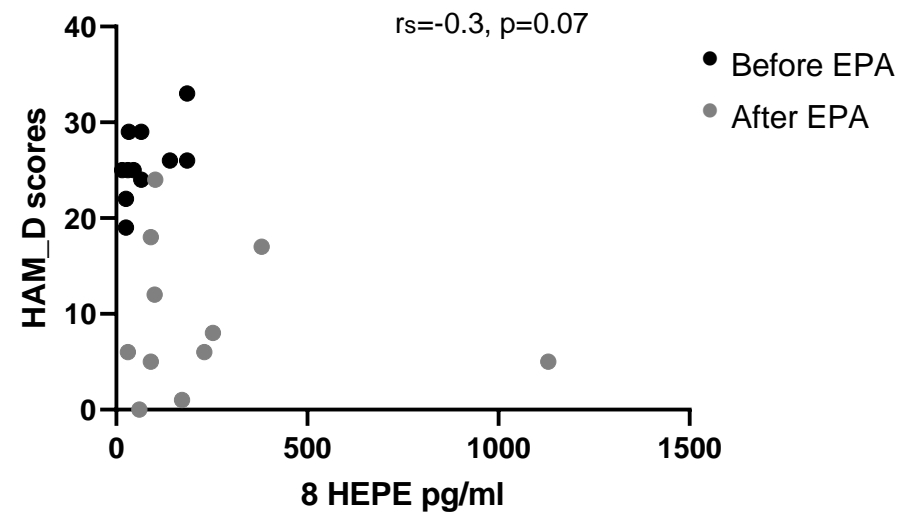

c)

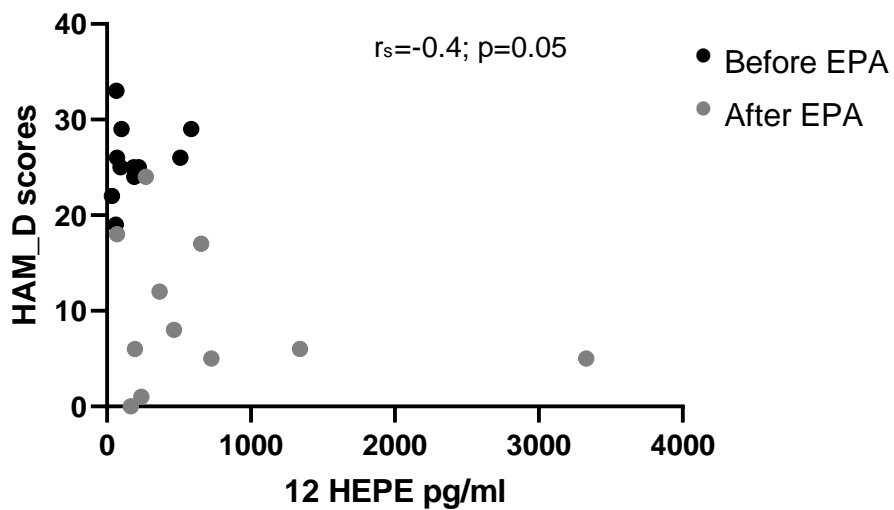

d)

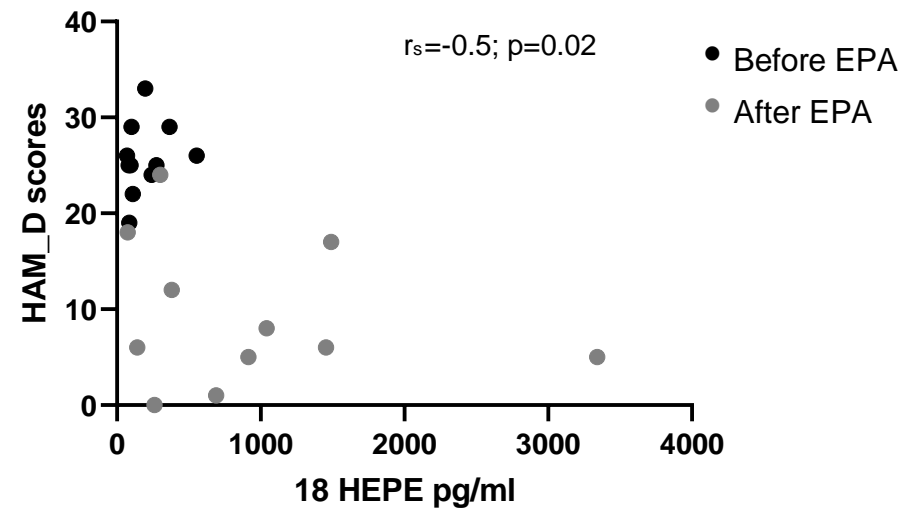

e)

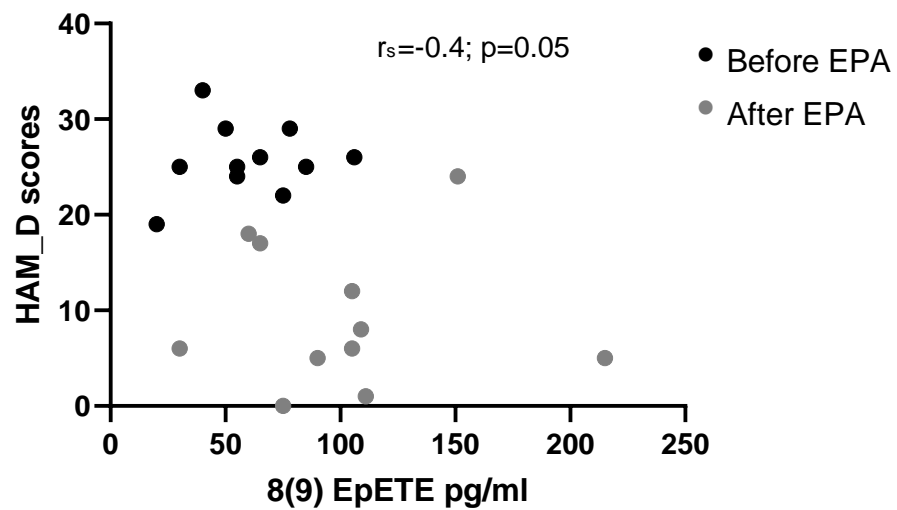

f)

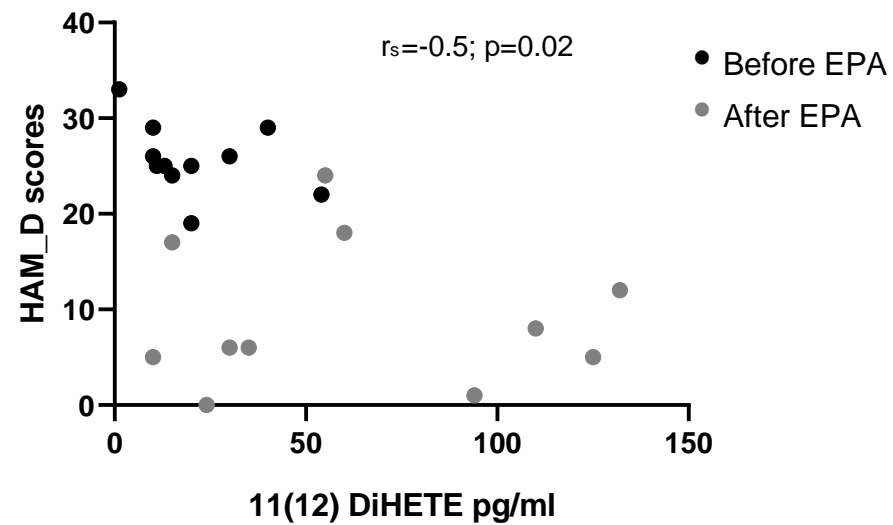

g)

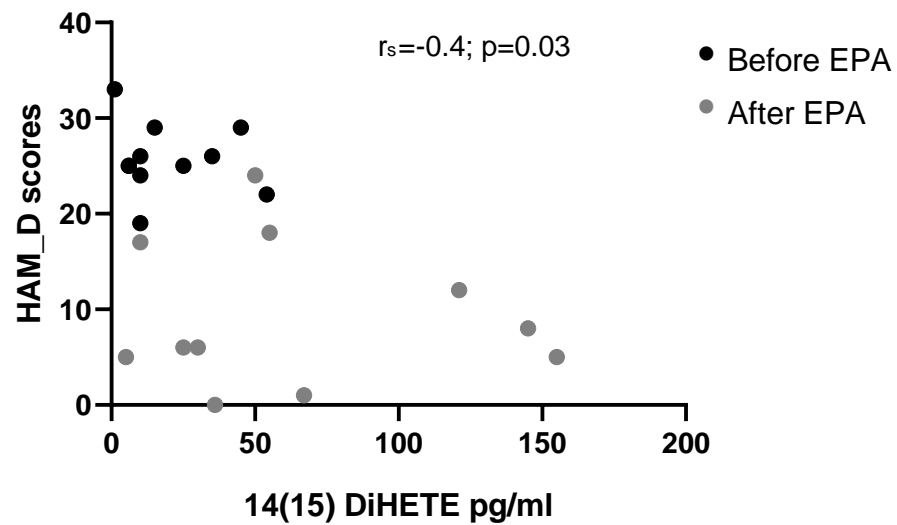

h)

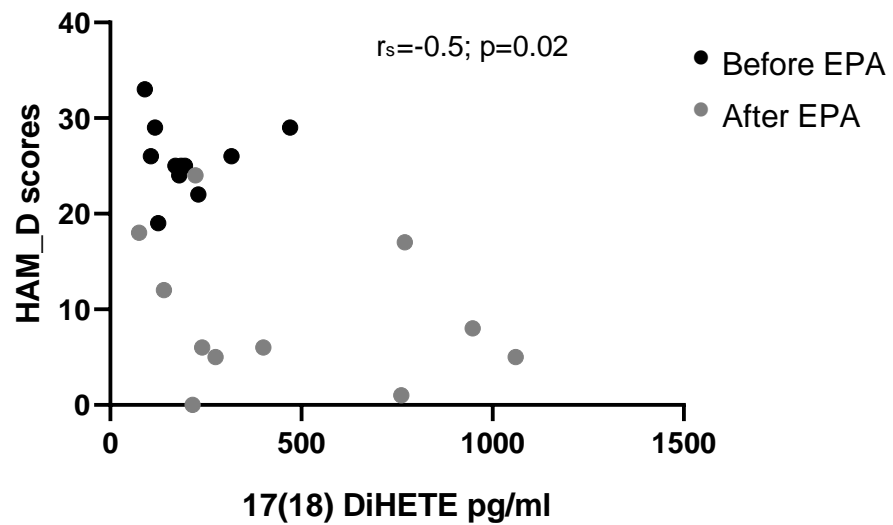

i)

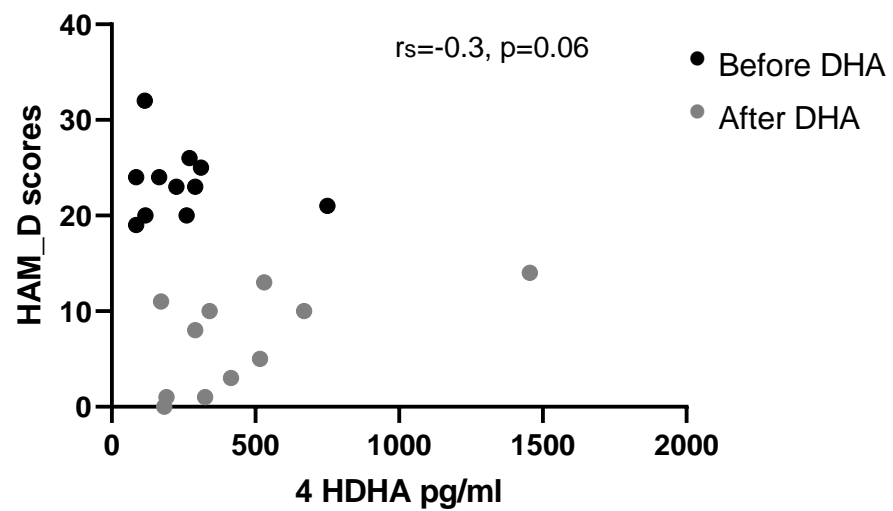

j)

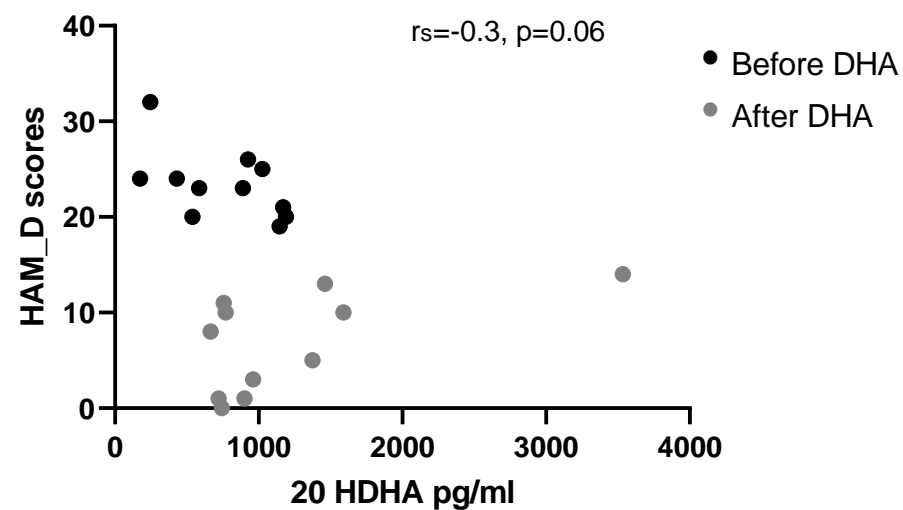

k)

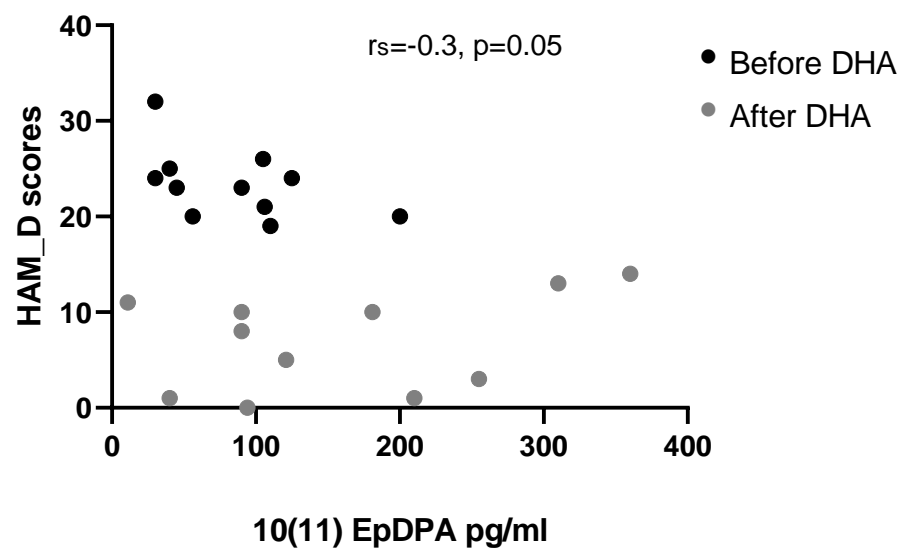

l)

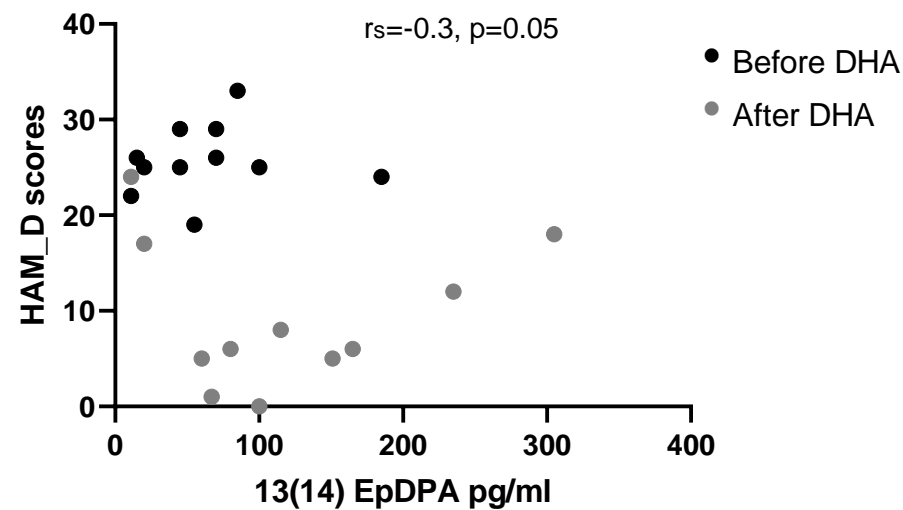

m)

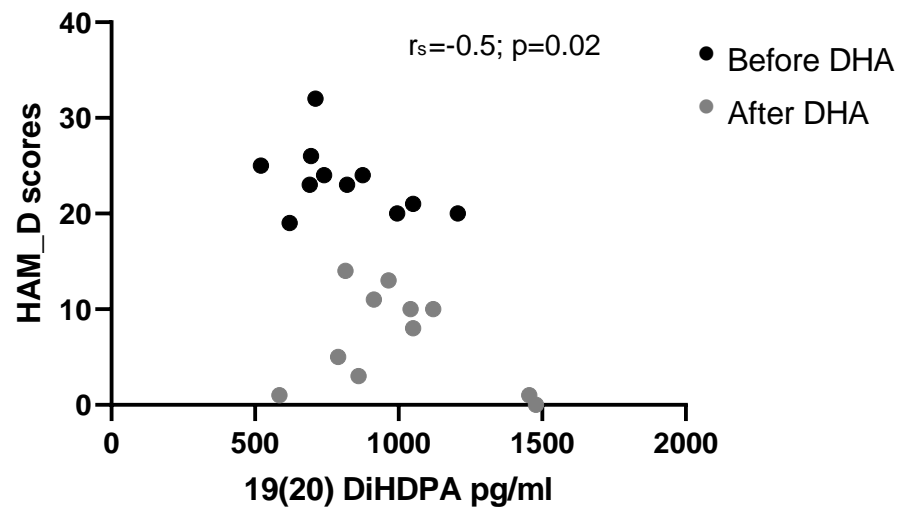

Supplement: Supplementary file 10 — Supplementary Figure 9 [file 41380_2021_1160_MOESM10_ESM.pdf]
