# Supplementary material for: Omega-3 polyunsaturated fatty acids protect against inflammation through production of LOX and CYP450 lipid mediators: relevance for major depression and for human hippocampal neurogenesis
Source: Mol Psychiatry. 2021 Jun 16;26(11):6773–88. doi: 10.1038/s41380-021-01160-8 (PMC8760043; doi:10.1038/s41380-021-01160-8)
Supplement: Supplementary file 3 — Supplementary Figure 2 [file 41380_2021_1160_MOESM3_ESM.pdf]

a)

**LA-derived metabolites**	**AA-derived metabolites**	**EPA-derived metabolites**	**DHA-derived metabolites**
9-HODE	PGD$_2$	LTB$_5$	MaR1
13-HODE	PGE$_2$	LXA$_5$	MaR2
9,10-DiHOME	PGF$_{2\alpha}$	RvE$_1$	RvD$_1$
12,13-DiHOME	PGJ$_2$	PGD$_3$	RvD$_2$
9-OxoODE	15-keto PGE$_2$	PGE$_3$	RvD$_3$
13-OxoODE	13,14-dihydro-15-keto PGE$_2$	PGF$_{3\alpha}$	RvD$_4$
9(10)-EpOME	8-iso PGF$_{2\alpha}$	TXB$_3$	RvD$_5$
12(13)-EpOME	13,14-dihydro PGF$_{2\alpha}$	5-HEPE	10(S)17(S)-DiHDPA (PDX)
Trans-EKODE	13,14-dihydro-15-keto PGF$_{2\alpha}$	8-HEPE	4-HDHA
	Δ12-PGJ$_2$	9-HEPE	7-HDHA
	15-deoxy-Δ12,14-PGJ$_2$	11-HEPE	8-HDHA
**ALA-derived metabolites**	6-keto PGF$_{1\alpha}$	12-HEPE	10-HDHA
9-HOTrE	TXB$_2$	15-HEPE	11-HDHA
13-HOTrE	LTB$_4$	18-HEPE	13-HDHA
	LXA$_4$	8(9)-EpETE	14-HDHA
	5-HETE	11(12)-EpETE	17-HDHA
**DGLA-derived metabolites**	8-HETE	14(15)-EpETE	20-HDHA
5-HETrE	9-HETE	17(18)-EpETE	10(11)-EpDPA
15-HETrE	11-HETE	8,9-DiHETE	13(14)-EpDPA
13,14-dihydro PGF$_{1\alpha}$	12-HETE	11,12-DiHETE	16(17)-EpDPA
PGD$_1$	15-HETE	14,15-DiHETE	19(20)-EpDPA
PGE$_1$	20-HETE	17,18-DiHETE	10,11-DiHDPA
PGF$_{1\alpha}$	5,6-DHET		13,14-DiHDPA
13,14-dihydro-15-keto PGE$_1$	8,9-DHET		16,17-DiHDPA
13,14-dihydro-15-keto PGF$_{1\alpha}$	11,12-DHET		19,20-DiHDPA
13,14-dihydro PGE$_1$	14,15-DHET		
	5,15-DiHETE		
	8,15-DiHETE		
	5(6)-EET		
	8(9)-EET		
	11(12)-EET		
	14(15)-EET		
	5-oxo-ETE		
	15-oxo-ETE		

b)

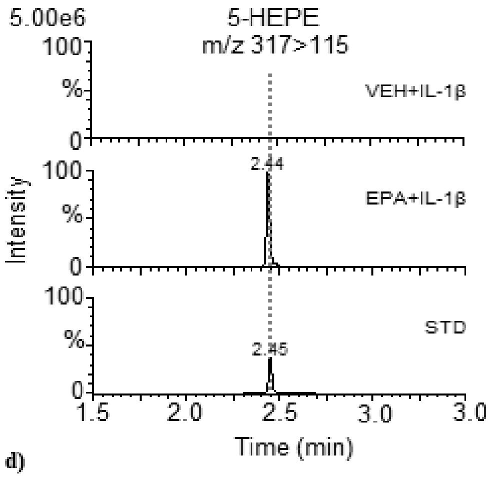
5.00e6
5-HEPE
m/z 317>115
VEH+IL-1β
EPA+IL-1β
STD
2.44
2.45
Intensity
Time (min)

c)

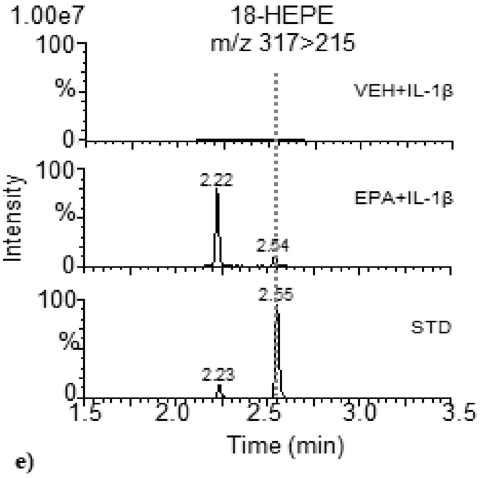
1.00e7
18-HEPE
m/z 317>215
VEH+IL-1β
EPA+IL-1β
STD
2.22
2.54
2.55
2.23
Intensity
Time (min)

d)

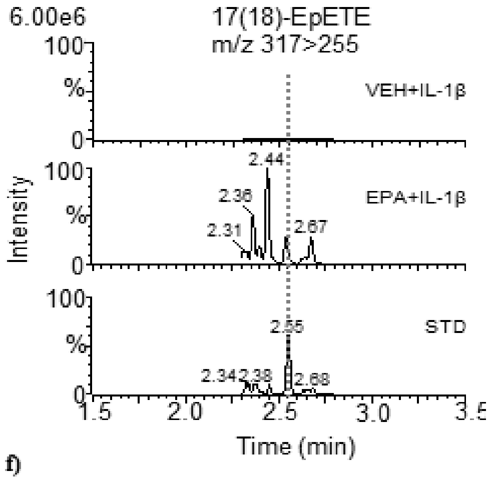
6.00e6
17(18)-EpETE
m/z 317>255
VEH+IL-1β
EPA+IL-1β
STD
2.44
2.36
2.31
2.67
2.55
2.342.38
2.68
Intensity
Time (min)

e)

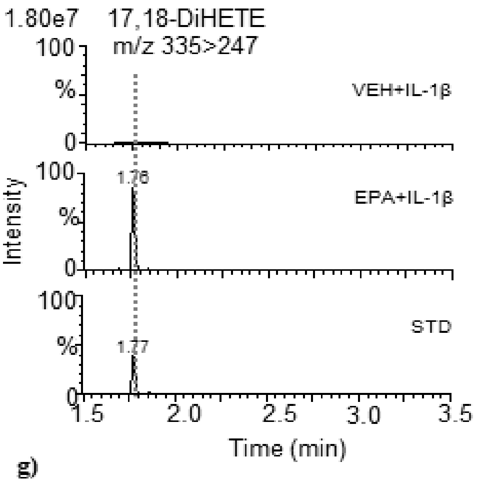
1.80e7
17,18-DiHETE
m/z 335>247
VEH+IL-1β
EPA+IL-1β
STD
1.76
1.77
Intensity
Time (min)

f)

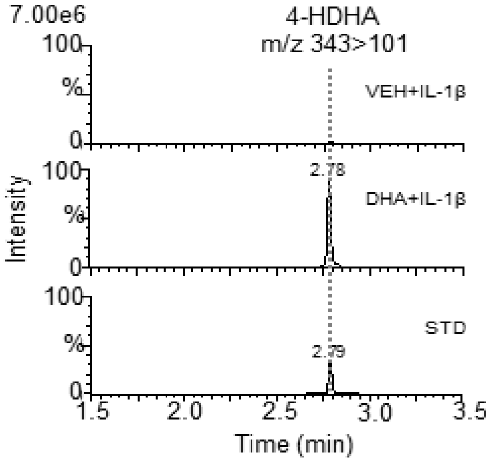
7.00e6
4-HDHA
m/z 343>101
VEH+IL-1β
DHA+IL-1β
STD
2.78
2.79
Intensity
Time (min)

g)

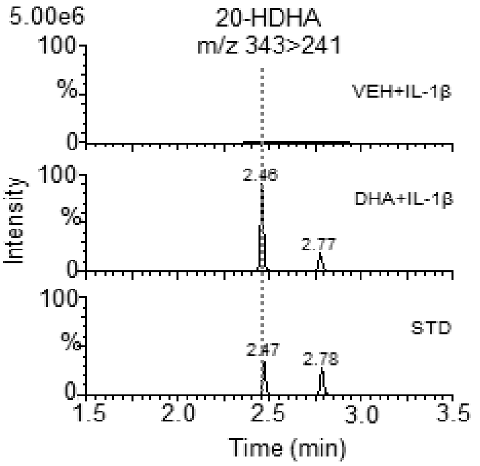
5.00e6
20-HDHA
m/z 343>241
VEH+IL-1β
DHA+IL-1β
STD
2.46
2.77
2.47
2.78
Intensity
Time (min)

h)

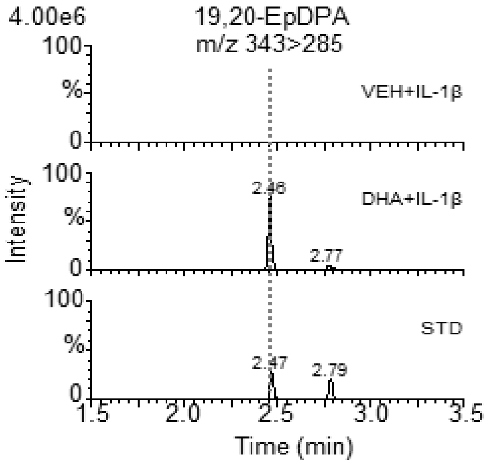
4.00e6
19,20-EpDPA
m/z 343>285
VEH+IL-1β
DHA+IL-1β
STD
2.46
2.77
2.47
2.79
Intensity
Time (min)

i)

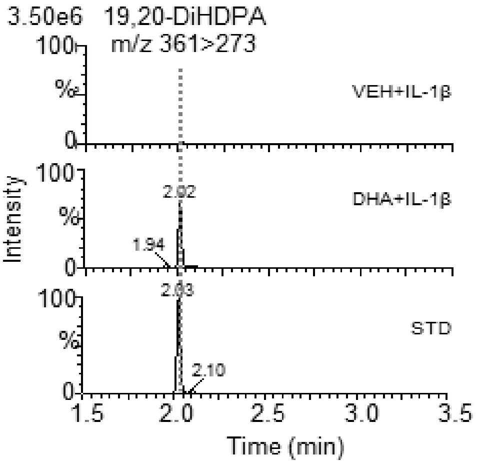
3.50e6
19,20-DiHDPA
m/z 361>273
VEH+IL-1β
DHA+IL-1β
STD
2.02
1.94
2.03
2.10
Intensity
Time (min)
